# Supplementary material for: Multiple redox switches of the SARS-CoV-2 main protease in vitro provide opportunities for drug design
Source: Nat Commun. 2024 Jan 9;15:411. doi: 10.1038/s41467-023-44621-0 (PMC10776599; doi:10.1038/s41467-023-44621-0)
Supplement: Supplementary file 1 — Supplementary Information [file 41467_2023_44621_MOESM1_ESM.pdf]

Supplementary Figures 1-36 and Supplementary Tables 1-6

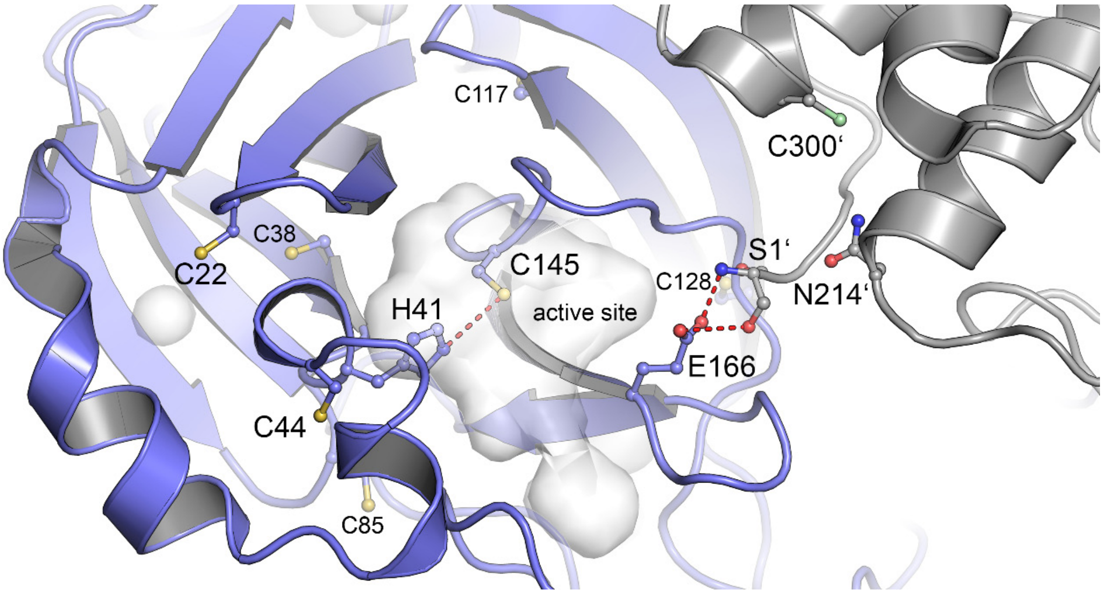

Supplementary Figure 1. Structure of M^{pro} showing the positions of cysteine residues in a monomer and at the dimer interface (pdb code 7KPH). The two monomers of the functional dimer and corresponding cysteines are colored individually. Residues contributed by the second monomer are marked with an apostrophe. The transparent volumes indicate cavities including the active site pocket. Important hydrogen-bond interactions are highlighted including that between catalytic residues C145 and H41 as well as that between E166 and N-terminal residue S1' at the dimer interface. The structure of the dimer is shown in Figure 1a of the main manuscript.

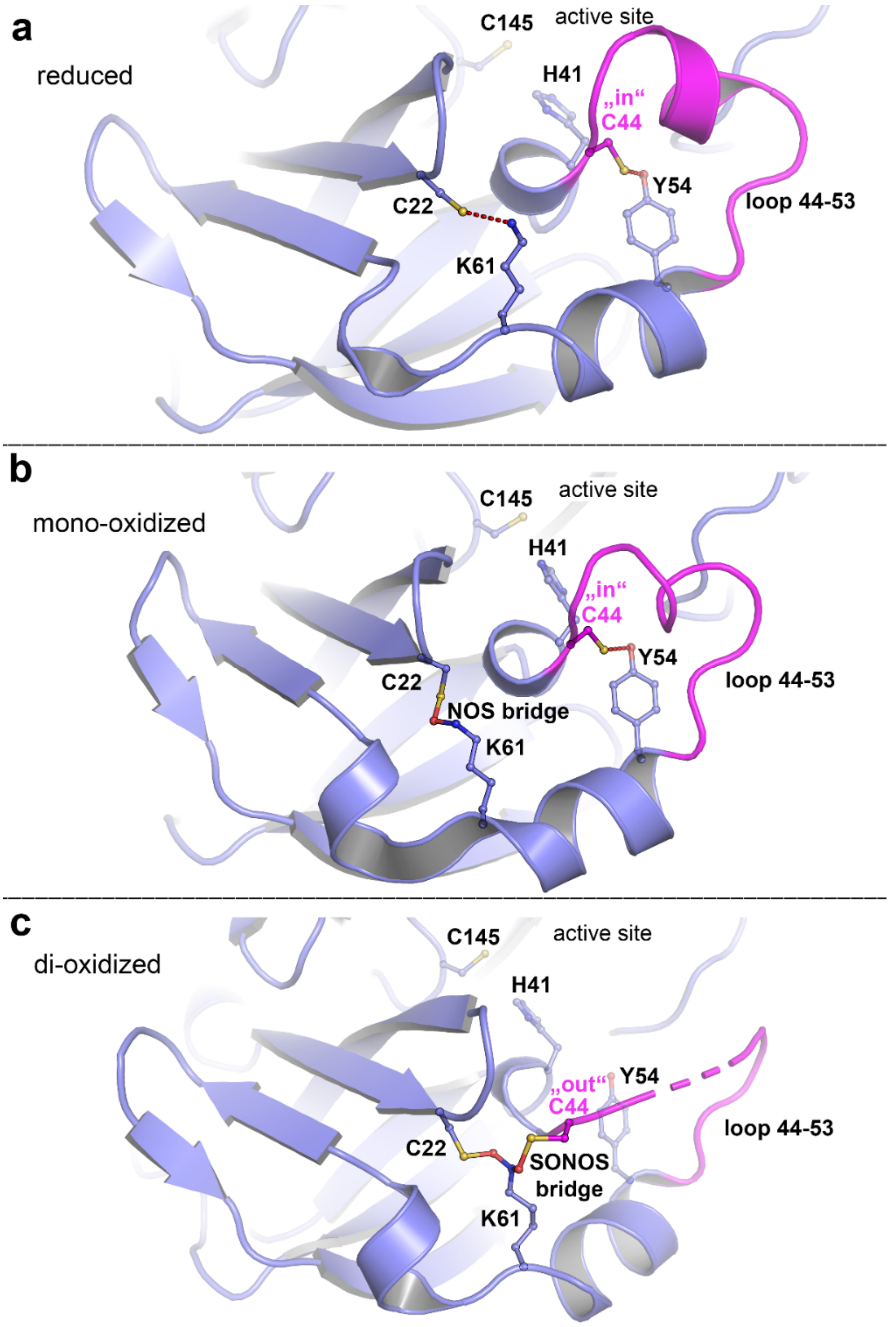

Supplementary Figure 2. NOS and SONOS bridges in the main protease M^{pro} from SARS-CoV-2. (**a**) Structure of M^{pro} in the reduced state (pdb code 7JR3) showing the redox switch at the protein surface formed by residues C22, C44 and K61 as well as the active site with residues C145 (catalytic nucleophile), H41 and Y54. A mobile loop bearing C44 is indicated in magenta. Note that C44 is in the "in" conformation and interacts with Y54. (**b**) Structure of M^{pro} in a mono-oxidized state with an NOS bridge formed between C22 and K61 (pdb code 6XMK). C44 is found in the "in" conformation. (**c**) Structure of M^{pro} in a di-oxidized state with a SONOS bridge formed between C22, K61 and C44 (pdb code 7JR4). C44 is found in the "out" conformation.

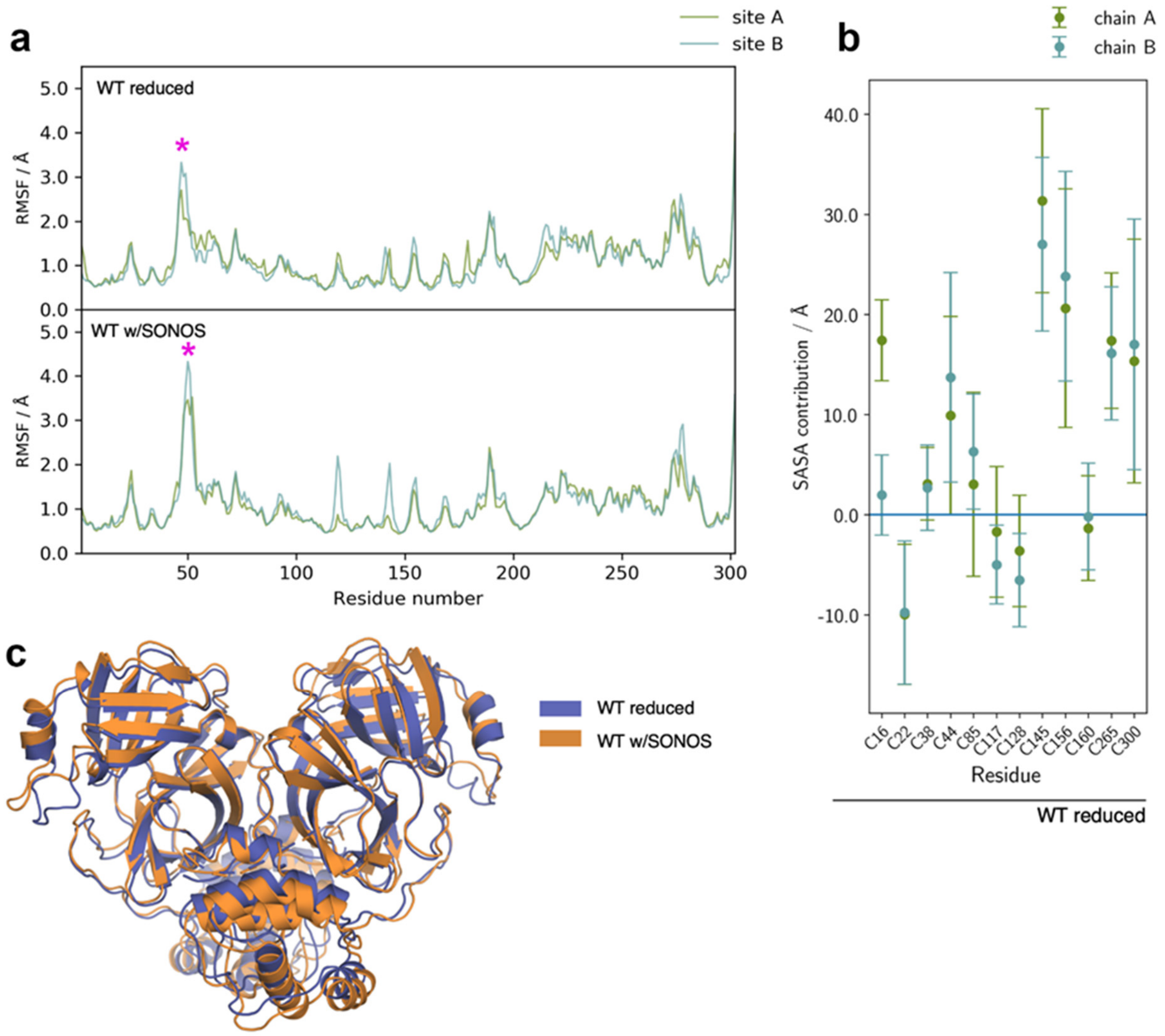

Supplementary Figure 3. Structural dynamics of M^pro as analyzed by MD simulations. (**a**) Root-mean-square fluctuations extracted from the molecular dynamics trajectories for the WT M^pro in its reduced state (top) and with a SONOS bridge (bottom). The flexible loop 44-53 (highlighted by an asterisk in magenta) is visible and both structures show very similar fluctuation patterns. (**b**) Solvent accessible surface area (SASA) contributions of individual cysteines to the full SASA of the system, following the linear combinations of pairwise overlaps (LCPO) method of Weiser et al. [SI 1]. Positive contributions show cysteines which are exposed to solvent and do not hinder neighboring residues. Negative values indicate cysteines which are blocked or effectively block from solvent contact other residues, with a net result of reducing solvent exposure. The values are based on the molecular dynamics simulations of the reduced WT. (**c**) Root-mean-square difference overlap using the alpha-carbon positions for the averaged MD structures of the reduced WT and with a SONOS bridge (RMSD of 1.44 Å). Both structures are very close to identical with the exception of the 44-53 loop.

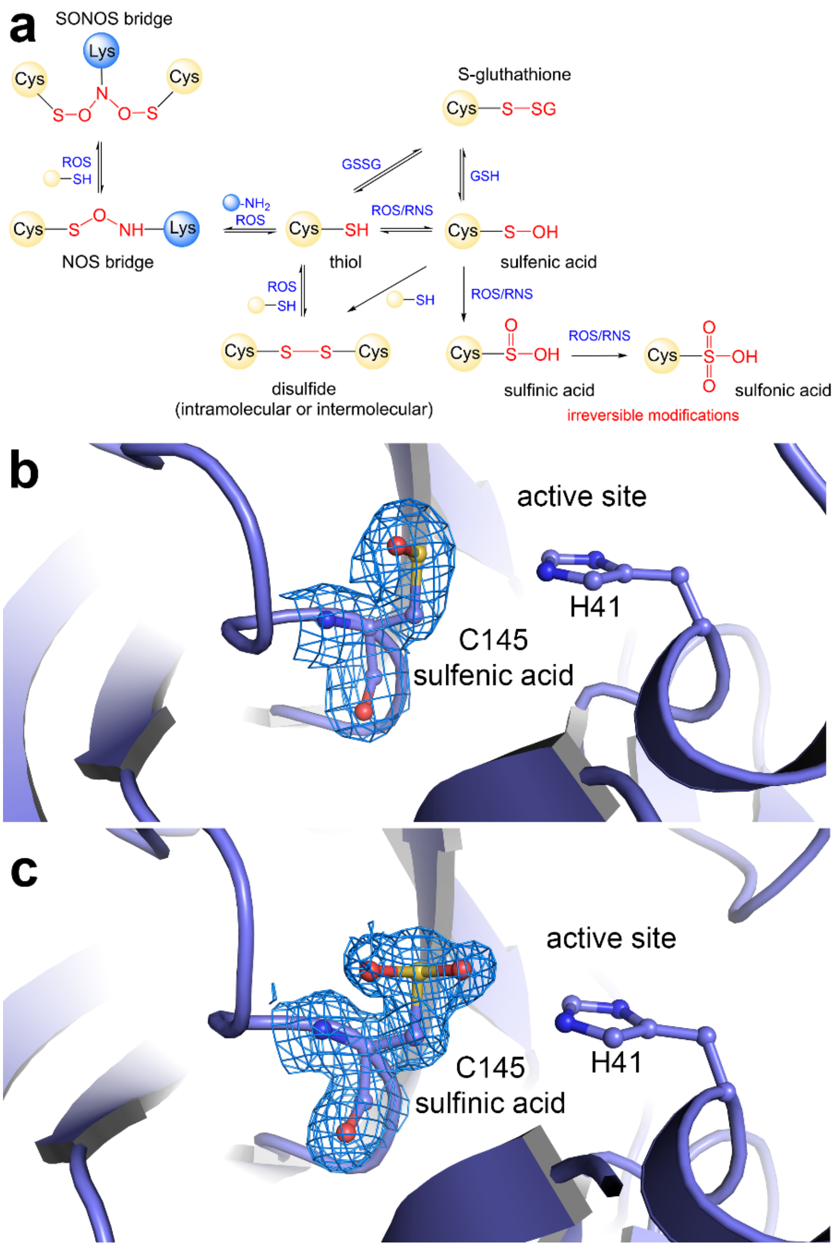

Supplementary Figure 4. Redox modifications of the catalytic cysteine C145 in M^{pro} from SARS-CoV-2. (**a**) Redox modifications of cysteines in proteins showing key species involved in oxidative and reductive transformations. Abbreviations: Cys, cysteine; Lys, lysine; ROS, reactive oxygen species; RNS, reactive nitrogen species; GSH, glutathione; GSSG, glutathione disulfide. (**b**) Structure of M^{pro} with catalytic C145 in the mono-oxidized sulfenic acid state (pdb code 6XKF). The structural model of C145 is superposed with the 2mFo-DFc electron density map at a contour level of 1σ. (**c**) Structure of M^{pro} with catalytic C145 in the di-oxidized sulfinic acid state (pdb code 6XKH). The structural model of C145 is superposed with the 2mFo-DFc electron density map at a contour level of 1σ.

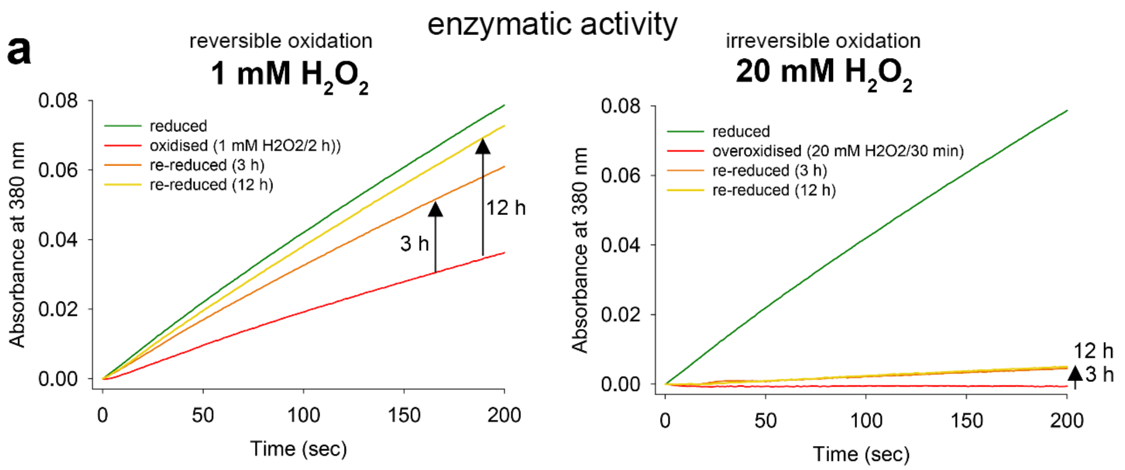

	reduced	(over)oxidized	re-reduced (3h)	re-reduced (12h)
WT (oxidation)	100.00%	33.45%	69.79%	83.92%
WT (overoxidation)	100.00%	0.13%	4.43%	4.93%

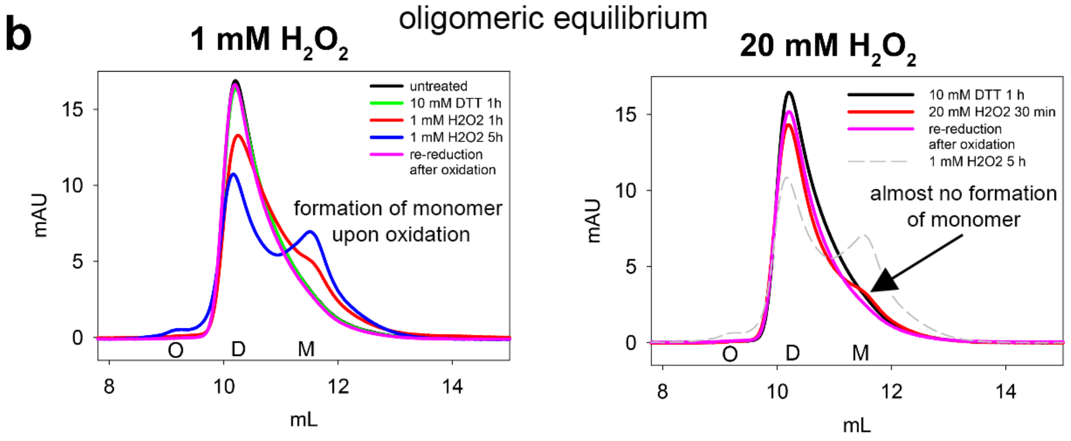

Supplementary Figure 5. Redox-dependent enzymatic activity and oligomeric equilibrium of M^{pro}. (**a**) Progress curves of M^{pro}-catalyzed substrate turnover in the reduced state, after oxidation with either 1 mM H_2O_2 (left panel) or 20 mM H_2O_2 (right panel) and after re-reduction with reductant DTT. Experimental details are provided in the Supplementary Methods. The estimated relative enzymatic activities are summarized in the accompanying table. An activity of 100% refers to that of the reduced enzyme. Note that an oxidation with 20 mM H_2O_2 leads to an irreversible inactivation of M^{pro}, while treatment with 1 mM H_2O_2 entails a reversible loss of activity. (**b**) Gel filtration analysis of the oligomeric state of M^{pro} wild-type in the reduced state, after different reaction times with either 1 mM H_2O_2 (left panel) or 20 mM H_2O_2 (right panel) and after re-reduction. Abbreviations: O, oligomer; D, dimer; M, monomer. Note the progressive formation of the monomer with increasing oxidation times after reaction with 1 mM H_2O_2. Re-reduction restores the dimer.

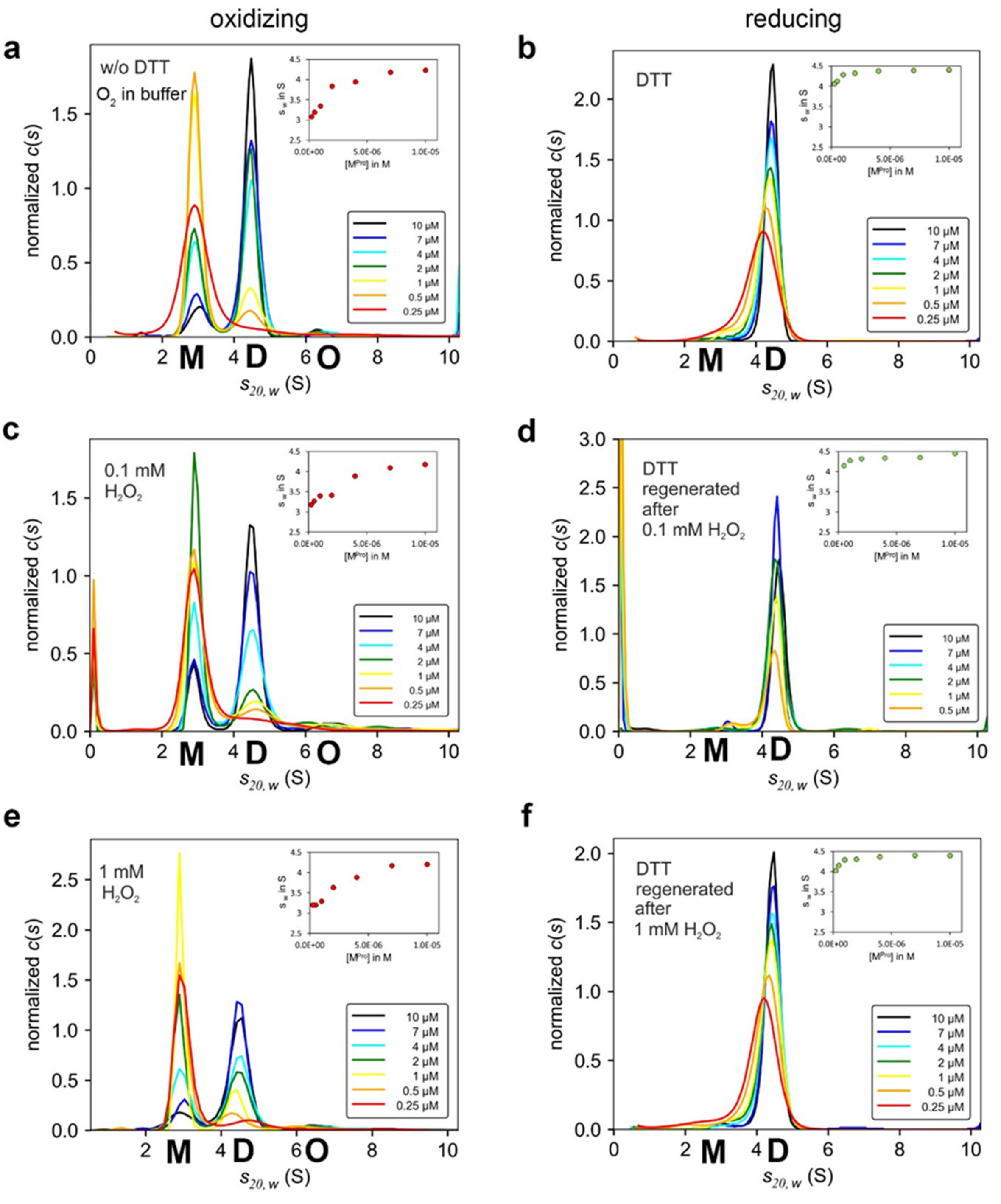

Supplementary Figure 6. Redox-dependent oligomeric equilibrium of M^{pro} from SARS-CoV-2. Sedimentation velocity analysis of SARS-CoV-2 M^{pro} in a concentration range from 0.25 to 10 µM under different oxidising (left panels **a**, **c**, **e**) or reducing (right panels **b**, **d**, **f**) conditions indicate a redox-dependent monomer ⇔ dimer equilibrium with apparent equilibrium constants of K_{app} <0.25 µM for the reduced enzyme and of about 2.5 µM for the oxidized enzyme. Insets show s_w binding isotherms, as calculated from the corresponding c(s) distributions. Abbreviations: M, monomer ($s_{20,w}$ = 2.9 S); D, dimer ($s_{20,w}$ = 4.5 S); O, oligomers (($s_{20,w}$ = 6.3 S). Note that the redox switching is reversible as re-reduction of previously oxidized M^{pro} by DTT leads to a dimerization. Analysis upon treatment with 20 mM H_2O_2 could not be conducted as the protein aggregated during the process of equilibration in the centrifuge.

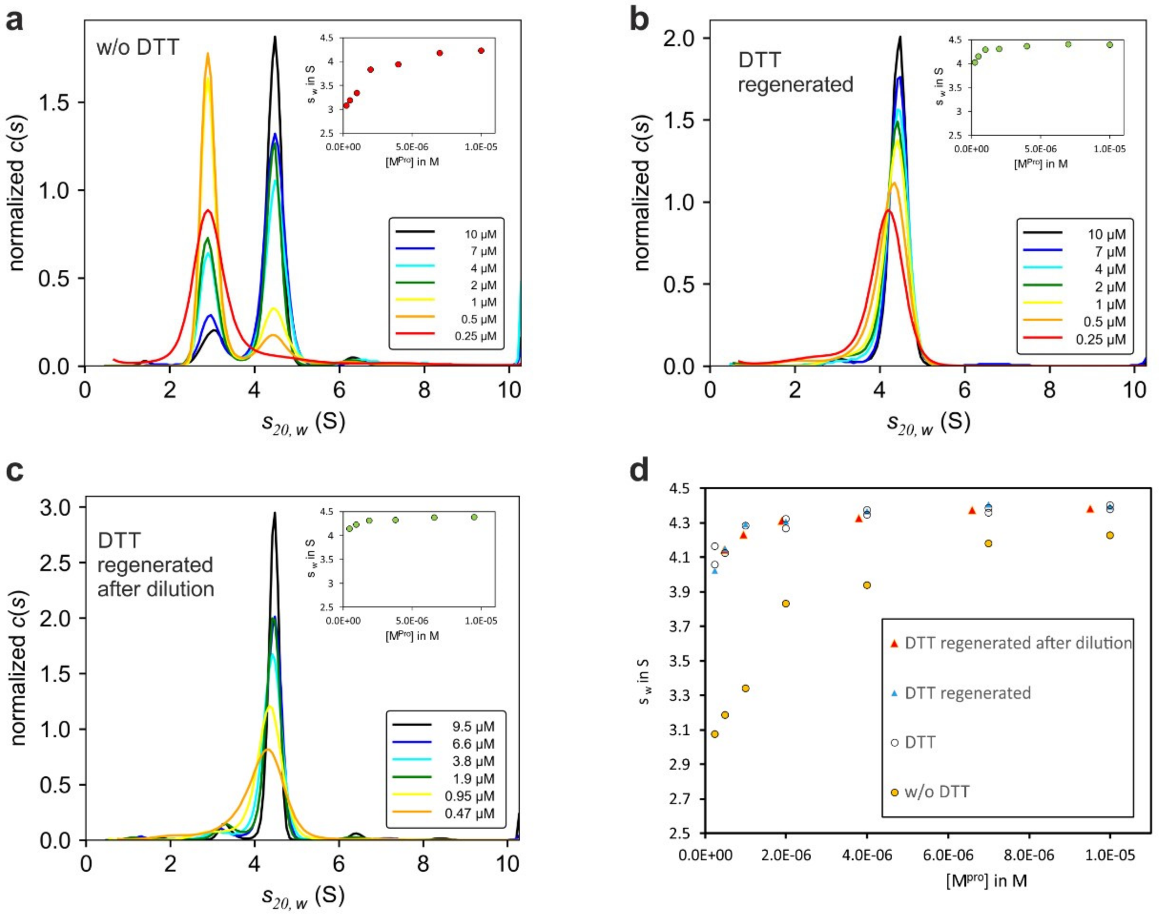

Supplementary Figure 7. Oxidation and regeneration of SARS-CoV-2 M^{pro} after removal of DTT. The corresponding c(s) distributions were obtained from sedimentation velocity analyses. Insets show s_w binding isotherms, as calculated from the corresponding c(s) distributions. (**a**) M^{pro} in non-reducing buffer (devoid of reductant DTT). Samples were diluted to 0.25 - 10 µM and analyzed by analytical ultracentrifugation (AUC) 23 h after dilution. (**b**) After overnight dialysis of M^{pro} against non-reducing buffer, protein stock solution was diluted to 0.25 -10 µM with buffer + DTT. After 23 h of further incubation at room temperature, samples were analyzed by AUC. (**c**) After overnight dialysis against non-reducing buffer, M^{Pro} was diluted with non-reducing buffer to 0.5 - 10 µM and incubated for 23 h at room temperature. Afterwards, 1 mM DTT was added and M^{Pro} was allowed to regenerate for 23 h at room temperature before AUC analysis. (**d**) Comparison of s_w isotherms obtained after M^{pro} regeneration with DTT from experiments in (b) and (c) with those obtained for M^{Pro} in buffer + DTT without previous oxidation (see Fig. 1b in the main manuscript) and in non-reducing buffer (a). M^{Pro} oxidation in the absence of DTT is completely reversible, even if the protein was already dissociated before DTT addition. For better representation, all c(s) distributions in (a)-(c) were normalized to the same area.

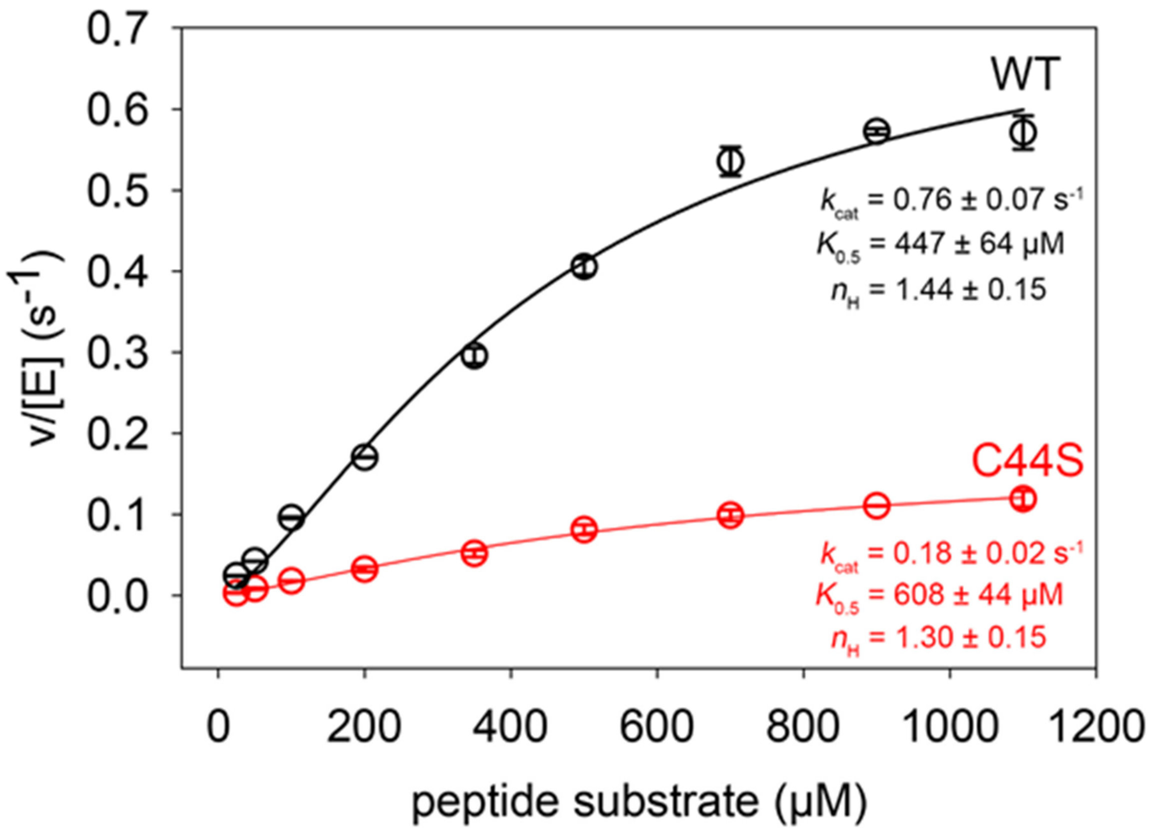

Supplementary Figure 8. Steady-state kinetic analysis of M^{pro} wild-type and variant C44S. The corresponding v_S plots for proteolytic cleavage of a peptidic model substrate are shown for the wild-type protein (WT) in black and for variant C44 in red. Experimental details are summarized in the Online Methods. All measurements were carried out in duplicate and are shown as mean ± s.d. Data were fitted with the Hill equation to obtain estimates for the catalytic constant $k_{cat,}$ the substrate binding constant $K_{0.5}$ and the Hill coefficient n_H. The fits are shown as solid lines and the estimated kinetic constants are depicted for both proteins. Note that the C44S variant exhibits a ~4-fold decreased catalytic constant relative to the WT enzyme, while the substrate affinity is only slightly changed. Both the WT as well as variant C44S exhibit positive cooperativity as signified by Hill coefficients n_H >1.

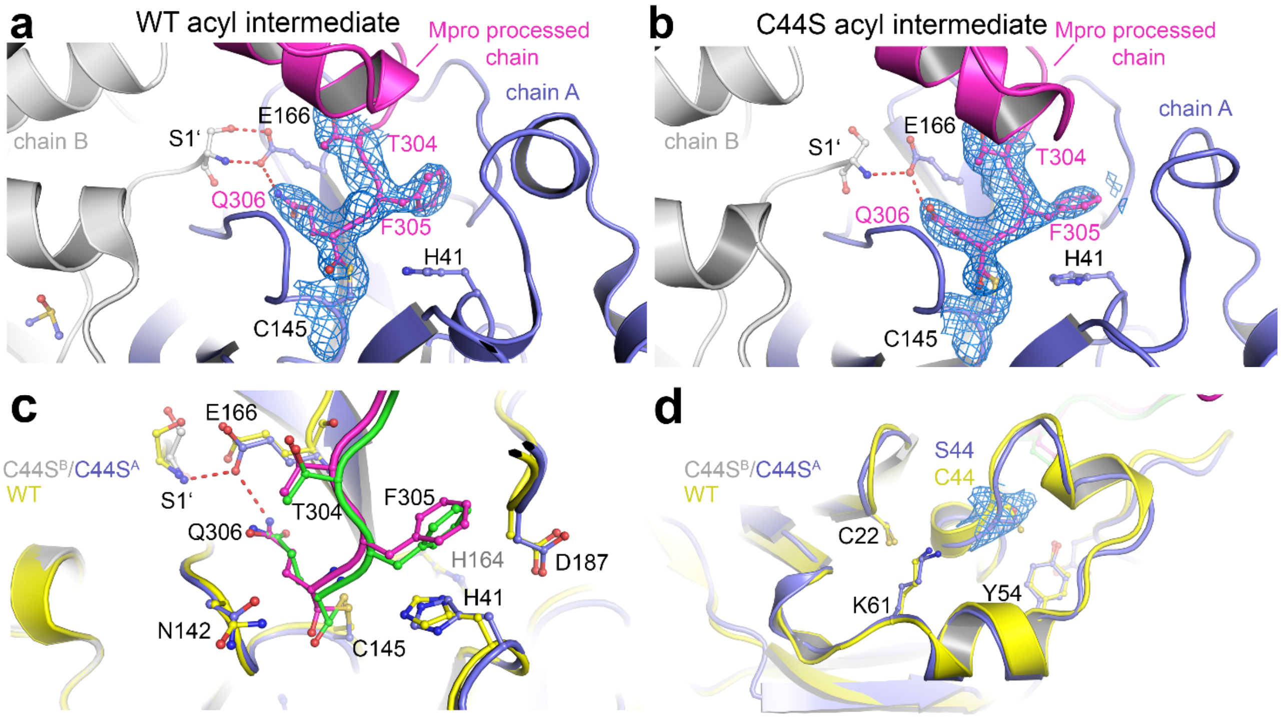

Supplementary Figure 9. X-ray crystallographic structures of the covalent acyl intermediates in M^{pro} wildtype (WT) and variant C44S. (**a**) Snapshot of the acyl intermediate in M^{pro} WT (pdb code 7KHP) formed between a functional dimer in the asymmetric unit and a symmetry-related M^{pro} molecule [39]. The two chains of the dimer are colored individually (chain A, blue; chain B, grey). Residues contributed by chain B are marked with an apostrophe. The C-terminus of the symmetry-related molecule, which visits the active site and forms a covalent linkage with catalytic C145, is highlighted in magenta. The structural model of C145 and the last three C-terminal residues of the symmetry-related molecule are superposed with the corresponding 2mFo-DFc electron density map contoured at 1σ. (**b**) Snapshot of the acyl intermediate in variant C44S (this study) formed between a functional dimer in the asymmetric unit and a symmetry-related M^{pro} molecule. The two chains of the dimer are colored individually (chain A, blue; chain B, grey). Residues contributed by chain B are marked with an apostrophe. The C-terminus of the symmetry-related molecule, which visits the active site and forms a covalent linkage with catalytic C145, is highlighted in magenta. The structural model of C145 and the last three C-terminal residues of the symmetry-related molecule are superposed with the corresponding 2mFo-DFc electron density map contoured at 1σ. (**c**) Superposition of the WT structure (yellow, symmetry-related molecule green) with that of variant C44 (blue/grey, symmetry-related molecule magenta) showing the active sites. Note the structural differences at the active site, in particular of H41, C145 and the visiting C-terminus. (**d**) Superposition of the WT structure (yellow) with that of variant C44 (blue), showing the allosteric SONOS redox switch sites. The structural model of mutation site S44 in the variant is superposed with the corresponding 2mFo-DFc electron density map. Please note that loop 43-50 bearing the mutation site exhibits increased flexibility indicated by elevated B-factors.

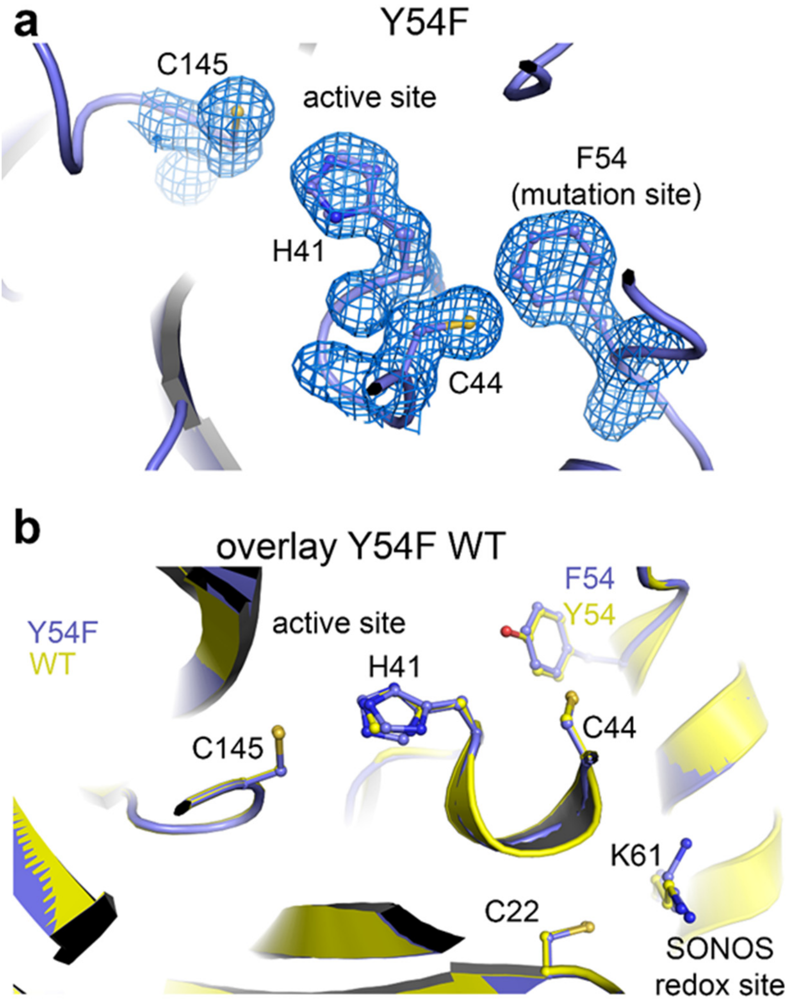

Supplementary Figure 10. X-ray crystallographic structure of M^{pro} variant Y54F. (**a**) Structure of the active site showing catalytic residues C145 and H41 as well as mutation site F54 and SONOS residue C44. The structural model is superposed with the corresponding 2mFo-DFc electron density map at a contour level of 1σ. (**b**) Superposition of the wild-type (WT) structure (yellow, pdb code 7KPH) with that of variant Y54F (blue, this study). Note the slight structural changes of catalytic residue H41 and its increased flexibility indicated by two alternate conformations.

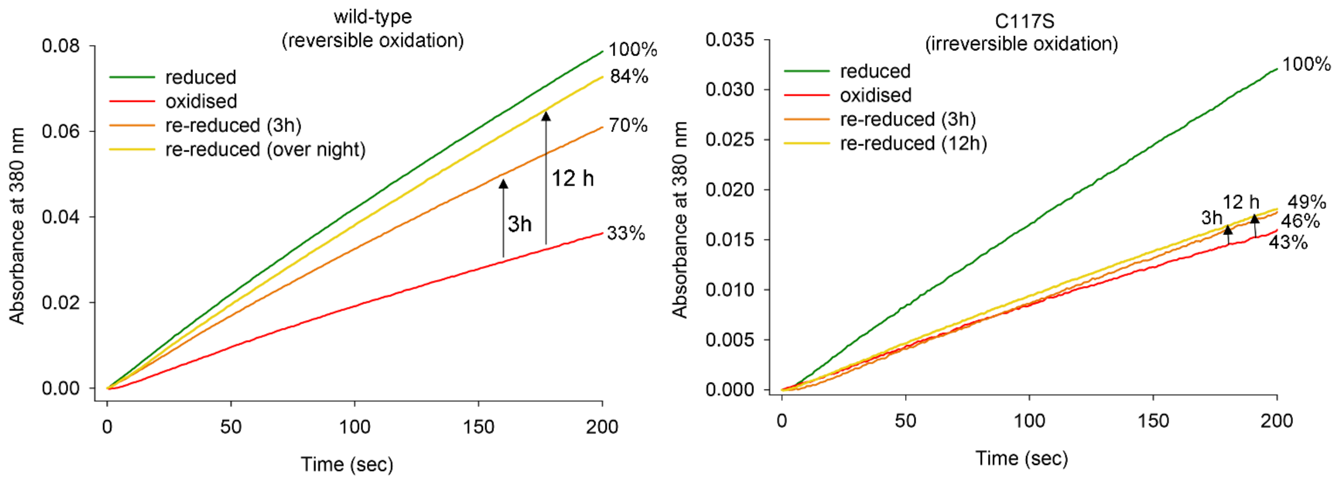

Supplementary Figure 11. Redox-dependent activity of M^{pro} wild-type (left panel) and variant C117S (right panel). Progress curves of M^{pro} catalyzed substrate turnover in the reduced state, after oxidation with 1 mM H_2O_2 and after re-reduction with reductant DTT (3 h and 12 h). Experimental details are provided in the Online Methods. An activity of 100% refers to that of the reduced enzyme. Please note that - in contrast to the wild-type protein - the loss of enzymatic activity after an oxidative insult with 1 mM H_2O_2 cannot be recovered in case of variant C117S.

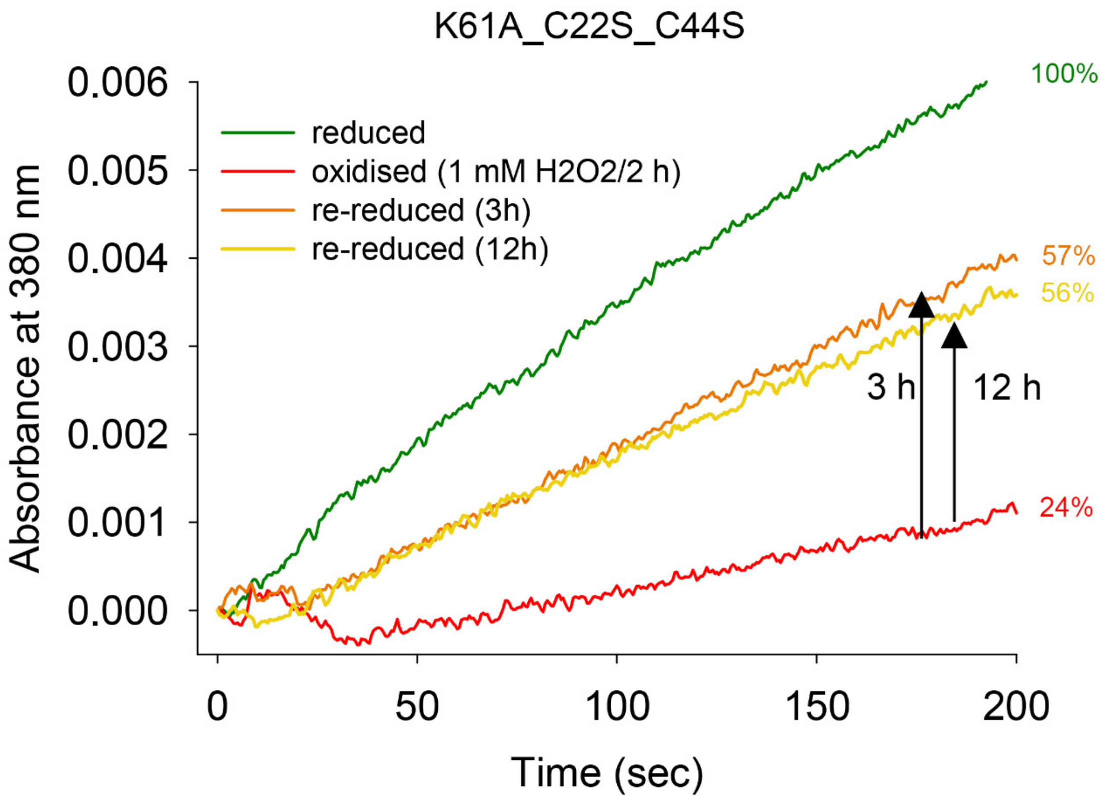

Supplementary Figure 12. Redox-dependent activity of M^{pro} triple variant K61A_C22S_C44S. Progress curves of M^{pro}-catalyzed substrate turnover in the reduced state, after oxidation with 1 mM H_2O_2 and after re-reduction with reductant DTT (3 h and 12 h). Experimental details are provided in the Online Methods. An activity of 100% refers to that of the reduced enzyme. Please note that - in contrast to the wild-type protein - the loss of enzymatic activity after an oxidative insult with 1 mM H_2O_2 cannot be fully recovered.

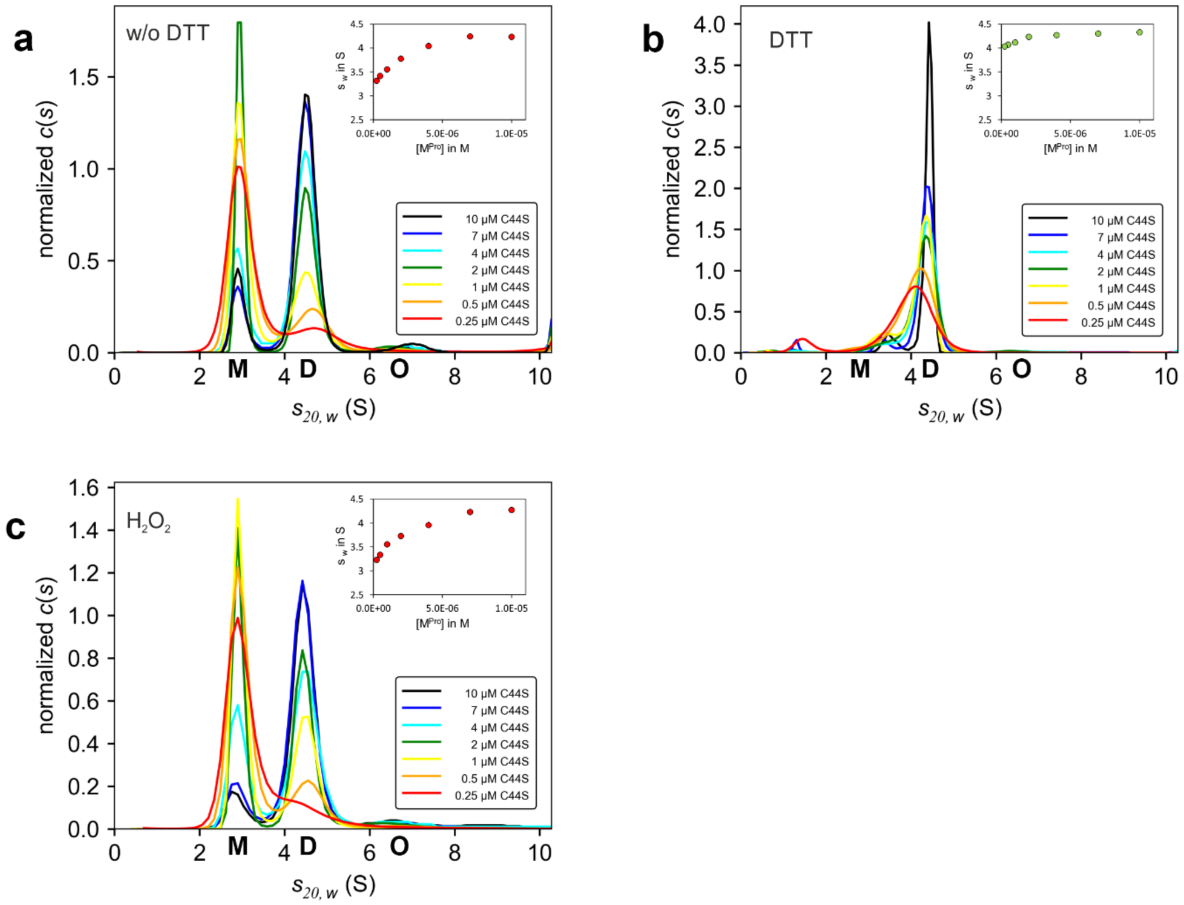

Supplementary Figure 13. Redox-dependent sedimentation velocity analysis of SARS-CoV-2 M^pro variant C44S. The oligomeric equilibrium was analyzed in a concentration range from 0.25 to 10 µM under either non-reducing (**a,** buffer devoid of DTT), reducing (**b**, buffer supplemented with 1 mM DTT) or oxidizing (**c**, 1 mM H_2O_2) conditions. The data indicate a redox-dependent monomer ⇔ dimer equilibrium with apparent equilibrium constants of K_D^{app} <0.25 µM for the reduced enzyme and about 2.5 µM for the oxidized enzyme. Insets show s_w binding isotherms, as calculated from the corresponding c(s) distributions. Abbreviations: M, monomer ($s_{20,w}$ = 2.9 S); D, dimer ($s_{20,w}$ = 4.5 S); O, oligomers ($s_{20,w}$ ≈ 6.3 S).

C117S

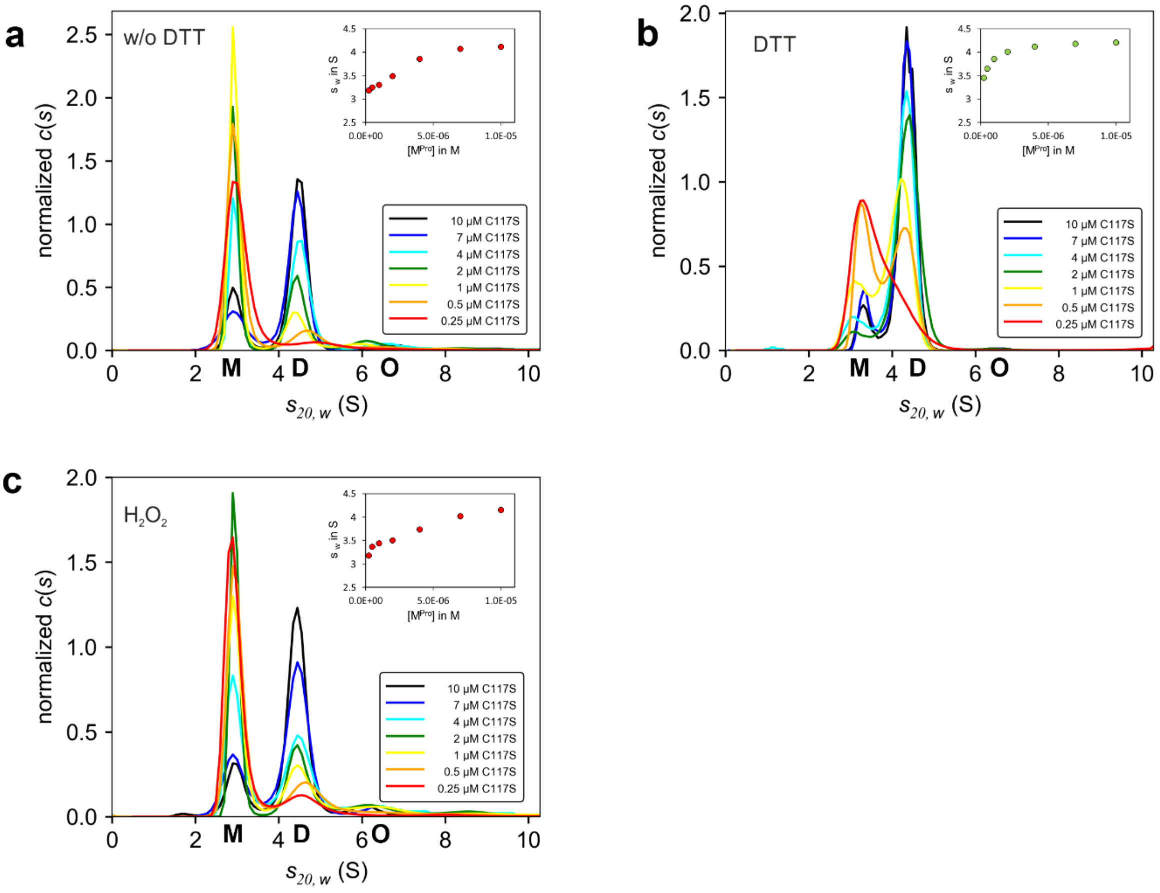

a
w/o DTT
normalized c(s)
s20, w (S)
M
D
O
10 µM C117S
7 µM C117S
4 µM C117S
2 µM C117S
1 µM C117S
0.5 µM C117S
0.25 µM C117S
[MPro] in M
b
DTT
c
H2O2

C145S

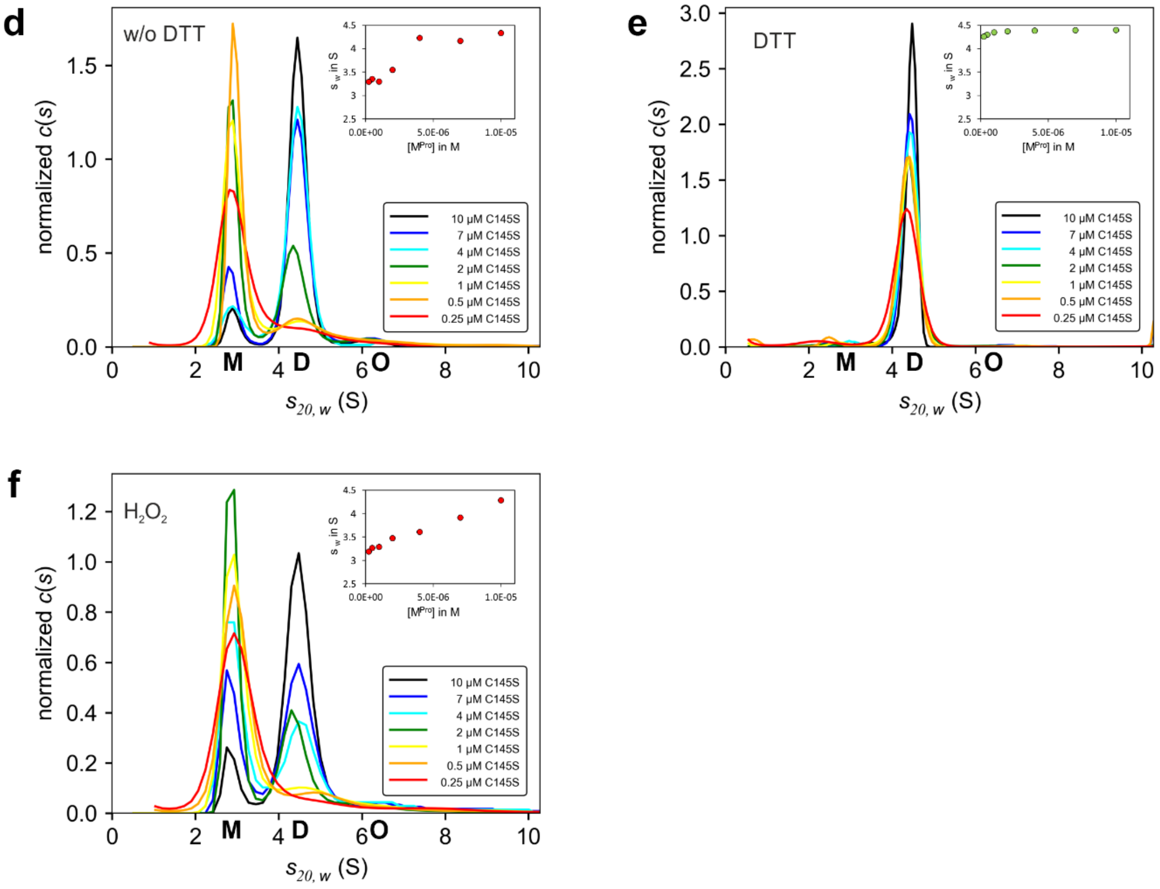

d
w/o DTT
normalized c(s)
s20, w (S)
M
D
O
10 µM C145S
7 µM C145S
4 µM C145S
2 µM C145S
1 µM C145S
0.5 µM C145S
0.25 µM C145S
[MPro] in M
e
DTT
f
H2O2

Supplementary Figure 14. Redox-dependent sedimentation velocity analysis of SARS-CoV-2 M^{pro} variants C117S (a-c) and C145S (d-f). The oligomeric equilibrium was analyzed in a concentration range from 0.25 to 10 µM under either non-reducing (**a/c,** buffer devoid of DTT), reducing (**b/d**, buffer supplemented with 1 mM DTT) or oxidizing (**c/f**, 1 mM H_2O_2) conditions. Insets show s_w binding isotherms, as calculated from the corresponding c(s) distributions. As opposed to the wild-type enzyme and all other variants tested, variant C117S undergoes monomerization also under reducing conditions with an equilibrium constant K_D^{app} in the range of 1 µM. For C145S, we observe a redox-dependent monomer ⇔ dimer equilibrium with an apparent equilibrium constant of K_D^{app} <0.25 µM for the reduced enzyme. Under oxidizing conditions, K_D^{app} is in the lower micromolar range for both mutants. Abbreviations: M, monomer ($s_{20,w}$ = 2.9 S); D, dimer ($s_{20,w}$ = 4.5 S); O, oligomers ($s_{20,w}$ ≈ 6.3 S).

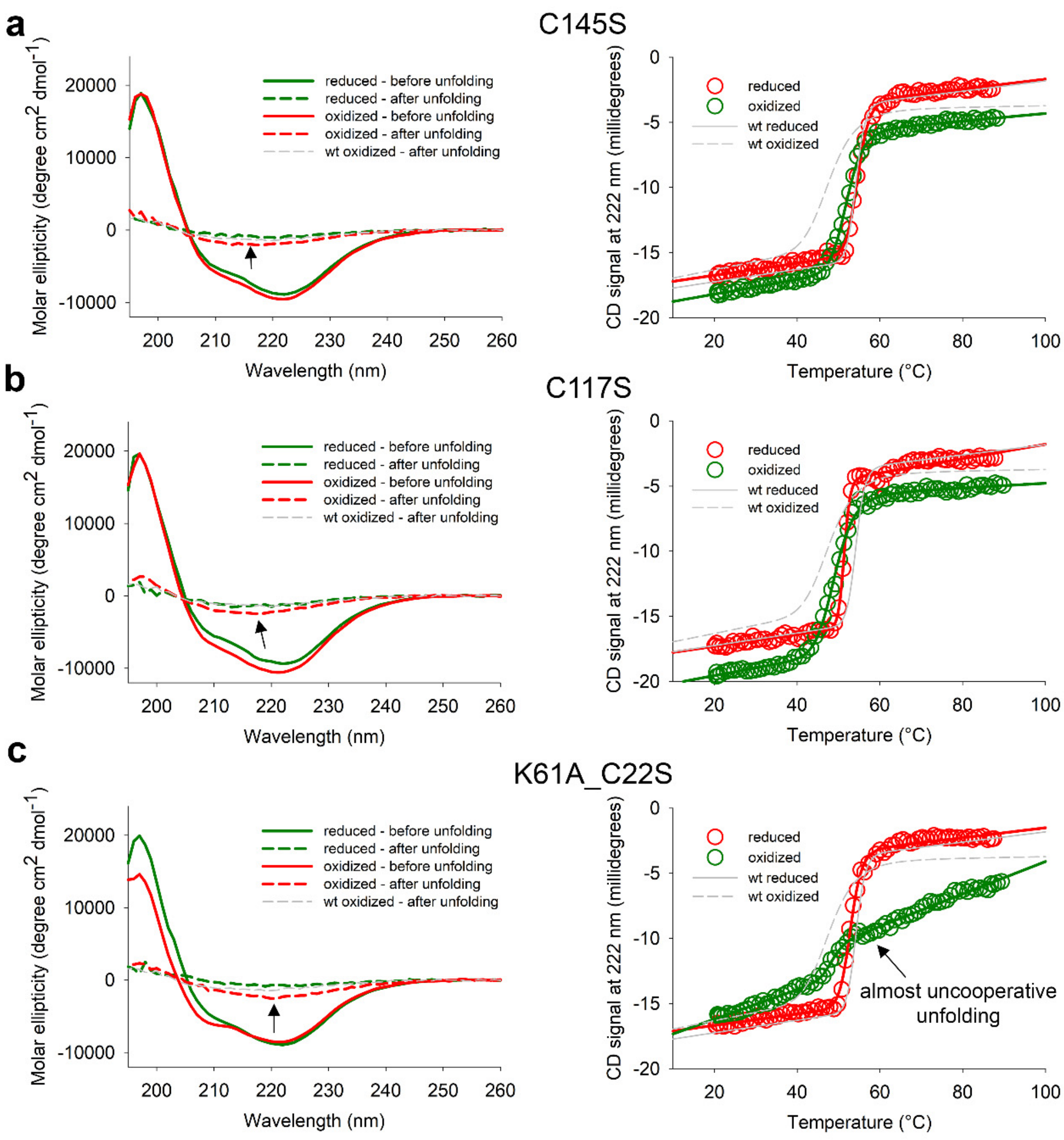

Supplementary Figure 15. Secondary structure and thermal unfolding analysis of M^pro variants C145S (**a**), C117S (**b**) and K61A_C22S (**c**) by far-UV CD spectroscopy under reducing and oxidizing conditions. The structural changes of both C145S and C117S upon oxidation are different compared to the wild-type protein (see Figure 1) and do not include an increase of ß-strand structural elements at the expense of α-helices (Supplementary Table 4). Also, the melting temperatures (Supplementary Table 5) and cooperativity of unfolding are almost identical for both proteins under reducing and oxidizing conditions. Variant K61A_C22S shows the typical structural transition following an oxidation. Note, however, the atypical early onset of unfolding (below 40 °C) and decreased cooperativity (decreased steepness of transition) of unfolding of the oxidized enzyme in case of the K61A_C22S double variant that is implying a loosely associated structure and a high tendency to undergo aggregation. This is further supported by the detection of increased residual ß-sheet-like structures after thermal unfolding indicated by the arrows.

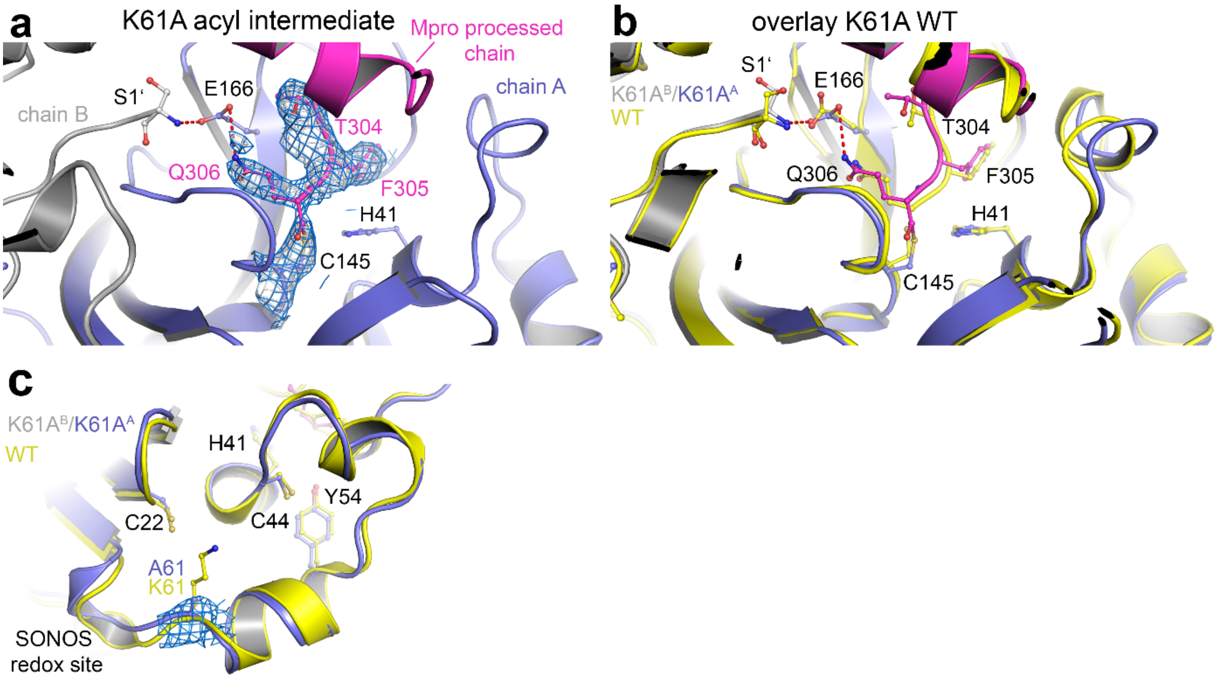

Supplementary Figure 16. X-ray crystallographic structure of the covalent acyl intermediate in M^{pro} variant K61A. (**a**) Snapshot of the acyl intermediate in M^{pro} K61A (this study) formed between a functional dimer in the asymmetric unit and a symmetry-related M^{pro} molecule. The two chains of the dimer are colored individually (chain A, blue; chain B, grey). Residues contributed by chain B are marked with an apostrophe. The C-terminus of the symmetry-related molecule, which visits the active site and forms a covalent linkage with catalytic C145, is highlighted in magenta. The structural model of C145 and the last three C-terminal residues of the symmetry-related molecule are superposed with the corresponding 2mFo-DFc electron density map contoured at 1σ. (**b**) Superposition of the WT structure (yellow) with that of variant K61A (blue/grey, symmetry-related molecule magenta) showing the active sites. Note the structural differences at the active site and at the dimer interface, in particular of C145 and E166. (**c**) Superposition of the wild-type (WT) structure (yellow) with that of variant K61A (blue), showing the allosteric SONOS redox switch sites. The structural model of mutation site A61 in the variant is superposed with the corresponding 2mFo-DFc electron density map.

Dimedone at C145

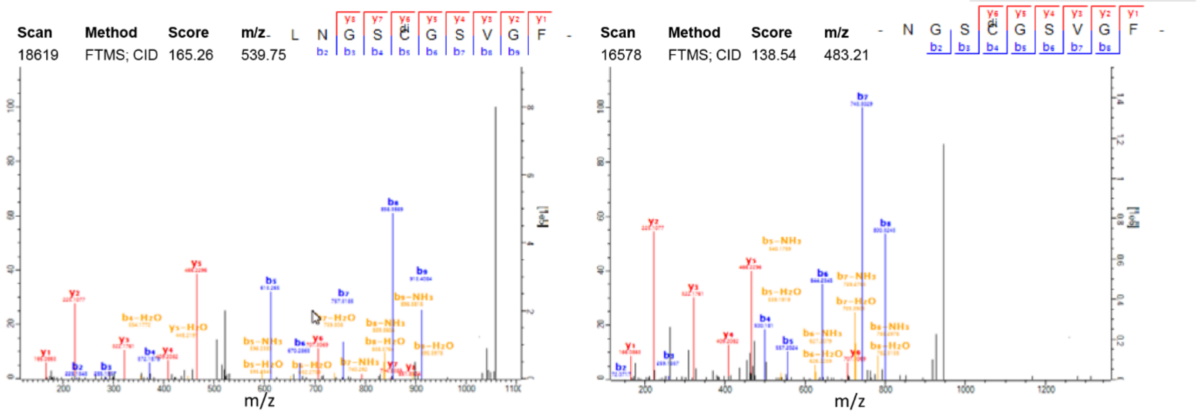

Dimedone at C156

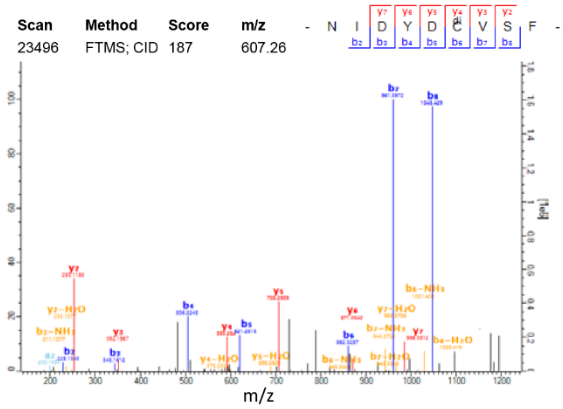

Dimedone at C300

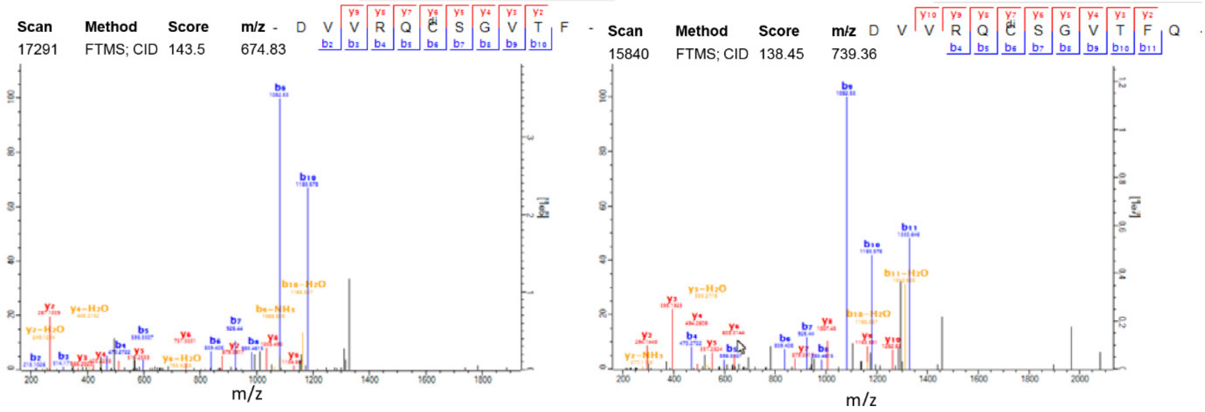

Supplementary Figure 17. Representative mass spectra of dimedone containing peptides of SARS-CoV-2 M^pro. Dimedone (di) was detected as a modification of several peptides using tandem mass spectrometry upon treatment of M^pro with 1 mM H_2O_2 for 10 minutes followed by incubation with 5 mM dimedone for 30 minutes. Recorded spectra suggest the existence of sulfenic acids at residues C145, C156 and C300.

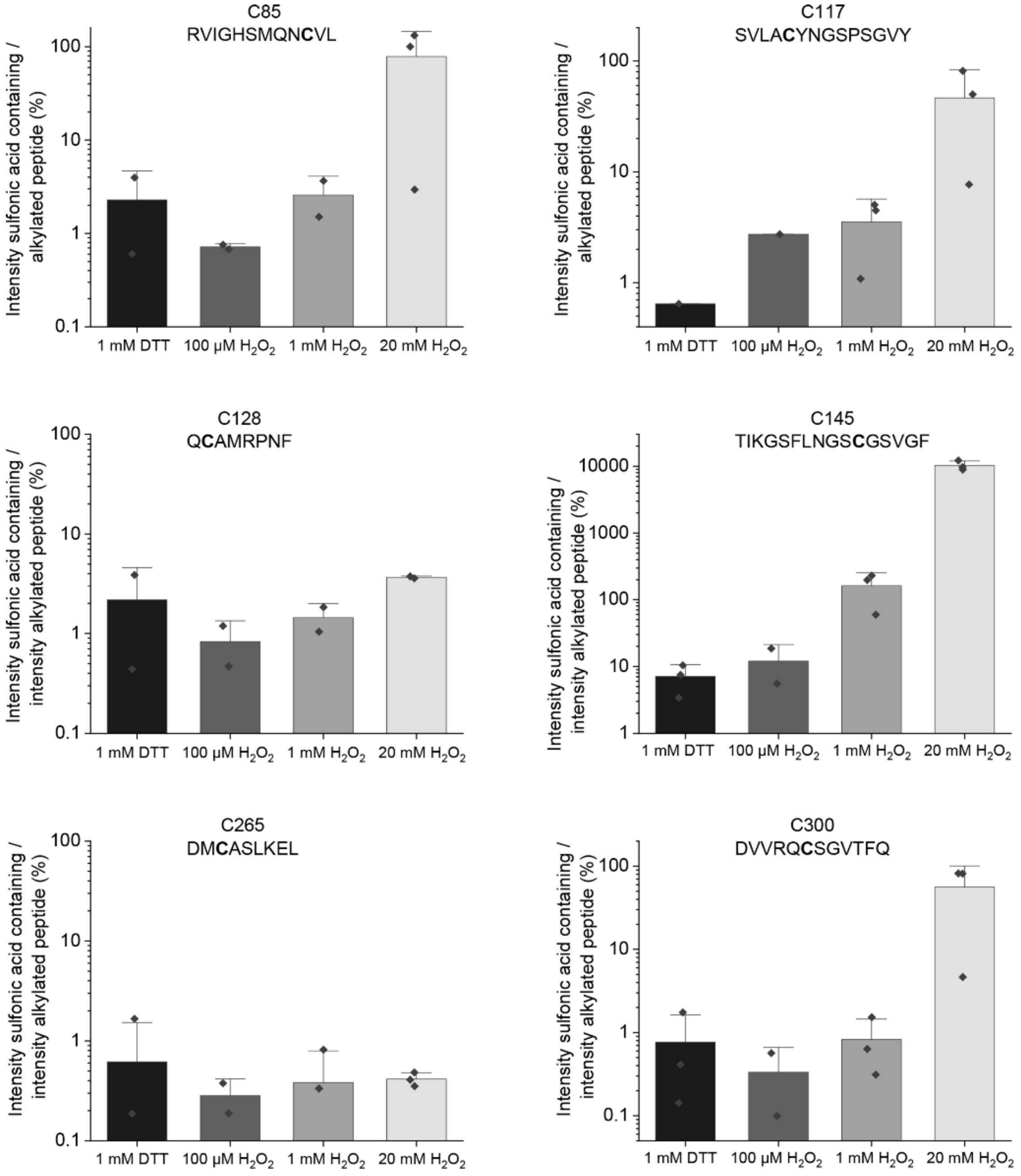

Supplementary Figure 18. Analysis of cysteine sulfonylation of SARS-CoV-2 M^{pro} using mass spectrometry. SARS-CoV-2 M^{pro} was incubated for 2 h on ice with either 1 mM DTT, 100 µM H_2O_2, 1 mM H_2O_2 or for 20 min with 20 mM H_2O_2, respectively. Subsequently M^{pro} was alkylated, digested with chymotrypsin and analysed by mass spectrometry. Ratios of peptide intensities (cysteine sulfonylated peptide variant / variant with alkylated cysteines) are shown (n=3, 100 µM H_2O_2 n=2) for cysteine-containing peptides where intensities could be repetitively found in all analysed conditions. Bars represent mean values and error bars the corresponding standard deviation. Representative spectra of sulfonylated peptides can be found in SI Figure 19.

C16

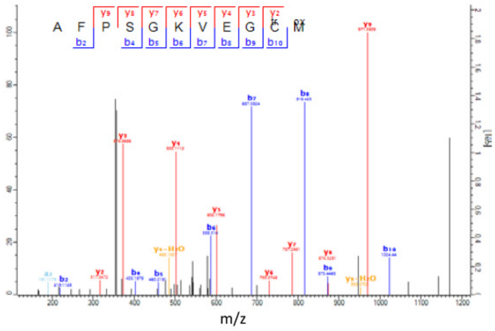

Scan	Method	Score	PEP	Charge	m/z	Mass error [ppm]
6718	FTMS; CID	140.16	3.3 E-18	2	895.25	-1.45

C22

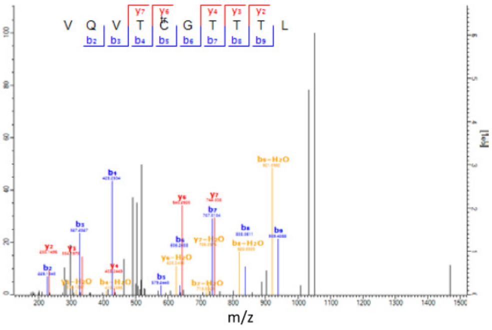

Scan	Method	Score	PEP	Charge	m/z	Mass error [ppm]
12248	FTMS; CID	134.05	3.3 E-12	2	535.76	-0.52

C85

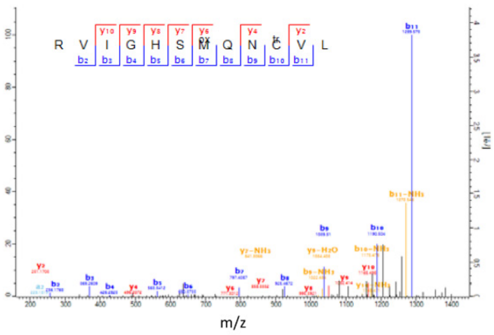

Scan	Method	Score	PEP	Charge	m/z	Mass error [ppm]
6741	FTMS; CID	117.14	7 E-8	2	710.84	-1.45

C117

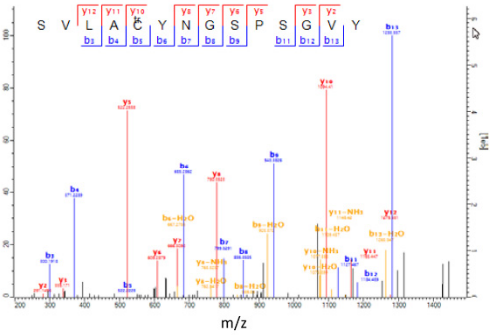

Scan	Method	Score	PEP	Charge	m/z	Mass error [ppm]
15973	FTMS; CID	169.82	6.1 E-47	2	732.82	0.23

C128

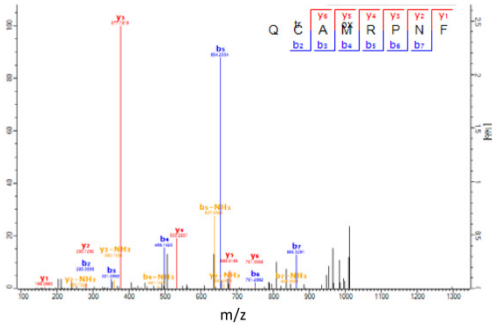

Scan	Method	Score	PEP	Charge	m/z	Mass error [ppm]
6428	FTMS; CID	134.66	2 E-10	2	515.71	2.4 E-5

C145

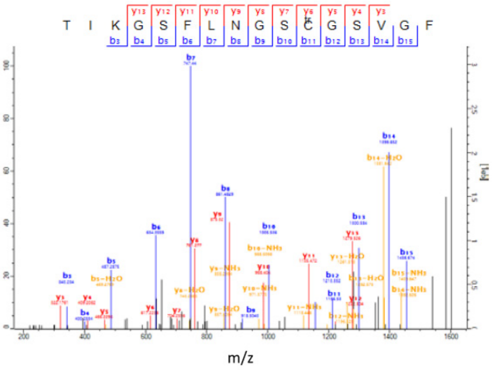

Scan	Method	Score	PEP	Charge	m/z	Mass error [ppm]
15682	FTMS; CID	259.7	2.5 E-230	2	811.38	-0.17

C156

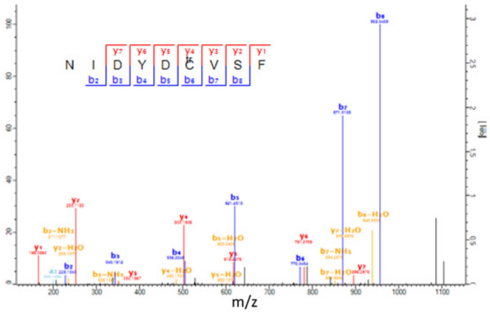

Scan	Method	Score	PEP	Charge	m/z	Mass error [ppm]
17987	FTMS; CID	171.75	4.4 E-36	2	562.22	-0.38

C265

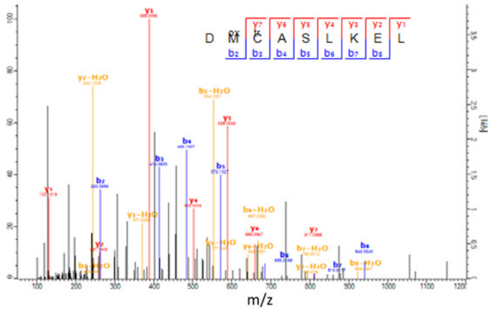

Scan	Method	Score	PEP	Charge	m/z	Mass error [ppm]
11117	FTMS; HCD	150.36	1.4 E-13	2	537.23	0.0003

C300

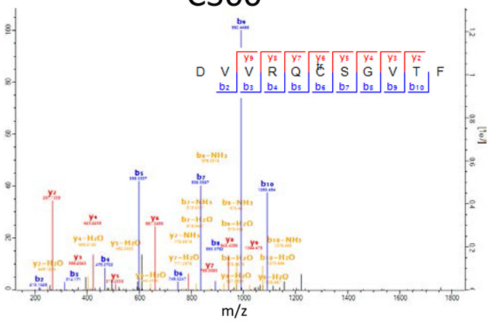

Scan	Method	Score	PEP	Charge	m/z	Mass error [ppm]
13206	FTMS; CID	285	8.2 E-263	2	629.79	-0.53

Supplementary Figure 19. Analysis of SARS-CoV-2 M^{pro} sulfonylation using mass spectrometry. Sulfonylation at cysteine residues (marked with tr for trioxidation) could be identified under different treatment conditions. Exemplary spectra are shown including information on peptide identification. Sulfonylated C22 could only be found at non-physiological conditons (M^{pro} treated with 20 mM H_2O_2). We found no evidence for sulfonylation at C38, C44 and C160 under all conditions tested.

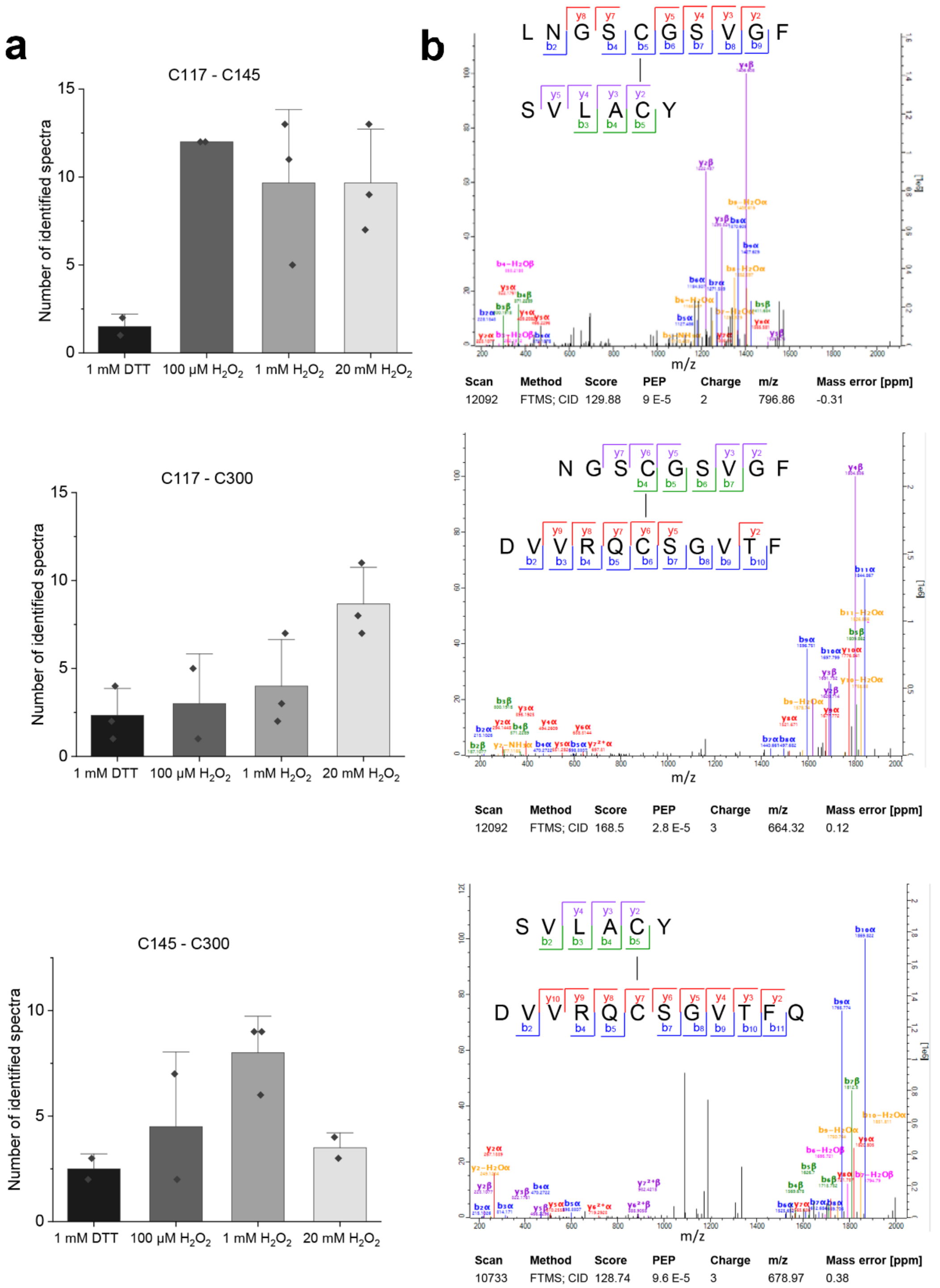

Supplementary Figure 20. Representative mass spectra of disulfide-containing peptides of SARS-CoV-2 M^{pro}. SARS-CoV-2 M^{pro} was incubated for 2 h on ice with either 1 mM DTT, 100 µM H_2O_2, 1 mM H_2O_2 or for 20 min with 20 mM H_2O_2, respectively. Subsequently, M^{pro} was alkylated, digested with chymotrypsin and analysed with mass spectrometry. Crosslinks were reported if found repetitively in 100 µM H_2O_2-treated samples. The number of found crosslinked peptides is shown in **a** and exemplary spectra from 100 µM H_2O_2 treated M^{pro} samples in **b**. Bars represent mean values and error bars the corresponding standard deviation.

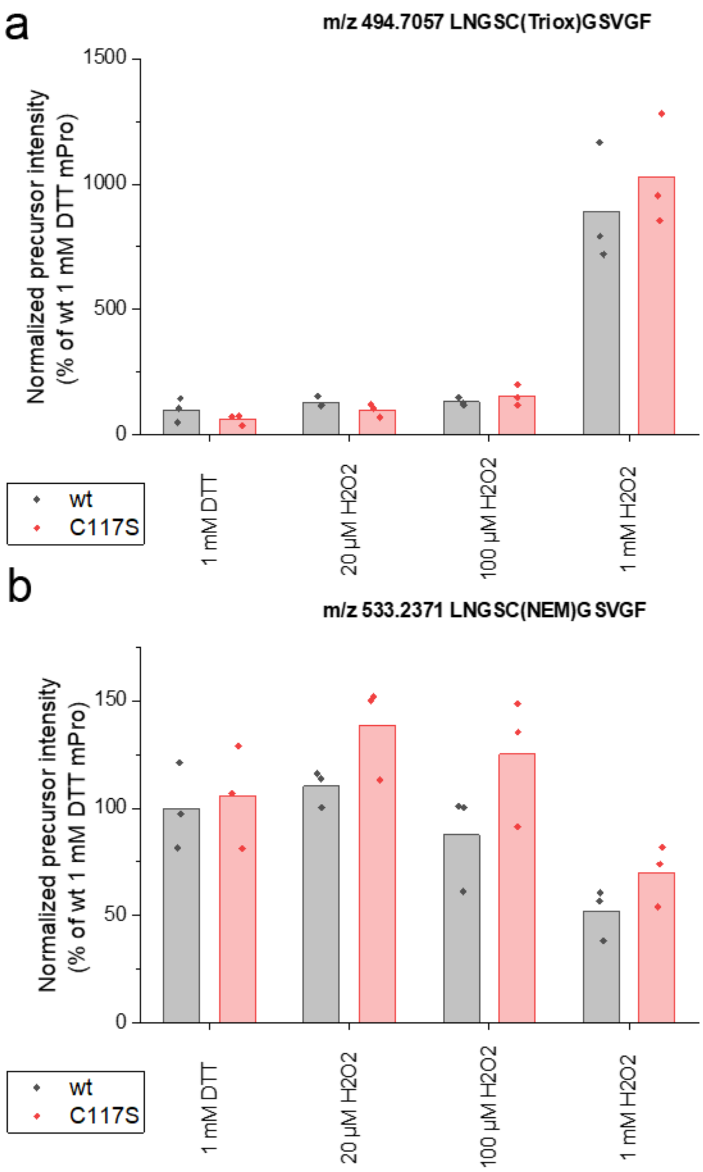

Supplementary Figure 21. Mass spectrometric analysis of M^{pro} variant C117S (in red) versus the wild-type enzyme (in grey) for reducing and different oxidizing conditions showing the extent of tri-oxidized C145 (catalytic cysteine) in panel (**a**) and modification with N-ethylmaleimide which might represent the reduced peptide variant in panel (**b**) (n = 3 for all samples). Peptide quantification is based on extracted ion chromatograms using m/z 494.7057 (sulfonic acid variant) and m/z 533.2371(N-ethylmaleimide variant) of peptide LNGSCGSVGF. Peptides TQDHVDIL (m/z 470.7404) and SAQTGIAVL (m/z 430.2478) have been used for normalization to account for total M^{pro} amount and normalized intensities scaled on the intensity of the reduced sample (set to 100%). Note the increased fractions of overoxidized C145 and alkylated C145 for variant C117S, in which the C117-C145 disulfide-dithiol switch cannot be formed.

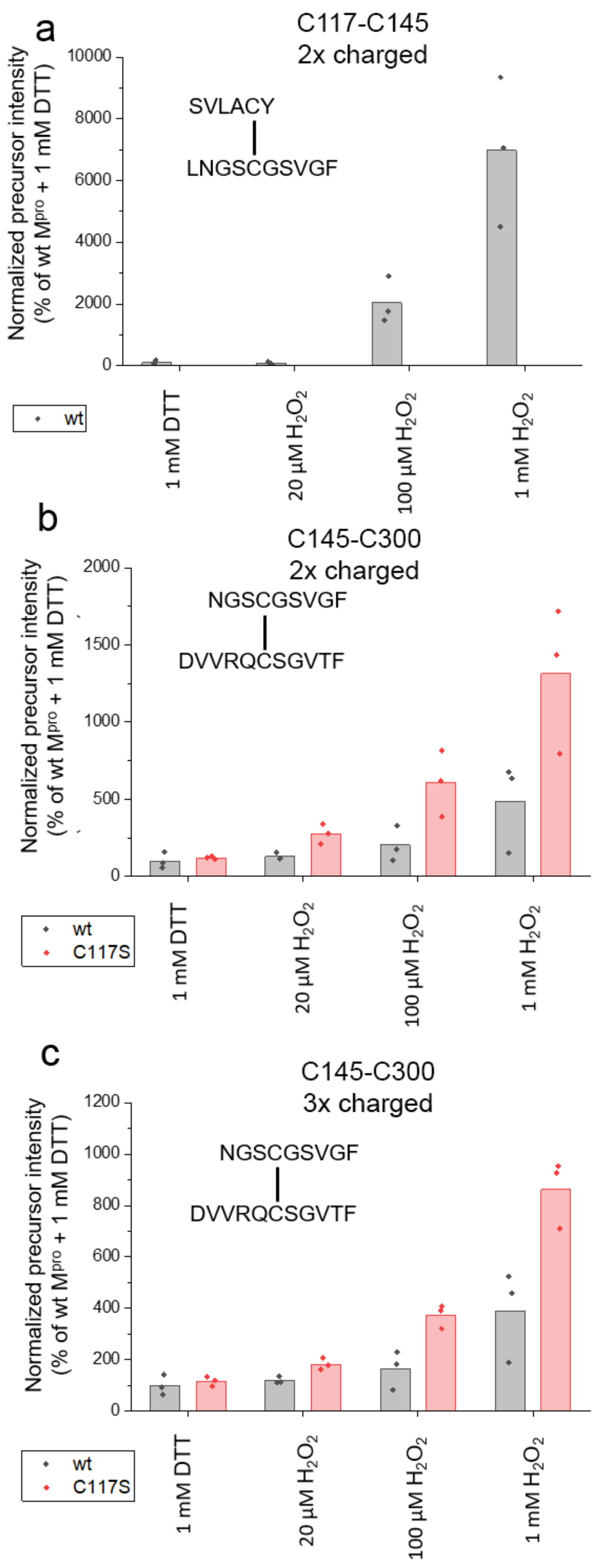

Supplementary Figure 22. Mass spectrometric analysis of disulfide-containing peptides of SARS-CoV-2 M^{pro} wild-type versus variant C117S (n = 3 / group). SARS-CoV-2 M^{pro} was incubated for 2 h on ice with either 1 mM DTT, 20 µM H_2O_2, 100 µM H_2O_2 or 1 mM H_2O_2,

respectively. Subsequently, M^{pro} was alkylated, digested with chymotrypsin and analyzed with mass spectrometry. Extracted ion chromatograms were used to quantify relevant disulfide peptide pairs. Peptides TQDHVDIL (m/z 470.7404) and SAQTGIAVL (m/z 430.2478) have been used for normalization to account for total M^{pro} amount and normalized intensities scaled on the intensity of the reduced sample (set to 100%). Relative intensities are shown for C117-C145 (a, wild-type only), C145-C300 2x charged (panel b, wild-type and variant C117S) and C145-C300 3x charged (panel c, wild-type and variant C117S).

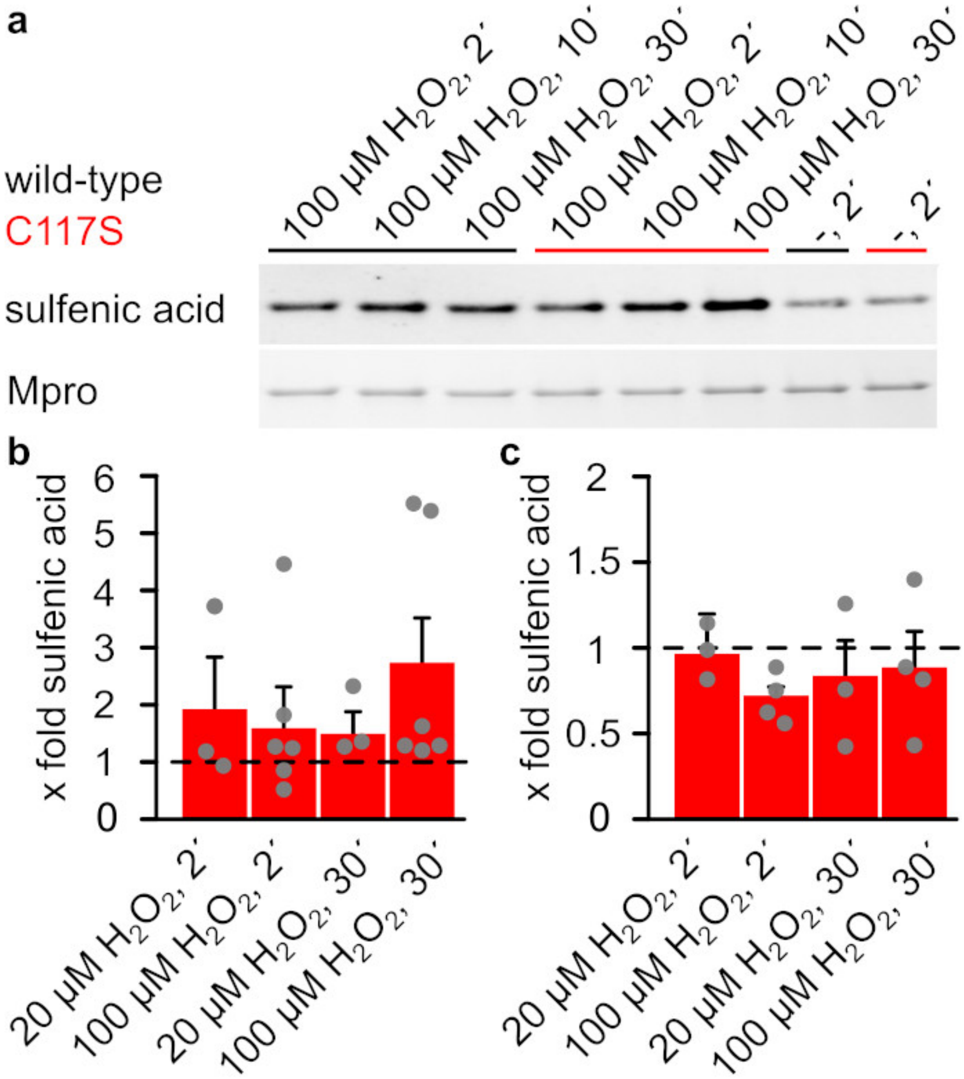

Supplementary Fig. 23. Analysis of sulfenylation of M^pro wild-type versus variant C117S. M^pro wild-type (black) and M^pro variant C117S (red) were either reduced (1 mM DTT) or oxidized (freeze/thawing) and afterwards incubated with H_2O_2 at the indicated concentrations and time periods in the presence of 5 mM dimedone (marker for sulfenylated cysteines). After western blotting, proteins were visualized using the stain free tool and the formed sulfenic acids by anti-dimedone antibodies (**a**, reduced proteins). The sulfenic acid staining was applied to M^pro staining and the relative ratio between M^pro C117S and M^pro wild-type is shown for initially reduced (**b**) and oxidized proteins (**c**). Note that the extent of sulfenylation is increased for variant C117S under reducing conditions indicating a higher sensitivity to oxidation (panel b). In contrast, when using oxidizing conditions prior to sulfenylation analysis, an inverted ratio is observed possible reflecting an overoxidation of variant C117S relative to the wild-type enzyme.

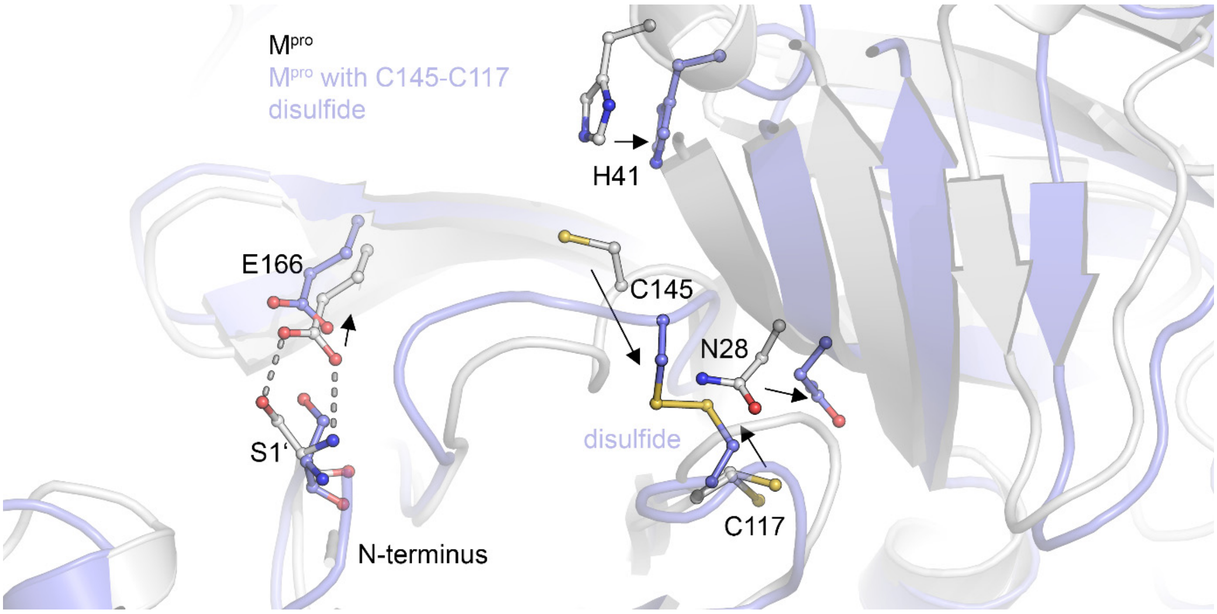

Supplementary Figure 24. Structural changes of SARS-CoV-2 M^{pro} associated with disulfide formation between residues C145 and C117. Structural superposition of M^{pro} in the reduced state (grey, pdb code 7KPH) and with a C145-C117 disulfide (blue, MD simulation) showing the two cysteines C145/C117, neighboring residues H41, N28 and dimer interface residues E166 and S1' contributed from the second subunit. Note that disulfide formation goes along with a structural change of the loop bearing C145 that entails a displacement of neighboring sheets including N28. The structural transition is propagated to the dimer interface and leads to a loss of the H-bonding interaction between E166 and S1' as a critical determinant of dimer stability.

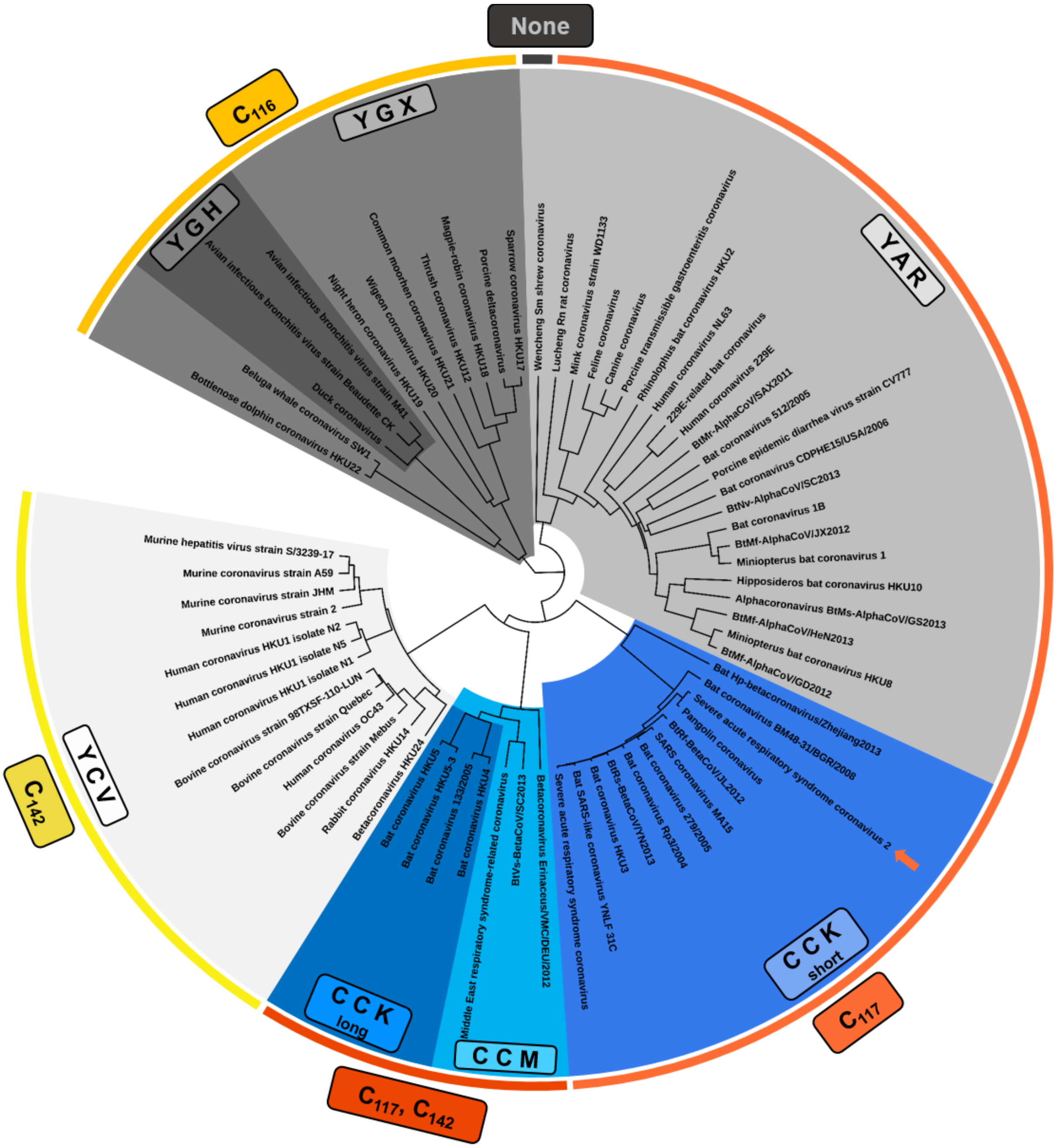

Supplementary Fig.25. Tree-like representation of the dataset used for sequence conservation analyses in this work. Groups with similar SONOS triads are indicated by grey and blue hues, the respective amino acids at position 22, 44 and 61 are indicated (YCV, CCK, CCM, YAR, YGX, YGH). Groups with similar putative disulfide-forming partners for cysteine 145 are indicated by yellow and orange hues (C142, C117 or C142, C117, C116). The red arrow indicates the main protease from SARS-CoV-2.

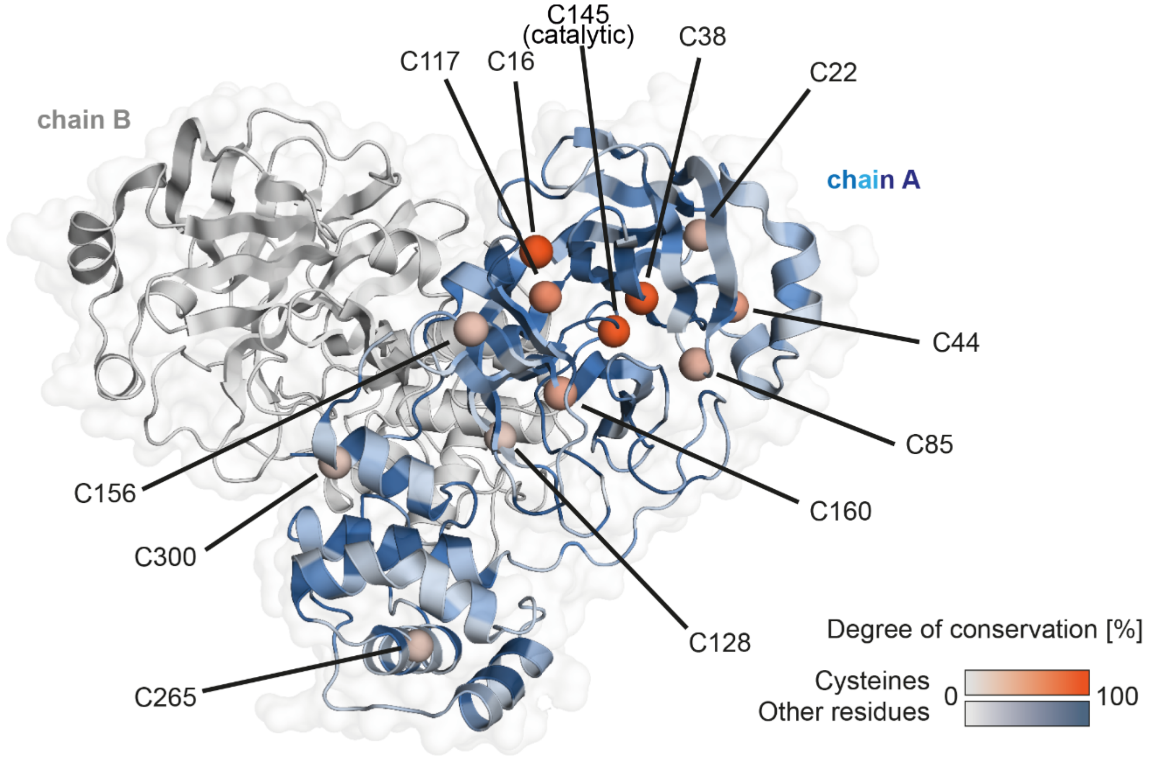

Supplementary Figure 26. Mapping of sequence conservation among coronavirus main proteases onto the structure of the SARS-CoV-2 M^{Pro}. Overall conservation is shown in hues of blue, the conservation of the twelve cysteines in hues of red.

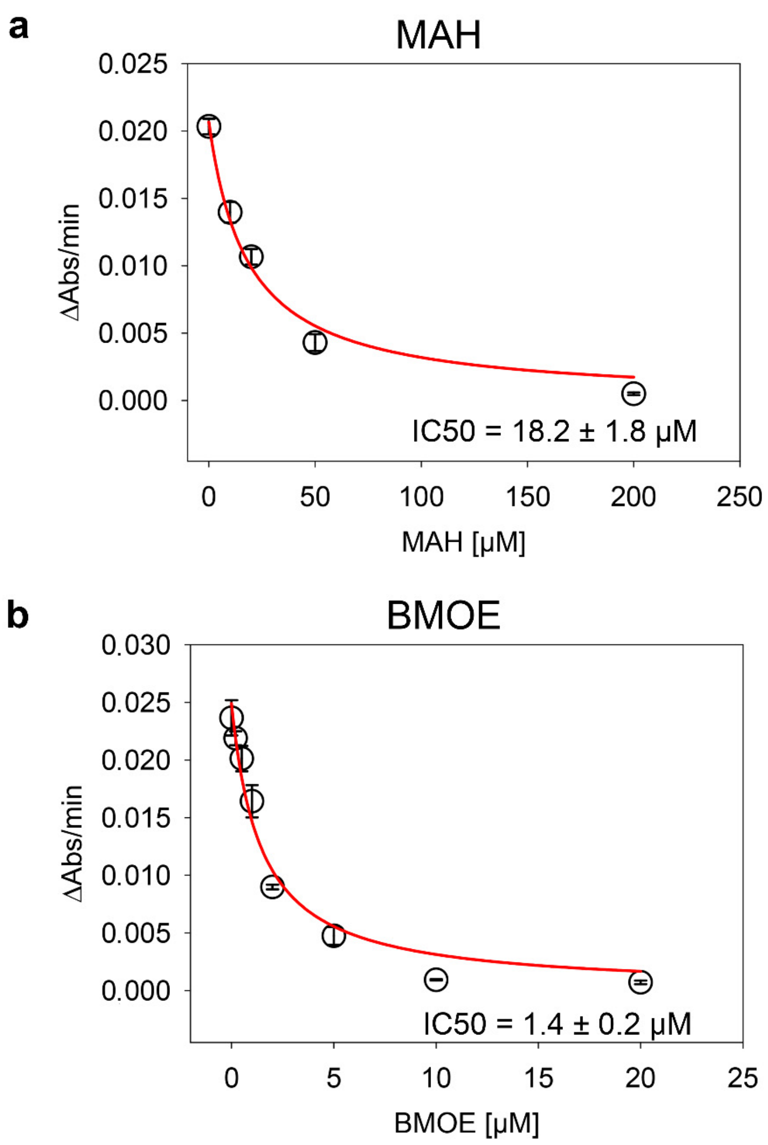

Supplementary Figure 27. Estimation of IC50 values for inhibition of SARS-CoV-2 M^{pro} by bifunctional crosslinkers MAH (**a**) and BMOE (**b**). Enzymatic activity of M^{pro} was measured after pre-incubation with varying concentrations of MAH or BMOE for 30 min using a chromophoric peptide substrate as detailed in the methods section. Data were fitted with eq 2, the fit is shown as a red line. All measurements were carried out in triplicate and are shown as mean ± s.d.

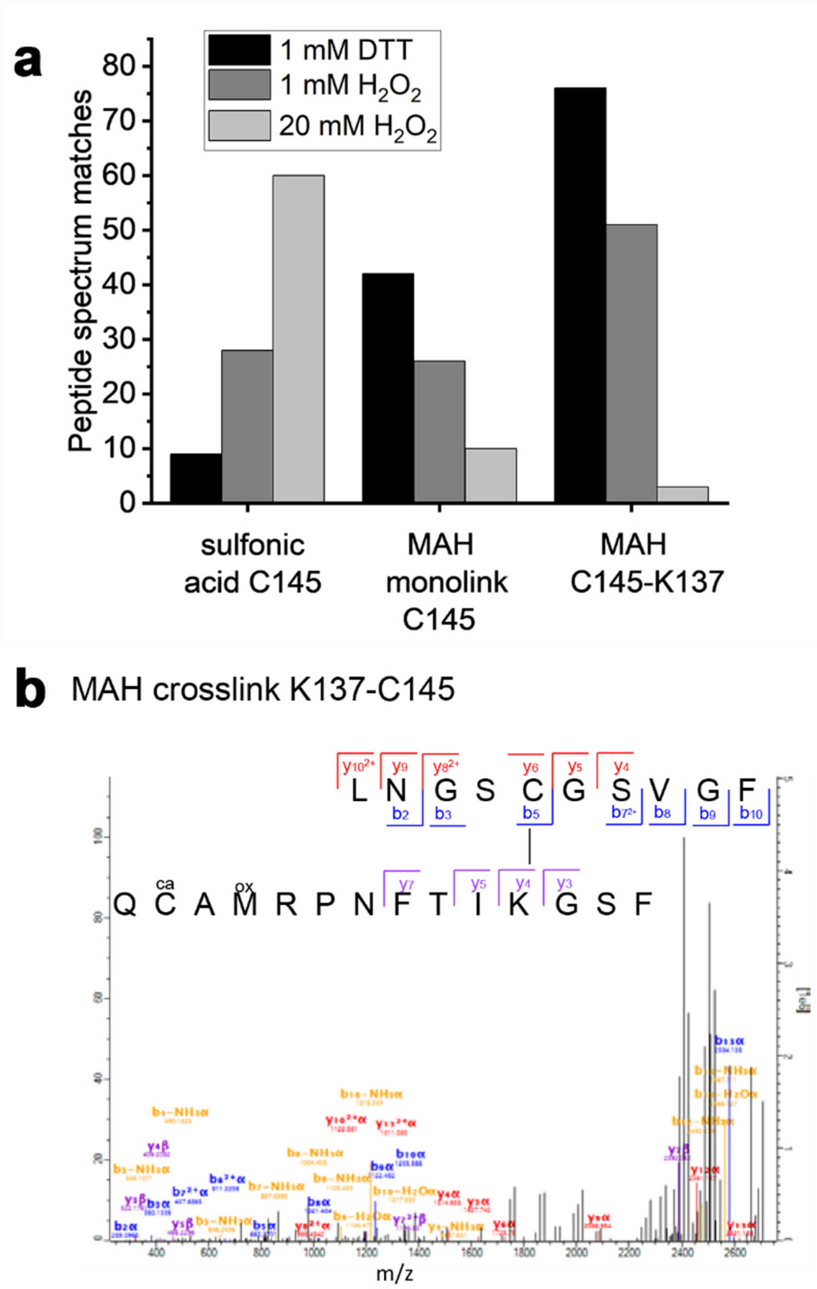

Scan	Method	Score	PEP	Charge	m/z	Mass error [ppm]
16050	FTMS; CID	188.71	0.00135	3	917.0771	-0.028

Supplementary Figure 28. Mass spectrometric analysis of covalent modifications of M^{pro} after reaction with heterobifunctional crosslinker maleimidoacetic acid N-hydroxysuccinimide ester (MAH) under different redox conditions. A reaction scheme for MAH crosslinking is shown in Figure 7c of the main manuscript. (**a**) Residues C145 (thiol) and K137 (amine) were identified as main reaction sites (exemplary spectrum in (**b**), ca: carbamidomethylation, ox: oxidation). With increasing H_2O_2 concentrations, the fraction of sulfonylated C145 becomes dominant based on the counts of peptide spectrum matches.

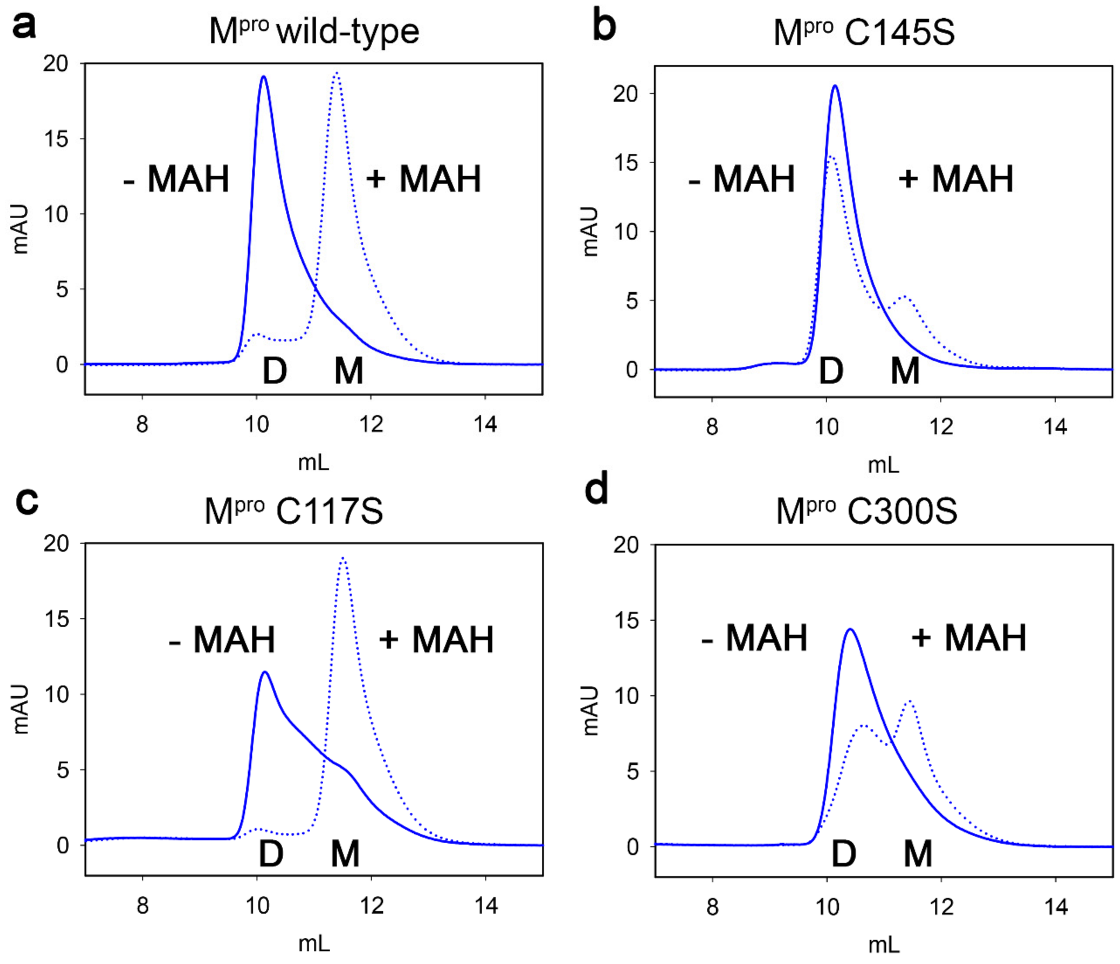

Supplementary Figure 29. Impact of the heterobifunctional crosslinker MAH on the oligomeric equilibrium of M^{pro} wild-type and selected variants as revealed by gel filtration experiments. Abbreviations: D, dimer; M, monomer. The gel filtration profiles are shown as blue solid lines for the proteins in the absence of MAH and as blue dotted lines after reaction with MAH as detailed in the Methods section. (**a**) M^{pro} wild-type: reaction with MAH entails an almost complete monomerization. (**b**) M^{pro} variant C145S: Monomerization upon reaction with MAH is strongly abolished. (**c**) M^{pro} variant C117S: reaction with MAH leads to an almost complete monomerization akin to M^{pro} wild-type. (d) M^{pro} variant C300S: reaction with MAH leads to a marked shift from the dimer to the monomer albeit not as quantitative as observed for wild-type M^{pro}.

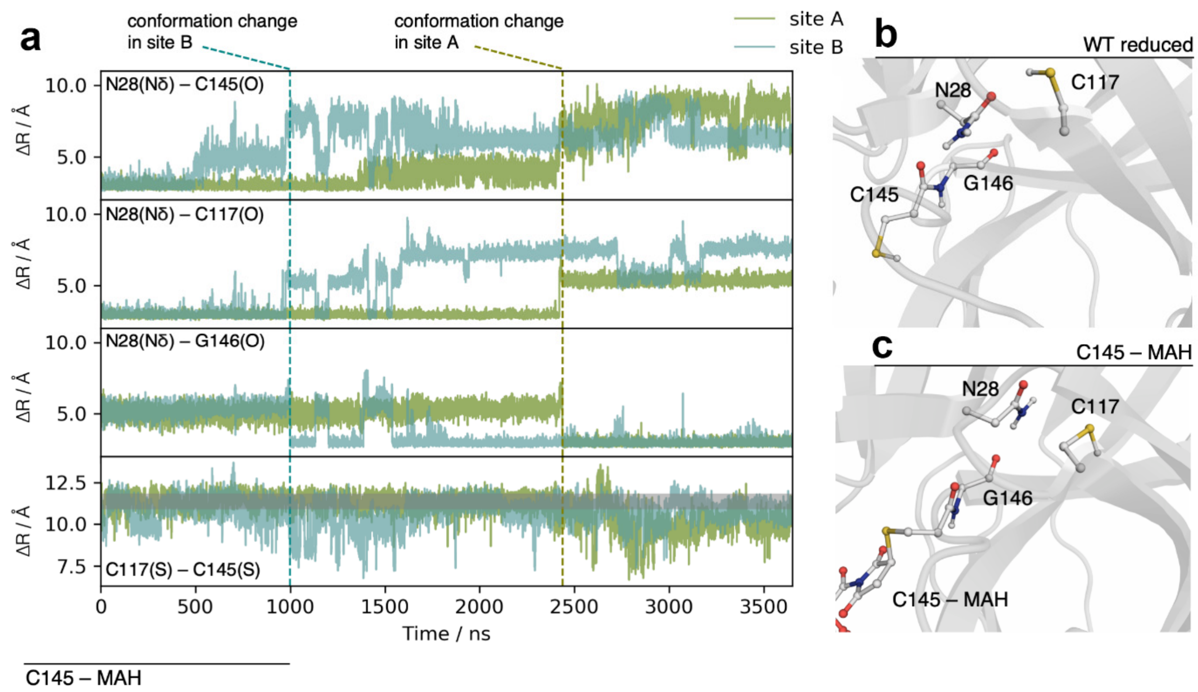

Supplementary Figure 30. MD simulations of M^{pro} after reaction with MAH. (**a**) Selected distances along the 3.65 µs MD trajectory of M^{pro} with MAH covalently bound to C145. Long simulation times are required but a similar effect to the one observed in the disulfide C145 – C117 system takes place. After about 1 µs for the active site of chain B and 2.5 µs in chain A (approximately marked by the vertical dotted lines), the productive interactions of N28 with both cysteines are disrupted and N28 flips interacting with the backbone carbonyl of G146. After the conformation changes, C117 and C145 come in closer proximity in several events. The horizontal grey line depicts the average C117(S)-C145(S) distance in the reduced WT. (**b,c**) Snapshots taken from MD trajectories illustrating the interactions of N28. The N28 interaction with C117 and C145 is again illustrated for the reduced WT (**b**). In the case of the covalent bound C145 – MAH structure, a similar flip of N28 is observed as in the disulfide case (**c**).

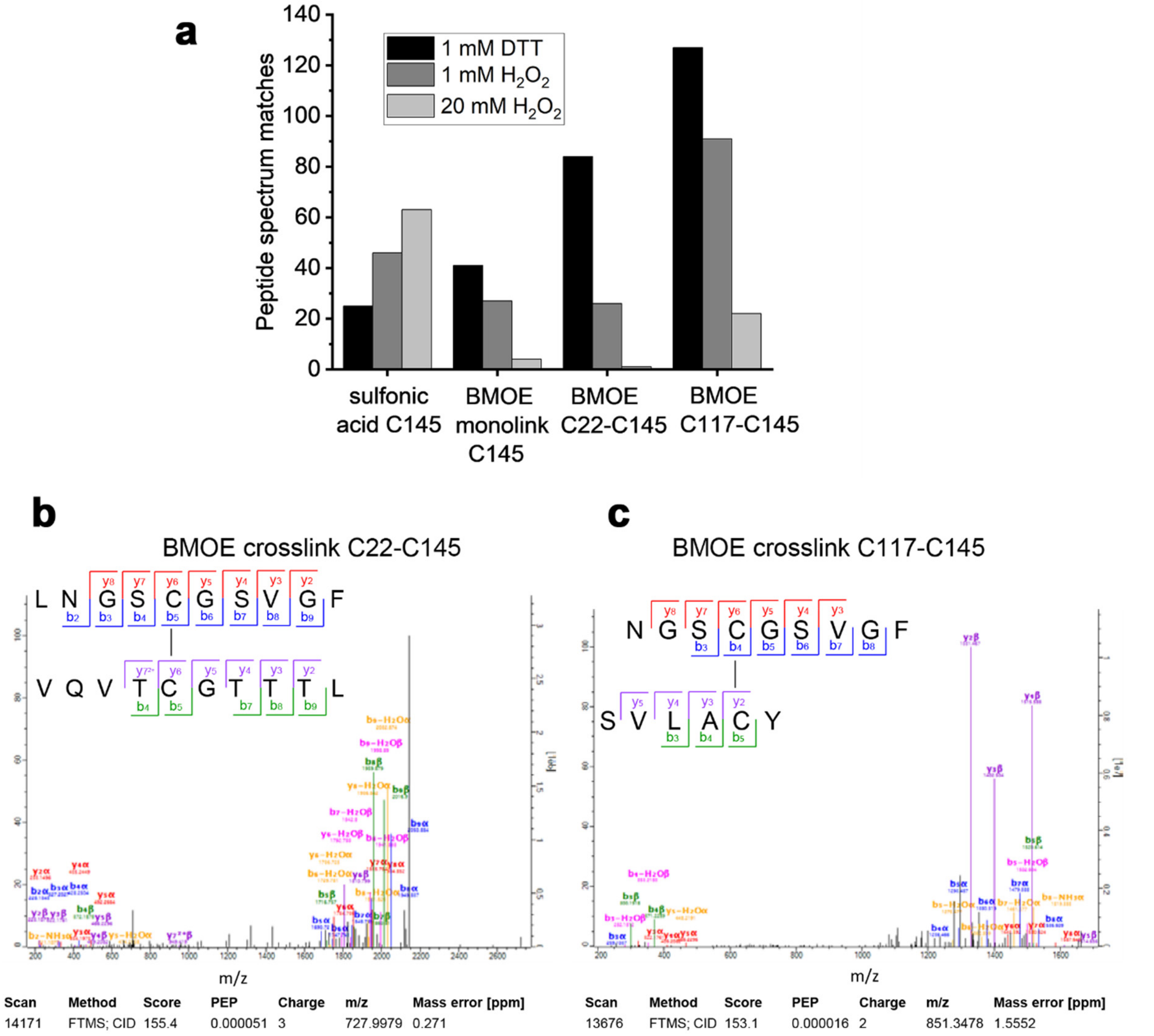

Supplementary Figure 31. Mass spectrometric analysis of covalent modifications of M^{pro} after reaction with homobifunctional crosslinker bismaleimidoethane (BMOE) under different redox conditions. (**a**) Residues C145 and C117 were identified as main crosslink sites (exemplary spectrum in **c**) as well as – to a lesser extent – C145 and C22 (exemplary spectrum in (**b**)). With increasing H_2O_2 concentrations, the fraction of sulfonylated C145 becomes dominant based on the counts of peptide spectrum matches.

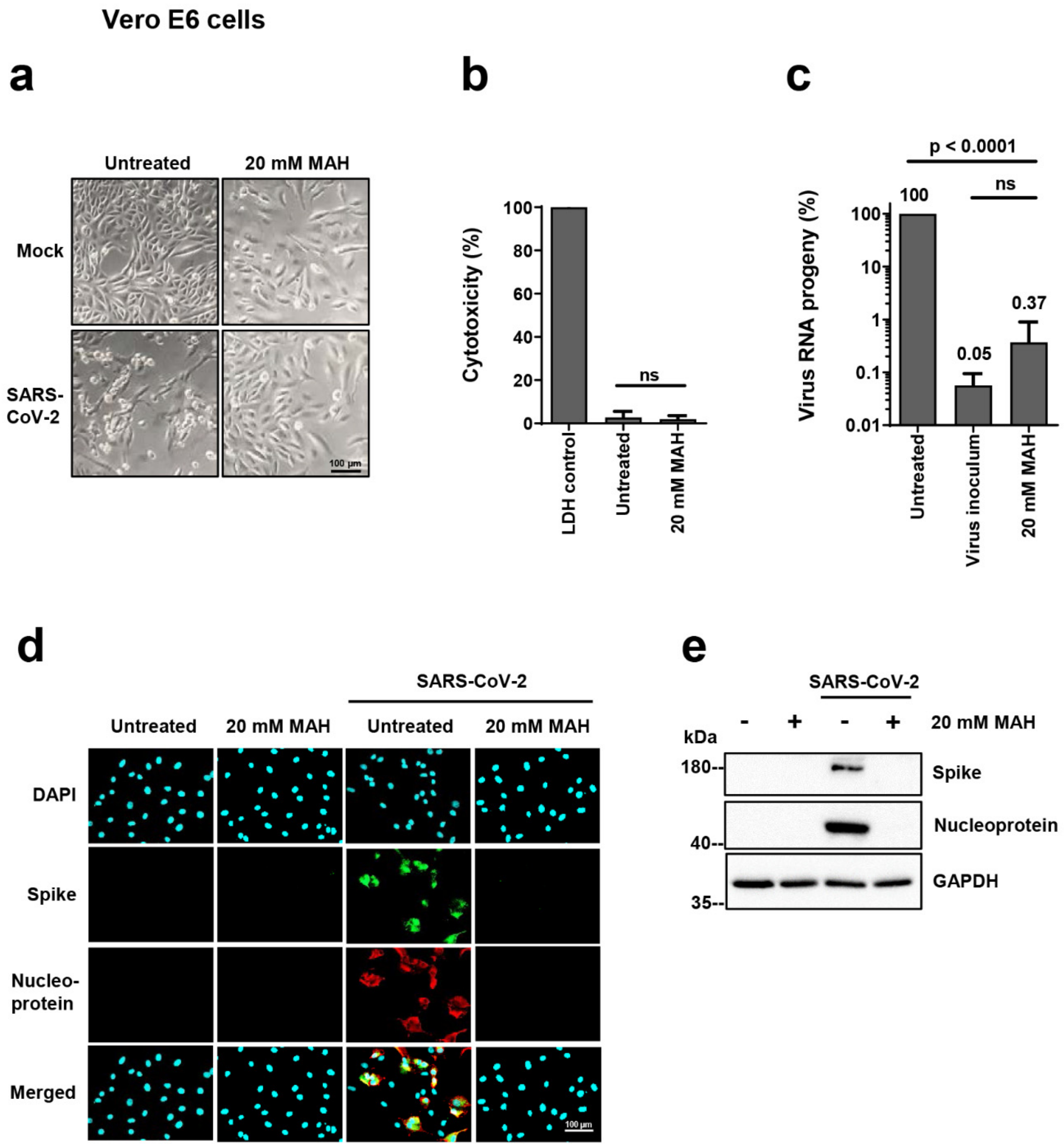

Supplementary Figure 32. MAH inhibits SARS-CoV-2 propagation and the synthesis of viral proteins with mild cytotoxicity. (**a**) Reduced cytopathic effect (CPE) upon treatment with MAH. Vero E6 cells were treated with 20 mM MAH or the PBS control for 1 h before infection, and then throughout the time of infection (48 h). Cell morphology was assessed by bright field microscopy. Note that the CPE was clearly visible in virus-infected cells but to a far lesser extent upon treatment with MAH. (**b**) Cytotoxicity by MAH. Vero E6 cells were treated with 20 mM MAH for 48 h. The release of lactate dehydrogenase (LDH) to the supernatant was quantified by bioluminescence as a read-out for cytotoxicity. The percentages reflect the proportion of LDH released to the media, compared to the overall amount of LDH in the cells (LDH control) (mean with SD, n=3). A systematic analysis of MAH cytotoxicity is provided in **SI Fig. 35**. (**b**) Diminished virus RNA progeny by MAH. Vero E6 cells were treated and infected as described in A. RNA was isolated from the cell culture supernatant, and SARS-CoV-2 RNA was quantified by qRT-PCR. The amount of RNA found upon infection without drug treatment was defined as 100%, and the other RNA quantities were normalized accordingly. RNA was also isolated from the virus inoculum used to infect the cells. Note that MAH reduced SARS-CoV-2 RNA progeny more than 200-fold when compared to the untreated control (mean with SD, n = 3). **(d)** Representative images showing the reduction of viral protein synthesis by MAH.

Vero E6 cells were treated and infected with SARS-CoV-2 as in A. Cell nuclei were stained with DAPI, and the SARS-CoV-2 Spike and Nucleoprotein were detected by immunofluorescence microscopy. € Reduced viral protein synthesis in the presence of MAH. Upon drug treatment and/or infection of Vero E6 cells, the viral Spike and Nucleoprotein as well as GAPDH (loading control) were detected by immunoblot analysis.

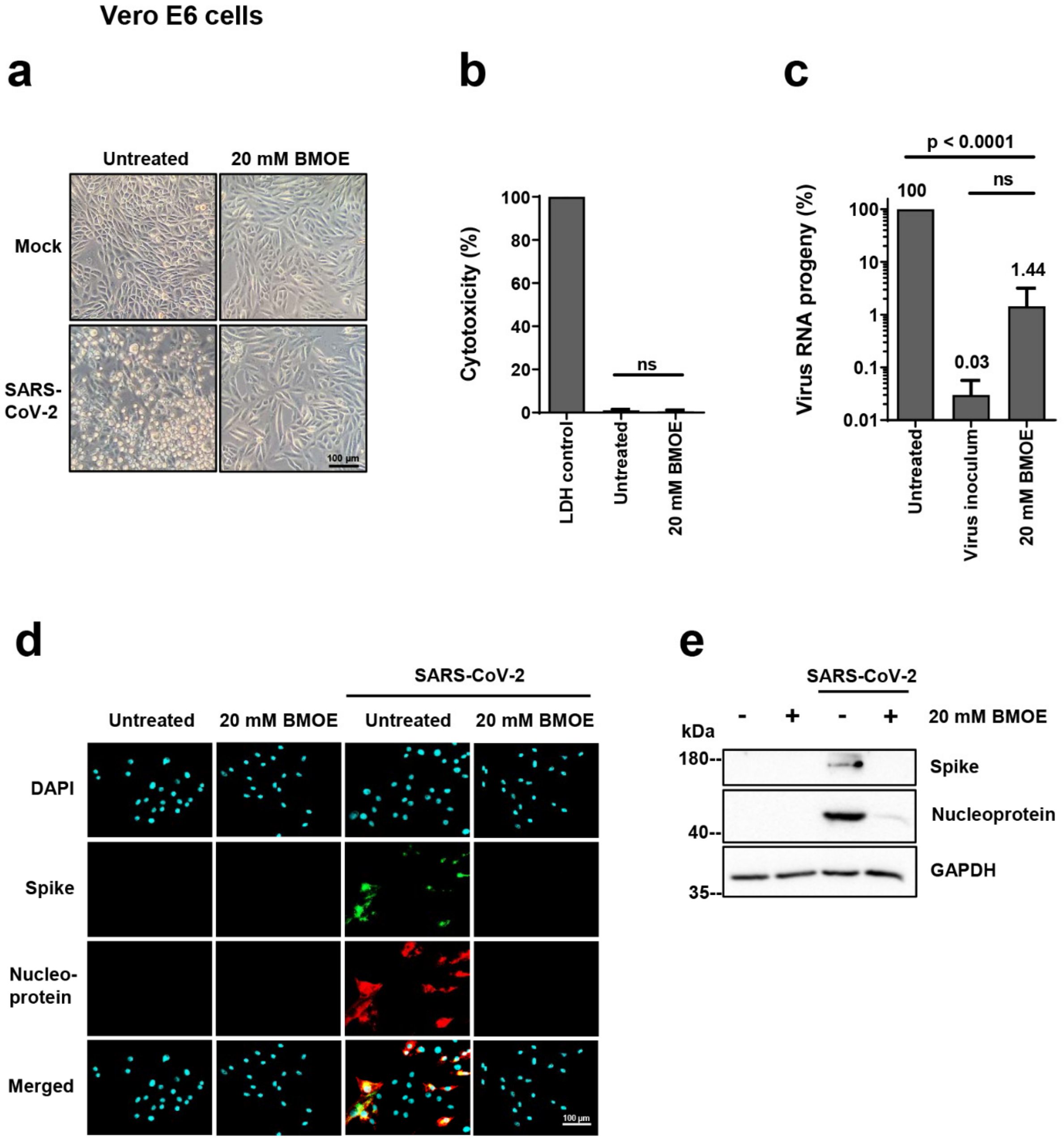

Supplementary Figure 33. BMOE inhibits SARS-CoV-2 propagation and the synthesis of viral proteins with mild cytotoxicity. (**a**) Reduced cytopathic effect (CPE) upon treatment with BMOE. Vero E6 cells were treated with 20 mM BMOE or the PBS control for 1 h before infection, and then throughout the time of infection (48 h). Cell morphology was assessed by bright field microscopy. Note that the CPE was clearly visible in virus-infected cells but to a far lesser extent upon treatment with BMOE. (**b**) Cytotoxicity by BMOE. Vero E6 cells were treated with 20 mM BMOE for 48 h. The release of lactate dehydrogenase (LDH) to the supernatant was quantified by bioluminescence as a read-out for cytotoxicity. The percentages reflect the proportion of LDH released to the media, compared to the overall amount of LDH in the cells (LDH control) (mean with SD, n=3). A systematic analysis of BMOE cytotoxicity is provided in **SI Fig. 35.** (**c**) Diminished virus RNA progeny by BMOE. Vero E6 cells were treated and infected as described in A. RNA was isolated from the cell culture supernatant, and SARS-CoV-2 RNA was quantified by qRT-PCR. The amount of RNA found upon infection without drug treatment was defined as 100%, and the other RNA quantities were normalized

accordingly. RNA was also isolated from the virus inoculum used to infect the cells. Note that BMOE reduced SARS-CoV-2 RNA progeny more than 60-fold when compared to the untreated control (mean with SD, n = 3). **(d)** Representative images showing the reduction of viral protein synthesis by BMOE. Vero E6 cells were treated and infected with SARS-CoV-2 as in A. Cell nuclei were stained with DAPI, and the SARS-CoV-2 Spike and Nucleoprotein were detected by immunofluorescence microscopy. € Reduced viral protein synthesis in the presence of MAH. Upon drug treatment and/or infection of Vero E6 cells, the viral Spike and Nucleoprotein as well as GAPDH (loading control) were detected by immunoblot analysis.

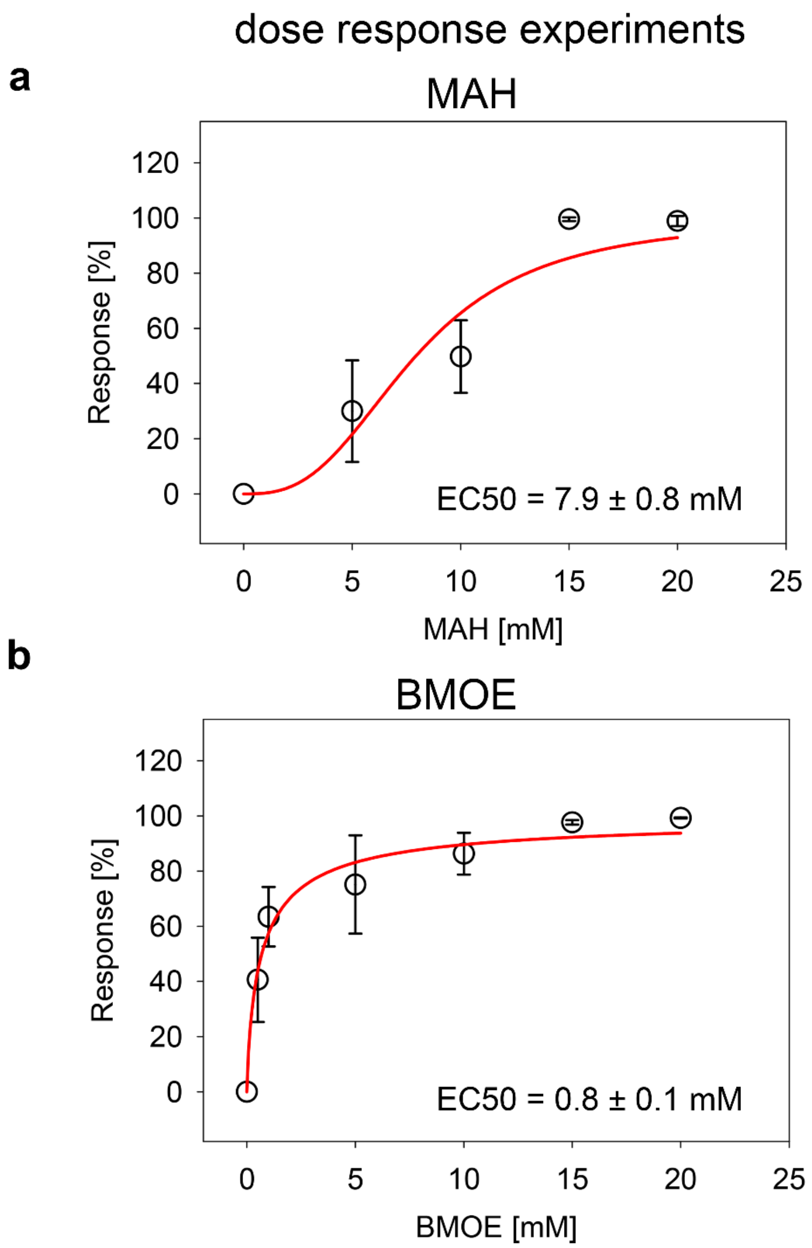

Supplementary Figure 34. Dose response experiments for inhibition of SARS-CoV-2 by bifunctional crosslinkers MAH (**a**) and BMOE (**b**). Virus RNA progeny in Vero 6 cells was analyzed at different concentrations of the crosslinkers in individual experiments using cell line SARS-CoV-2 infection models as outlined in the methods section. Data were fitted with eq 3, the fit is shown as a red line. All experiments were carried out in triplicate and the detected dose response at each concentration is shown as mean ± s.d.

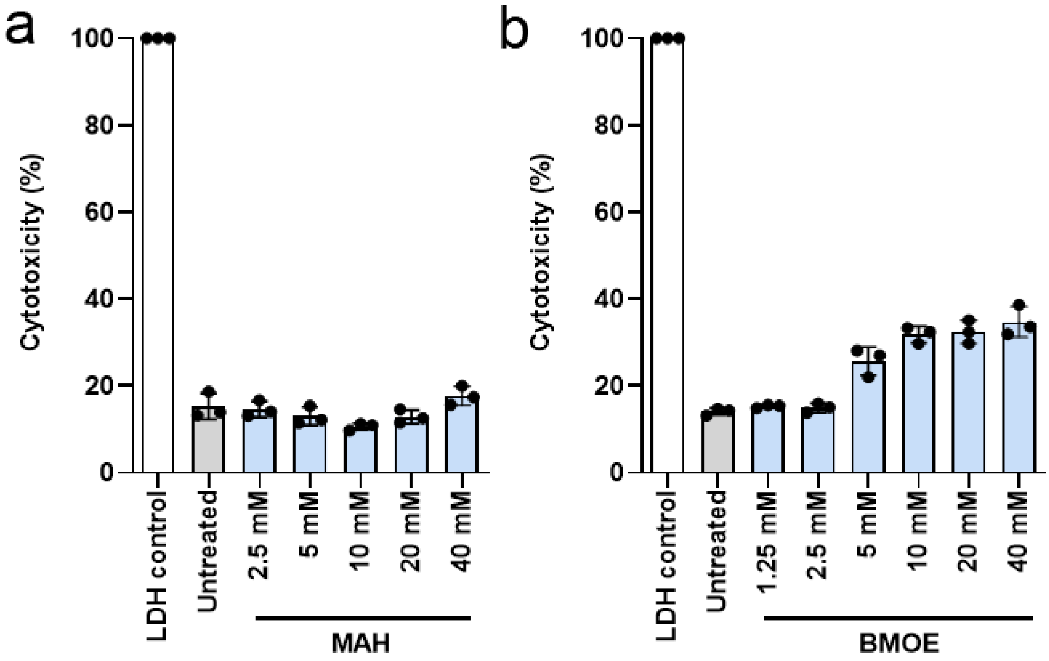

Supplementary Figure 35. Analysis of the cytotoxic effect of crosslinkers MAH (panel a) and BMOE (panel b) on Vero E6 cells using the LDH assay (see Materials and Methods section). Note the mild cytotoxic effect of MAH up to concentrations of 40 mM and the slightly stronger cytotoxicity of BMOE in the same concentration regime. Since the inhibition profiles of both crosslinkers in the dose response experiments are in a regime of little cytotoxicity (see Supplementary Figure 34), we conclude that the inhibitory effect results from impairing SARS-CoV-2 propagation.

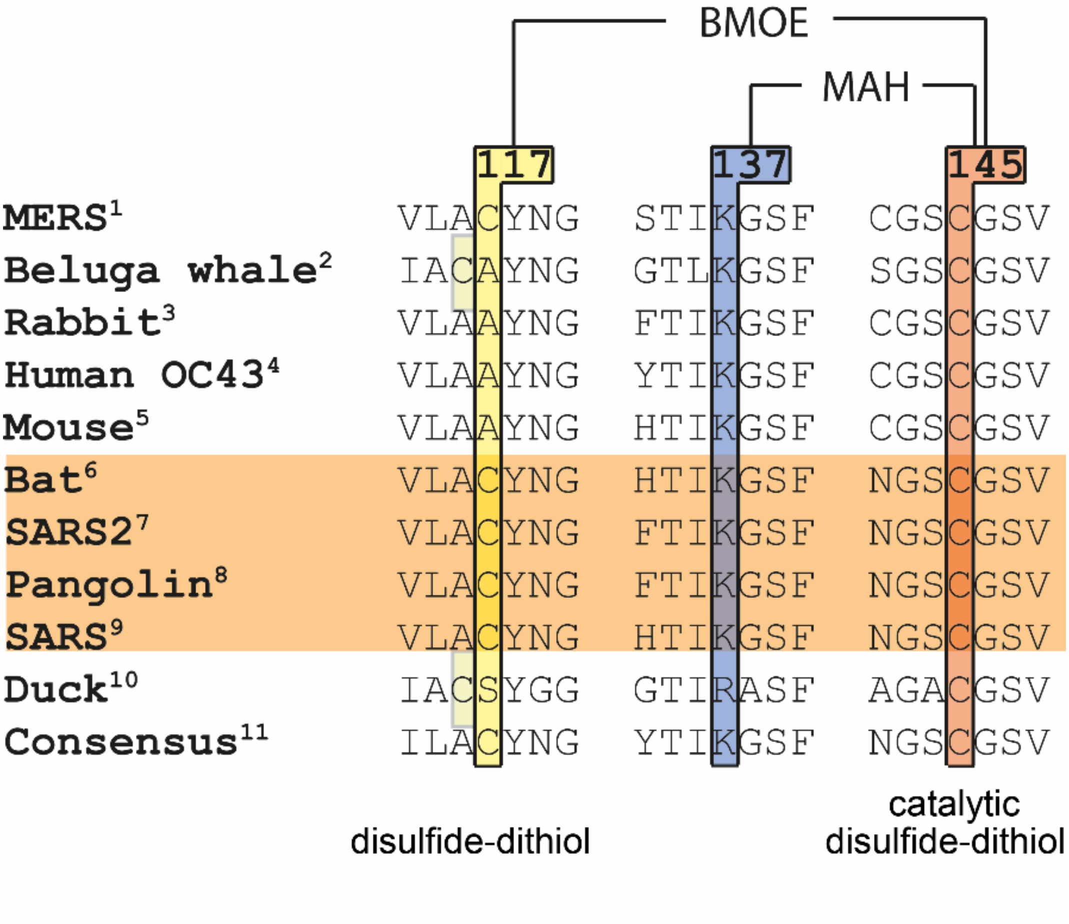

Supplementary Figure 36. Sequence conservation of residues of Coronavirus main proteases targeted by bifunctional crosslinkers MAH and BMOE. UniProtKB ID of polyprotein 1ab: 1 K9N7C7, 2 B2BW31, 3 H9AA60, 4 P0C6X6, 5 P0C6X9, 6 E0XIZ2, 7 P0DTD1, 8 A0A6G6A2G5, 9P0DTD1, 10 A0A0F6WGL5, 11 From 67 sequences. The functions of the individual residues for enzymatic activity and redox regulation are indicated.

Supplementary Table 1. X-ray data collection and refinement statistics.

	C44S<br>Single crystal	K61A<br>Single crystal	Y54F<br>Single crystal
**Data collection**			
Space group	C2	C2	C2
Cell dimensions			
a, *b*, *c* (Å)	122.89, 81.84, 63.56	122.48, 81.29, 63.70	113.66, 53.49, 44.59
α, β, γ (°)	90.00, 90.04, 90.00	90.00, 90.22, 90.00	90.00, 102.03, 90.00
Resolution (Å)	67.84 – 2.12 (2.35 – 2.12)*	46.45 – 2.49 (2.69 – 2.49)	55.63– 1.63 (1.76 – 1.63)
R_{sym} or R_{merge}	0.128 (1.675)	0.171 (1.734)	0.139 (2.695)
$I / \sigma I$	7.6 (1.4)	6.7 (1.2)	12.8 (1.4)
Ellipsoidal completeness (%)	91.9 (61.7)	84.1 (32.4)	92.2 (51.7)
Redundancy	7.1 (7.4)	7.2 (6.7)	16.8 (16.2)
**Refinement**			
Resolution (Å)	46.39 – 2.12	46.451 – 2.49	55.63 – 1.63
No. reflections	22608	16753	23931
R_{work} / R_{free}	26.01/29.64	25.8/33.6	17.4/21.4
No. atoms			
Protein	4753	4726	2531
Water	73	36	237
B-factors			
Protein	73.4	100.1	23.3
Water	54.2	54.6	32.9
R.m.s. deviations			
Bond lengths (Å)	0.003	0.001	0.004
Bond angles (°)	0.532	0.394	0.753
Diffraction limits (Å) and corresponding principal axes of the ellipsoid fitted to the diffraction cut-off surface as direction cosines in the orthogonal basis (standard PDB convention), and in terms of reciprocal unit-cell vectors:			
#1	2.845 (0.925, 0.000, -0.389) 0.622 a* - 0.208 c*	2.763 (0.974, 0.000, -0.225) 0.993 a* - 0.121 c*	2.058 (0.932, 0.000, 0.270) a* + 0.026 c*
#2	2.345 (0.000, 1.000, 0.000) b*	2.558 (0.000, 1.000, 0.000) b*	1.67 (0.000, 1.000, 0.000) b*
#3	2.065 (0.380, 0.000, 0.925) 0.622 a* + 0.783 c*	2.390 (0.225, 0.000, 0.974) 0.406 a* + 0.914 c*	1.586 (-0.270, 0.000, 0.963) -0.567 a* + 0.823 c*

*Values in parentheses are for highest-resolution shell.

Supplementary Table 2. Redox-dependent distribution of M^{pro} oligomeric species for cysteine variants as analyzed by gel filtration experiments in color-coded representation [1].

Quaternary structure	M^{pro} Cysteine Variants	Untreated	Reduced	Oxidized 1h	Oxidized 5h	Re-Reduced
**Oligomer**	WT	0.4%	0.4%	0.7%	0.9%	0.3%
	C16S	0.4%	0.4%	0.9%	9.0%	0.4%
	C22S	0.5%	0.5%	0.9%	6.1%	0.5%
	C38S	0.4%	0.4%	0.8%	5.5%	0.4%
	C44S	0.3%	0.4%	0.5%	5.3%	0.2%
	C85S	0.4%	0.4%	0.5%	4.7%	0.3%
	C117S	0.1%	0.2%	0.1%	1.8%	0.5%
	C128S	0.4%	0.2%	1.1%	6.9%	0.6%
	C145S	0.9%	0.8%	3.4%	4.1%	2.0%
	C156S	1.7%	0.9%	1.8%	4.8%	1.2%
	C160S	0.6%	0.5%	1.5%	11.2%	0.4%
	C265S	0.2%	0.1%	0.5%	3.4%	0.0%
	C300S	0.5%	0.6%	0.5%	3.2%	0.4%
**Dimer**	WT	83.6%	83.5%	71.7%	60.0%	84.5%
	C16S	63.4%	63.3%	49.6%	33.4%	61.8%
	C22S	83.0%	82.3%	73.3%	54.1%	82.6%
	C38S	76.7%	76.4%	66.7%	45.1%	76.1%
	C44S	79.5%	79.0%	62.0%	39.6%	78.9%
	C85S	76.3%	76.1%	62.1%	44.0%	75.1%
	C117S	36.5%	36.5%	46.5%	54.1%	42.3%
	C128S	72.1%	72.6%	52.0%	35.4%	71.3%
	C145S	93.5%	91.4%	89.6%	88.0%	89.5%
	C156S	81.6%	82.3%	72.3%	54.5%	81.7%
	C160S	79.4%	79.5%	68.7%	52.8%	78.6%
	C265S	78.2%	77.7%	64.6%	46.1%	77.7%
	C300S	70.6%	70.2%	54.0%	27.3%	69.1%
**Monomer**	WT	15.9%	16.1%	27.6%	39.1%	15.2%
	C16S	36.3%	36.3%	49.5%	57.7%	37.8%
	C22S	16.5%	17.2%	25.8%	39.8%	16.9%
	C38S	22.9%	23.2%	32.5%	49.4%	23.5%
	C44S	20.3%	20.6%	37.6%	55.1%	20.9%
	C85S	23.3%	23.5%	37.4%	51.4%	24.6%
	C117S	63.3%	63.3%	53.4%	44.1%	57.3%
	C128S	27.5%	27.2%	46.9%	57.7%	28.1%
	C145S	5.6%	7.8%	7.0%	7.9%	8.5%
	C156S	16.7%	16.7%	26.0%	40.7%	17.1%
	C160S	20.0%	20.0%	29.7%	36.0%	21.0%
	C265S	21.6%	22.1%	34.9%	50.5%	22.3%
	C300S	28.9%	29.2%	45.4%	69.5%	30.5%

[1] For clarity, relative fractions of each oligomeric state are visualized in color-coded fashion (green: high amount, yellow: medium amount, red: low amount).

Supplementary Table 3. Redox-dependent distribution of M^pro oligomeric species for SONOS variants as analyzed by gel filtration experiments in color-coded representation [1].

Quaternary structure	M^pro SONOS Variants	Untreated	Reduced	Oxidized 1h	Oxidized 5h	Re-Reduced
**Oligomer**	WT	0.44%	0.36%	0.70%	0.90%	0.30%
	C22S	0.48%	0.48%	0.91%	6.08%	0.51%
	C44S	0.25%	0.36%	0.45%	5.34%	0.21%
	C44A	0.39%	0.51%	0.84%	3.52%	0.64%
	C22S_C44S	0.15%	0.25%	2.02%	8.58%	0.32%
	K61A	0.58%	0.52%	1.04%	4.95%	0.72%
	K61A_C22S	0.41%	0.41%	0.60%	6.23%	0.37%
	K61A_C44S	0.21%	0.16%	0.38%	1.01%	0.22%
	K61A_C22S_C44S	0.10%	0.16%	0.27%	8.26%	0.28%
	Y54F	0.26%	0.31%	0.78%	7.62%	0.33%
**Dimer**	WT	83.61%	83.51%	71.74%	60.00%	84.51%
	C22S	83.01%	82.33%	73.30%	54.12%	82.56%
	C44S	79.49%	79.04%	61.95%	39.59%	78.88%
	C44A	83.05%	82.54%	71.46%	56.22%	82.72%
	C22S_C44S	77.02%	77.13%	57.51%	36.93%	76.16%
	K61A	79.47%	79.55%	67.14%	53.10%	79.28%
	K61A_C22S	78.37%	78.19%	65.95%	45.01%	77.57%
	K61A_C44S	77.47%	77.41%	60.70%	38.49%	76.80%
	K61A_C22S_C44S	79.80%	79.04%	57.86%	34.64%	77.57%
	Y54F	78.56%	78.69%	63.30%	45.73%	78.17%
**Monomer**	WT	15.94%	16.13%	27.56%	39.10%	15.18%
	C22S	16.51%	17.19%	25.79%	39.80%	16.93%
	C44S	20.25%	20.60%	37.60%	55.07%	20.91%
	C44A	16.57%	16.95%	27.70%	40.27%	16.65%
	C22S_C44S	22.83%	22.62%	40.47%	54.49%	23.53%
	K61A	19.95%	19.93%	31.83%	41.95%	20.00%
	K61A_C22S	21.23%	21.41%	33.44%	48.77%	22.06%
	K61A_C44S	22.32%	22.43%	38.91%	60.50%	22.98%
	K61A_C22S_C44S	20.10%	20.80%	41.86%	57.09%	22.15%
	Y54F	21.18%	21.00%	35.92%	46.64%	21.50%

[1] For clarity, relative fractions of each oligomeric state are visualized in color-coded fashion (green: high amount, yellow: medium amount, red: low amount).

Supplementary Table 4. Redox-dependent secondary structure contents for M^{pro} wild-type and variants as analyzed by CD spectroscopy in color-coded representation [1].

CD spectra 195-260 nm	α-Helix				
	Reduced	Oxidized	Δ Oxidized vs. Reduced	Δ Reduced rel. to WT	Δ Oxidized rel. to WT
WT	28.80%	27.70%	-1.10%	/	/
C22S	30.40%	28.20%	-2.20%	1.60%	0.50%
C44S	28.00%	27.20%	-0.80%	-0.80%	-0.50%
C117S	29.30%	30.90%	1.60%	0.50%	3.20%
C128S	28.80%	27.80%	-1.00%	0.00%	0.10%
C145S	24.60%	26.60%	2.00%	-4.20%	-1.10%
C156S	27.90%	27.90%	0.00%	-0.90%	0.20%
C300S	27.70%	27.90%	0.20%	-1.10%	0.20%
K61A	28.50%	27.30%	-1.20%	-0.30%	-0.40%
K61A_C22S	28.80%	27.60%	-1.20%	0.00%	-0.10%
K61A_C44S	29.10%	27.40%	-1.70%	0.30%	-0.30%
K61A_C22S_C44S	27.20%	29.30%	2.10%	-1.60%	1.60%
C22S_C44S	28.30%	26.80%	-1.50%	-0.50%	-0.90%
Y54F	28.50%	27.90%	-0.60%	-0.30%	0.20%

CD spectra 195-260 nm	Antiparallel ß-strands				
	Reduced	Oxidized	Δ Oxidized to vs. Reduced	Δ Reduced rel. to WT	Δ Oxidized rel. to WT
WT	9.40%	10.90%	1.50%		
C22S	8.60%	10.80%	2.20%	-0.80%	-0.10%
C44S	10.10%	11.60%	1.50%	0.70%	0.70%
C117S	9.40%	8.70%	-0.70%	0.00%	-2.20%
C128S	9.70%	10.70%	1.00%	0.30%	-0.20%
C145S	14.40%	11.60%	-2.80%	5.00%	0.70%
C156S	10.30%	10.80%	0.50%	0.90%	-0.10%
C300S	10.40%	10.60%	0.20%	1.00%	-0.30%
K61A	9.60%	11.50%	1.90%	0.20%	0.60%
K61A_C22S	9.60%	11.40%	1.80%	0.20%	0.50%
K61A_C44S	9.50%	11.60%	2.10%	0.10%	0.70%
K61A_C22S_C44S	10.80%	10.10%	-0.70%	1.40%	-0.80%
C22S_C44S	10.10%	12.30%	2.20%	0.70%	1.40%
Y54F	9.80%	11.10%	1.30%	0.40%	0.20%

[1] For clarity, relative fractions of α-helices (upper table) or antiparallel ß-strands (lower table) are visualized in color-coded fashion (green: high amount, yellow: medium amount, red: low amount).

Supplementary Table 5. Redox-dependent melting temperatures of M^{pro} wild-type and variants as analyzed by CD spectroscopy in color-coded representation [1].

Thermal Unfolding	Reduced T_m	Oxidized T_m	ΔT_m Oxidized vs. Reduced	ΔT_m Reduced Relative to WT	ΔT_m Oxidized Relative to WT
WT	54.22 ± 0.06	48.05 ± 0.17	-6.17 ± 0.23	/	/
**SONOS variants**					
C22S	52.17 ± 0.06	45.80 ± 0.13	-6.36 ± 0.19	-2.05 ± 0.12	-2.25 ± 0.30
C44S	52.55 ± 0.06	46.78 ± 0.15	-5.77 ± 0.21	-1.67 ± 0.12	-1.27 ± 0.32
C22S_C44S	51.08 ± 0.07	45.43 ± 0.19	-5.65 ± 0.26	-3.14 ± 0.13	-2.62 ± 0.36
Y54F	53.23 ± 0.07	46.49 ± 0.12	-6.74 ± 0.19	-0.98 ± 0.13	-1.56 ± 0.29
K61A	53.70 ± 0.10	48.86 ± 0.16	-4.84 ± 0.26	-0.52 ± 0.16	0.81 ± 0.33
K61A_C22S	52.87 ± 0.08	48.34 ± 0.32	-4.53 ± 0.41	-1.35 ± 0.15	0.29 ± 0.49
K61A_C44S	53.15 ± 0.09	49.79 ± 0.26	-3.36 ± 0.35	-1.06 ± 0.16	1.74 ± 0.42
K61A_C22S_C44S	51.70 ± 0.08	50.06 ± 0.18	-1.64 ± 0.26	-2.52 ± 0.15	2.01 ± 0.34
**Cysteine variants**					
C117S	51.39 ± 0.06	49.20 ± 0.12	-2.19 ± 0.18	-2.83 ± 0.13	1.15 ± 0.29
C128S	50.65 ± 0.12	48.16 ± 0.10	-2.49 ± 0.22	-3.57 ± 0.18	0.10 ± 0.27
C145S	54.64 ± 0.08	52.00 ± 0.08	-2.64 ± 0.15	0.42 ± 0.14	3.95 ± 0.24
C156S	53.99 ± 0.07	48.84 ± 0.17	-5.14 ± 0.24	-0.23 ± 0.13	0.79 ± 0.34
C300S	53.24 ± 0.07	49.79 ± 0.23	-3.45 ± 0.29	-0.98 ± 0.13	1.74 ± 0.39

[1] For clarity, melting temperatures for the reduced and oxidized proteins are visualized in color-coded fashion (green: high temperature, yellow: medium temperature, red: low temperature). An independent scaling was used for the differences between oxidized and reduced proteins.

Supplementary Table 6. Primers used for site-directed mutagenesis.

C16S	5´-P-AGTGGAAGGTtctATGGTACAGGTGACATG-3´ upr<br>5´-P-TTGCCGGACGGAAACGCC-3´lwr
C22S	5´-P-ACAGGTGACAtccGGCACCACAA-3´ upr<br>5´-P-ACCATACAACCTTCCACTTTGC-3´ lwr
C38S	5´-P-CGTAGTCTATtctCCTCGTCATGTC-3´upr<br>5´-P-TCGTCTAACCACAACCCA-3´ lwr
C44S	5´-P-TCATGTCATCtccACCTCTGAGG-3´ upr<br>5´-P-CGAGGGCAATAGACTACG-3´ lwr
C44A	5´-P-TCATGTCATCgccACCTCTGAGGAC-3´ upr<br>5´-P-CGAGGGCAATAGACTACG-3´ lwr
C85S	5´-P-CATGCAGAATtccGTCCTTAAAC-3´ upr<br>5´-P-CTATGACCAATAACGCGC-3´ lwr
C117S	5´-P-AGTGTTAGCGtccTATAACGGCA-3´ upr<br>5´-P-GAAAAGGTCTGACCAGGC-3´ lwr
C128S	5´-P-TGTGTATCAGtctGCTATGCGTCC-3´ upr<br>5´-P-CCAGAGGGACTGCCGTTA-3´ lwr
C145S	5´-P-TAATGGCAGCtctGGTTCGGTGG-3´ upr<br>5´-P-AGGAAGCTGCCTTTGATC-3´ lwr
C156S	5´-P-CGACTACGATagcGTTAGCTTCT-3´ upr<br>5´-P-ATGTTAAAGCCCACCGAAC-3´ lwr
C160S	5´-P-CGTTAGCTTCtccTATATGCACC-3´ upr<br>5´-P-CAATCGTAGTCGATGTTAAAG-3´ lwr
C265S	5´-P-GCTGGATATGtctGCCAGTCTGAAAG-3´ upr<br>5´-P-ACAGCAATGCCCGTCTGT-3´ lwr
C300S	5´-P-GGTGCGTCAGtctAGCGGTGTCA-3´ upr<br>5´-P-ACATCGAAGGGAGTGAACTCATC-3´ lwr
K61A	5´-P-CCTGATCCGCgcaTCCAACCACA-3´ upr<br>5´-P- AGATCTTCGTAATTCGGATTG-3´ lwr
Y54F	5´-P- CAATCCGAATttcGAAGATCTCCTG-3´ upr<br>5´-P- AGCATGTCCTCAGAGGTG-3´ lwr

References *Supplementary Information*

SI 1) Weiser, J., Shenkin, P. S., & Still, W. C. (1999) Approximate atomic surfaces from linear combinations of pairwise overlaps (LCPO). *Journal of Computational Chemistry* 20, 217-230.
