# Supplementary material for: Multiple redox switches of the SARS-CoV-2 main protease in vitro provide opportunities for drug design
Source: Nat Commun. 2024 Jan 9;15:411. doi: 10.1038/s41467-023-44621-0 (PMC10776599; doi:10.1038/s41467-023-44621-0)
Supplement: Supplementary file 3 — Description of Additional Supplementary Files [file 41467_2023_44621_MOESM3_ESM.pdf]

Description of Additional Supplementary Files

File Name: Supplementary Data 1
Description: Analytical gel filtration experiments for SARS-CoV-2 M^{pro} wild-type and variants as-isolated, reduced, oxidized and re-reduced.

File Name: Supplementary Data 2
Description: Far-UV CD (Circular Dichroism) spectra for SARS-CoV-2 M^{pro} wild-type and variants in the reduced and oxidized forms before and after thermal unfolding.

File Name: Supplementary Data 3
Description: Far-UV CD-based thermal unfolding experiments for SARS-CoV-2 M^{pro} wildtype and variants in the reduced and oxidized form.

File Name: Supplementary Data 4
Description: Bifunctional crosslinkers tested to inhibit SARS-CoV-2 M^{pro} wild-type and their impact on the oligomeric equilibrium of M^{pro}.
