# Supplementary material for: Multiple redox switches of the SARS-CoV-2 main protease in vitro provide opportunities for drug design
Source: Nat Commun. 2024 Jan 9;15:411. doi: 10.1038/s41467-023-44621-0 (PMC10776599; doi:10.1038/s41467-023-44621-0)
Supplement: Supplementary file 5 — Supplementary Data 2 [file 41467_2023_44621_MOESM5_ESM.pdf]

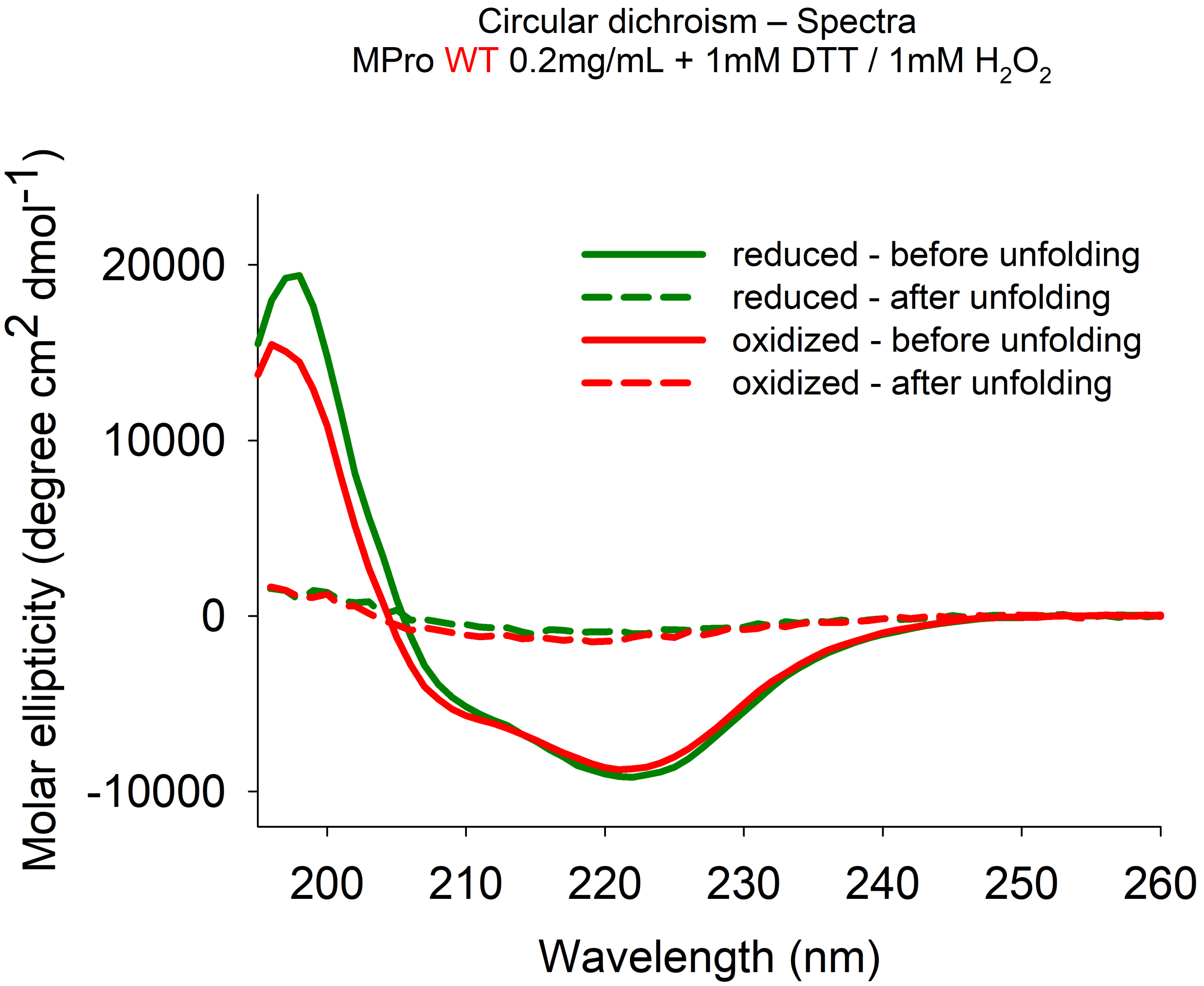
Circular dichroism – Spectra
MPro WT 0.2mg/mL + 1mM DTT / 1mM H_2O_2
Molar ellipticity (degree cm^2 dmol^{-1})
20000
10000
0
-10000
200
210
220
230
240
250
260
Wavelength (nm)
reduced - before unfolding
reduced - after unfolding
oxidized - before unfolding
oxidized - after unfolding

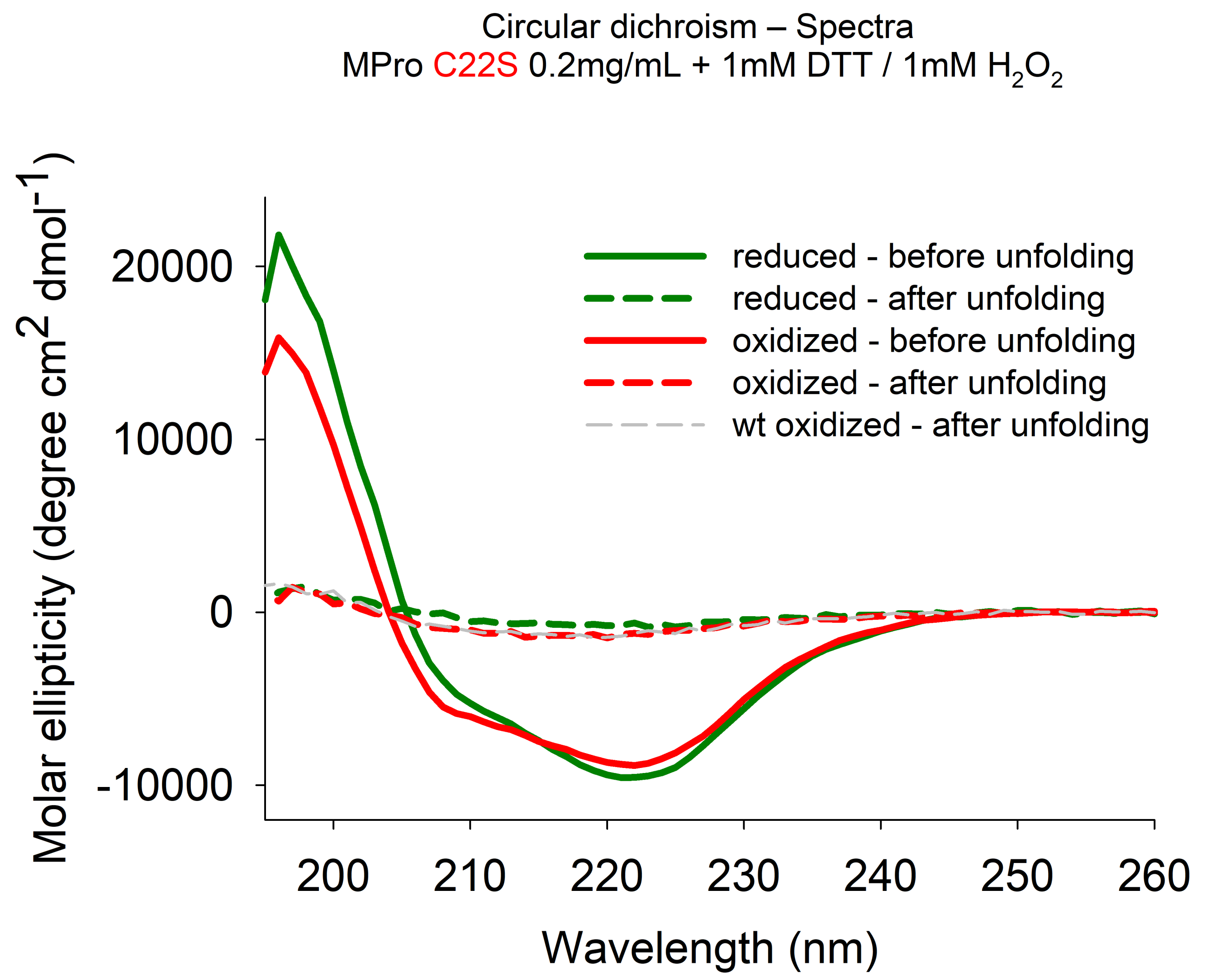
Circular dichroism – Spectra
MPro C22S 0.2mg/mL + 1mM DTT / 1mM H2O2
Molar ellipticity (degree cm2 dmol-1)
20000
10000
0
-10000
200
210
220
230
240
250
260
Wavelength (nm)
reduced - before unfolding
reduced - after unfolding
oxidized - before unfolding
oxidized - after unfolding
wt oxidized - after unfolding

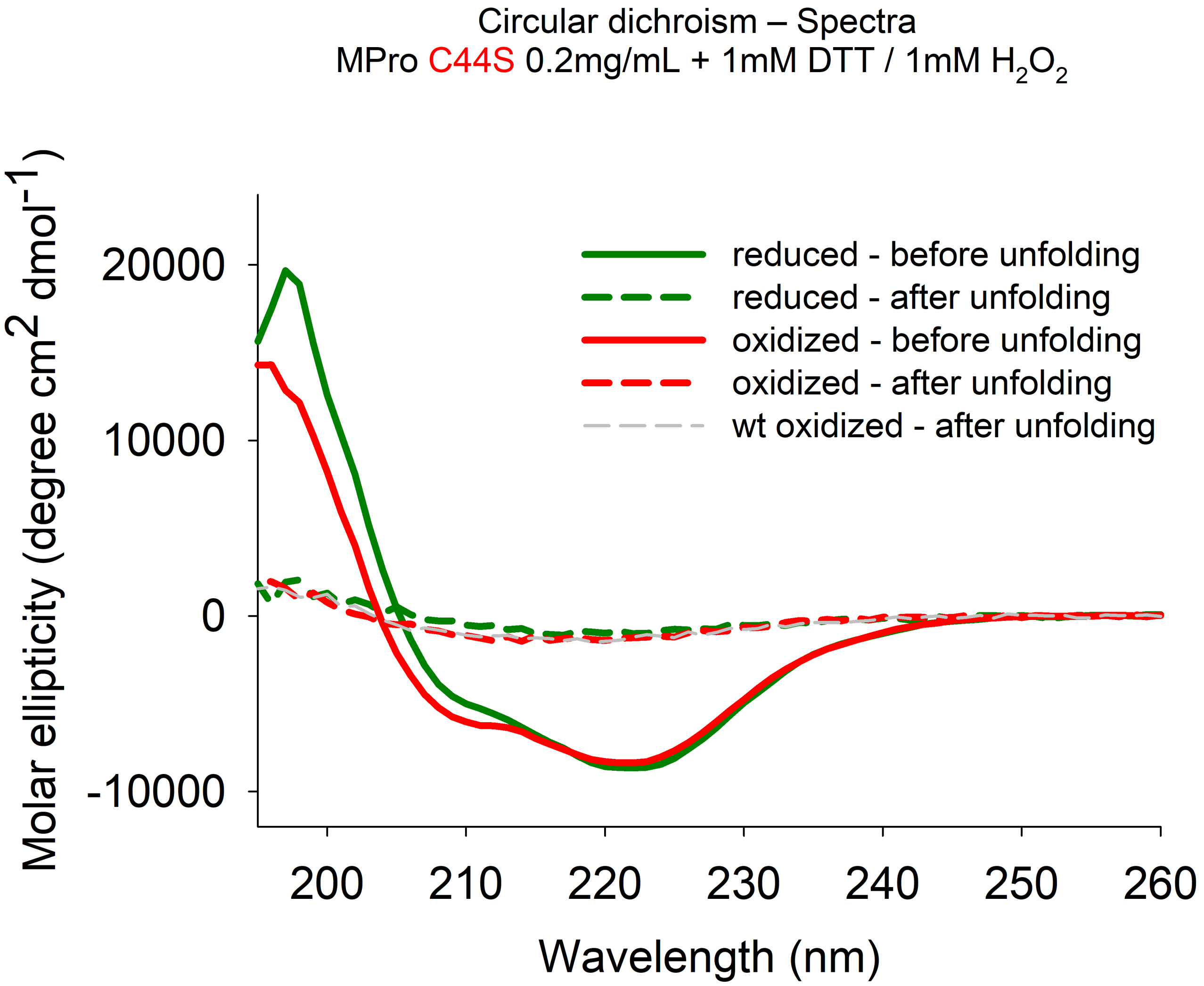

Circular dichroism – Spectra
MPro C44S 0.2mg/mL + 1mM DTT / 1mM H2O2
Molar ellipticity (degree cm2 dmol-1)
20000
10000
0
-10000
200
210
220
230
240
250
260
Wavelength (nm)
reduced - before unfolding
reduced - after unfolding
oxidized - before unfolding
oxidized - after unfolding
wt oxidized - after unfolding

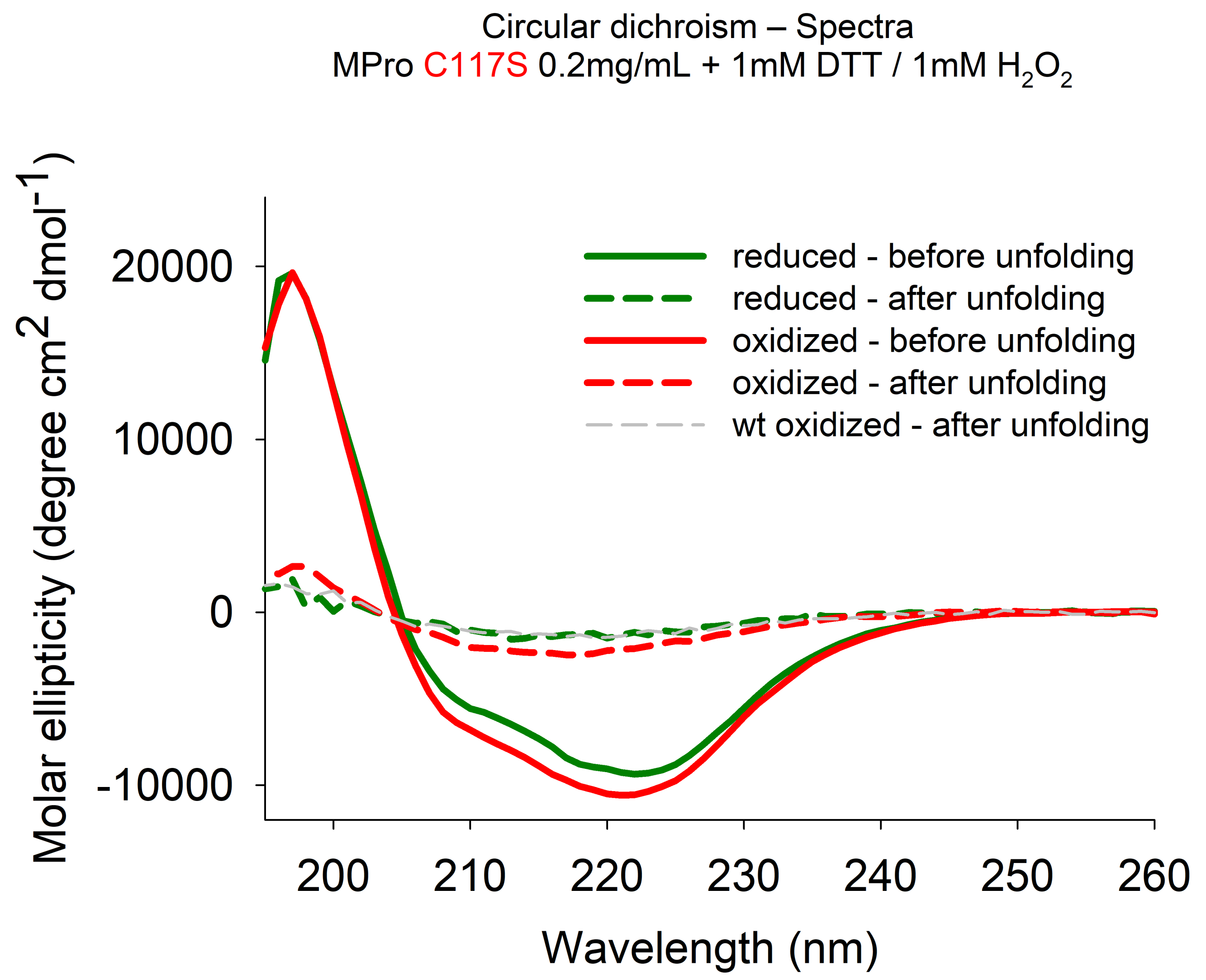
Circular dichroism – Spectra
MPro C117S 0.2mg/mL + 1mM DTT / 1mM H2O2
Molar ellipticity (degree cm2 dmol-1)
20000
10000
0
-10000
200
210
220
230
240
250
260
Wavelength (nm)
reduced - before unfolding
reduced - after unfolding
oxidized - before unfolding
oxidized - after unfolding
wt oxidized - after unfolding

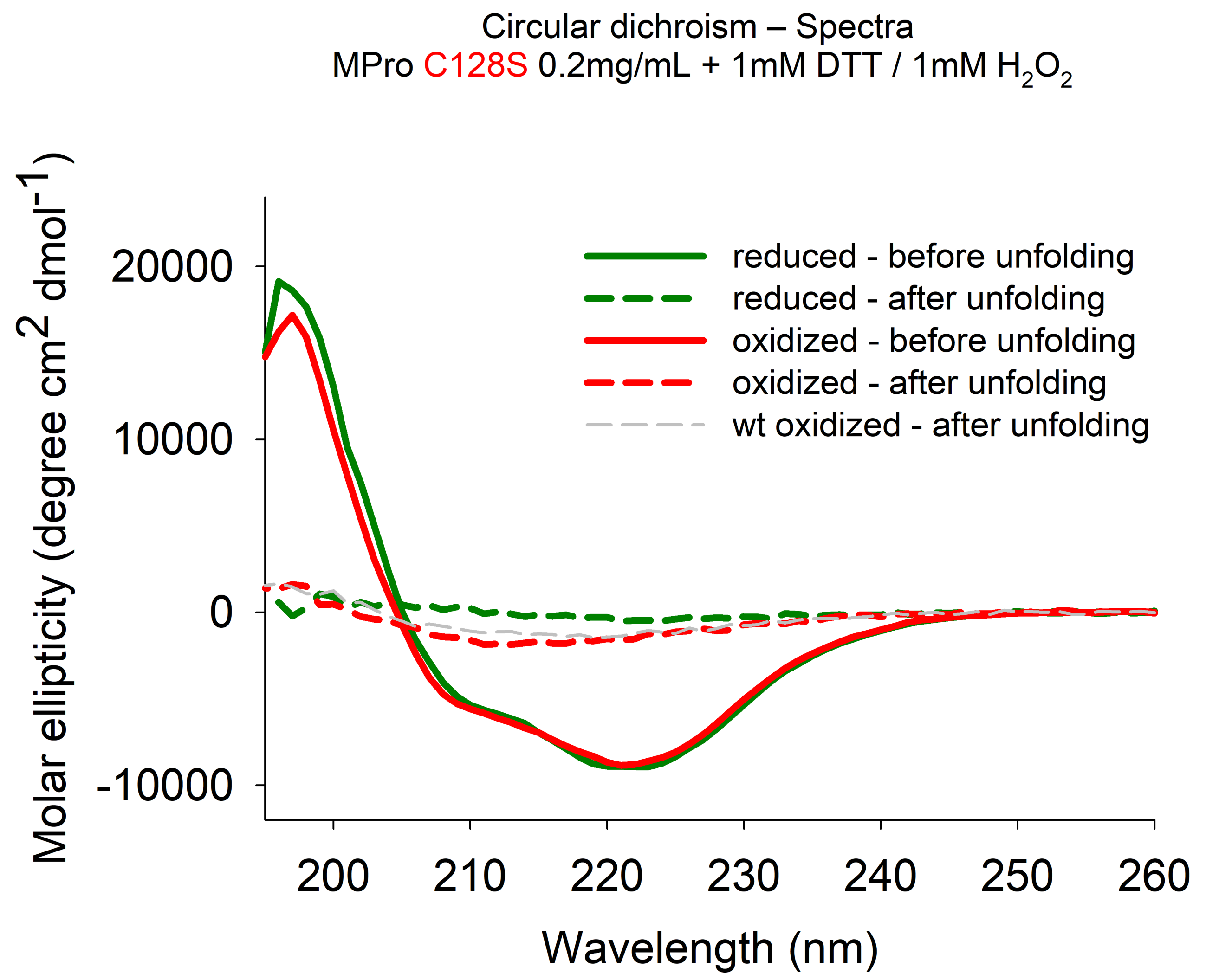
Circular dichroism – Spectra
MPro C128S 0.2mg/mL + 1mM DTT / 1mM H_2O_2
Molar ellipticity (degree cm^2 dmol^{-1})
20000
10000
0
-10000
200
210
220
230
240
250
260
Wavelength (nm)
reduced - before unfolding
reduced - after unfolding
oxidized - before unfolding
oxidized - after unfolding
wt oxidized - after unfolding

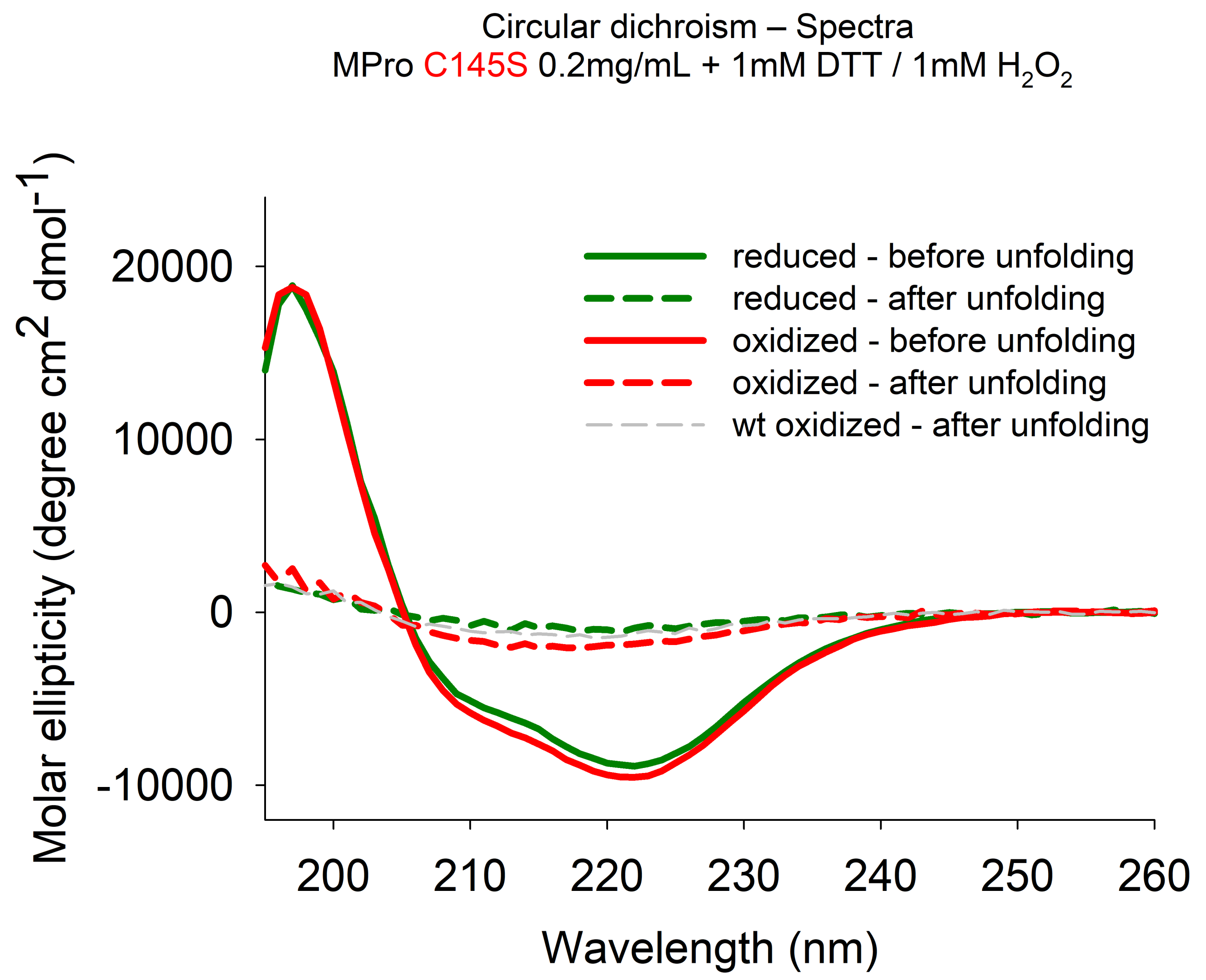
Circular dichroism – Spectra
MPro C145S 0.2mg/mL + 1mM DTT / 1mM H2O2
Molar ellipticity (degree cm2 dmol-1)
20000
10000
0
-10000
200
210
220
230
240
250
260
Wavelength (nm)
reduced - before unfolding
reduced - after unfolding
oxidized - before unfolding
oxidized - after unfolding
wt oxidized - after unfolding

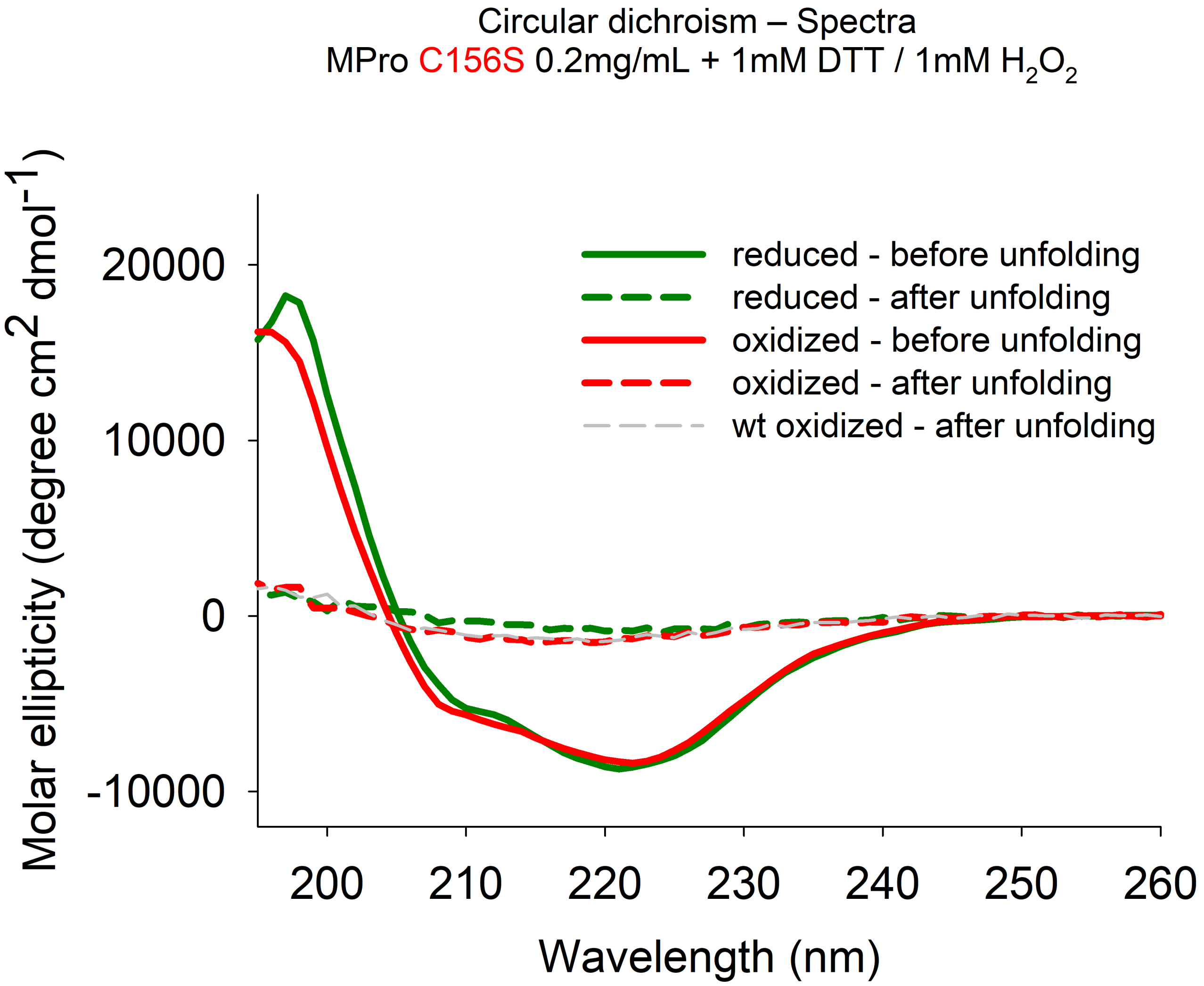
Circular dichroism – Spectra
MPro C156S 0.2mg/mL + 1mM DTT / 1mM H_2O_2
Molar ellipticity (degree cm^2 dmol^{-1})
20000
10000
0
-10000
200
210
220
230
240
250
260
Wavelength (nm)
reduced - before unfolding
reduced - after unfolding
oxidized - before unfolding
oxidized - after unfolding
wt oxidized - after unfolding

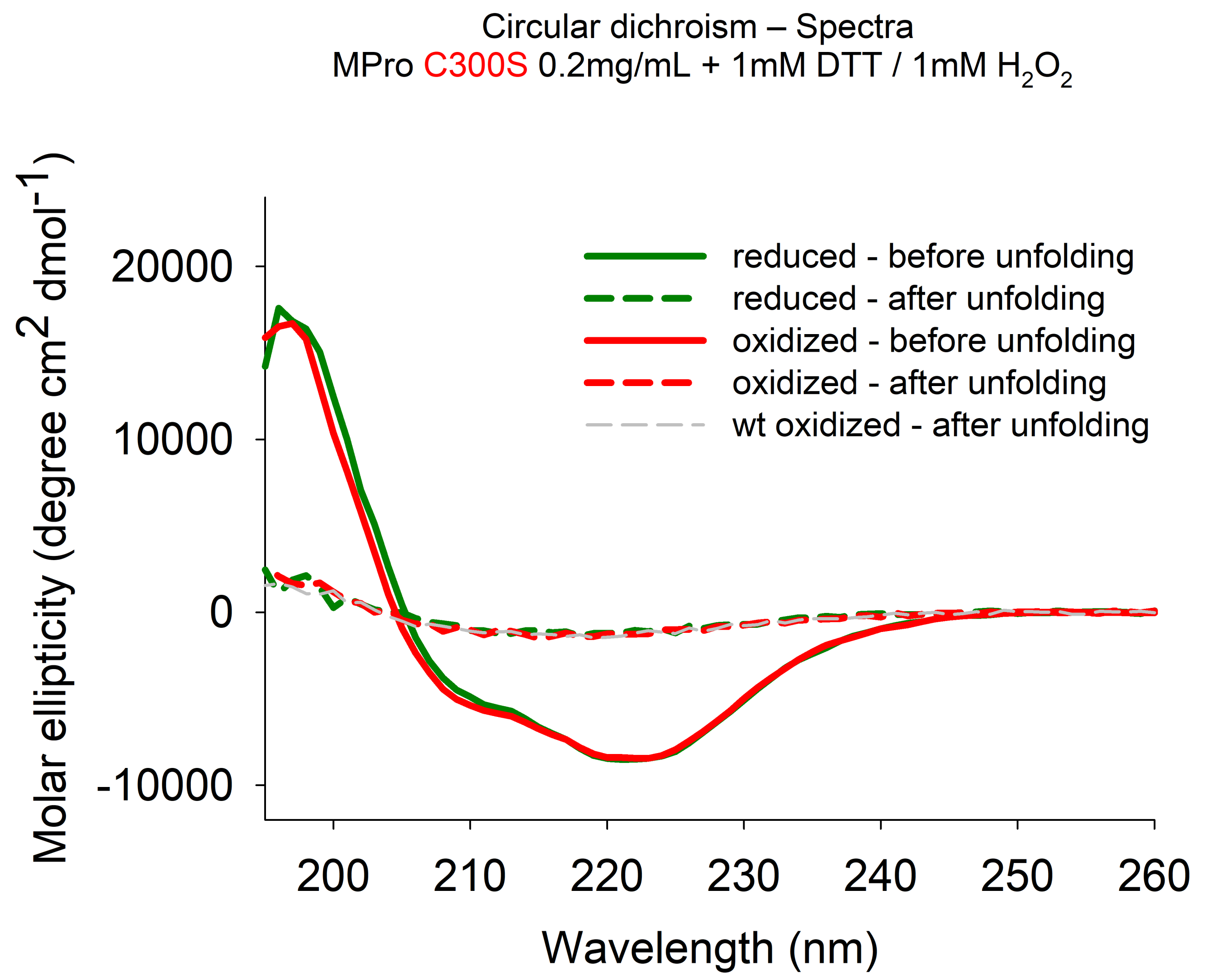
Circular dichroism – Spectra
MPro C300S 0.2mg/mL + 1mM DTT / 1mM H_2O_2
Molar ellipticity (degree cm^2 dmol^{-1})
20000
10000
0
-10000
200
210
220
230
240
250
260
Wavelength (nm)
reduced - before unfolding
reduced - after unfolding
oxidized - before unfolding
oxidized - after unfolding
wt oxidized - after unfolding

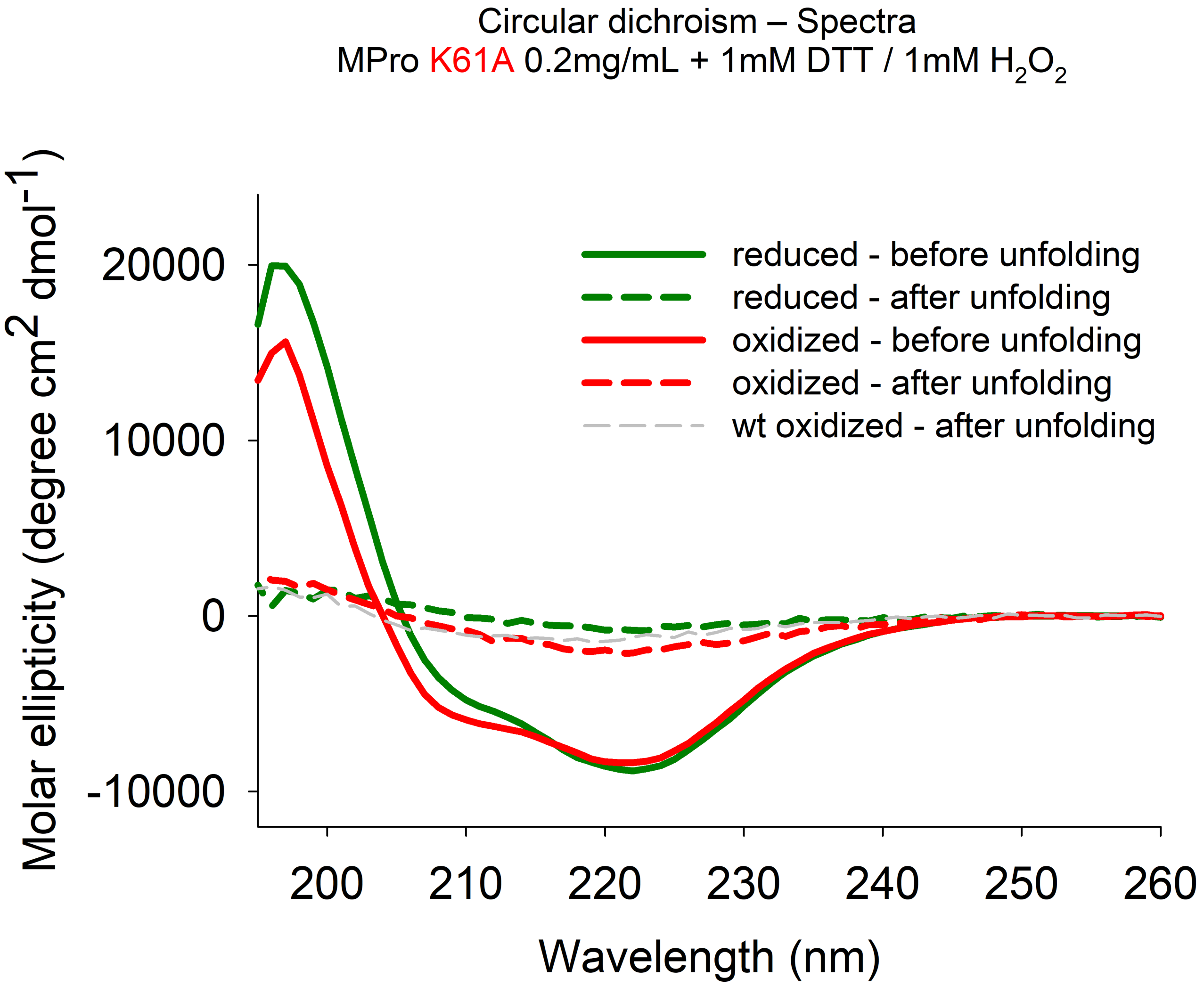

Circular dichroism – Spectra
MPro K61A 0.2mg/mL + 1mM DTT / 1mM H_2O_2
Molar ellipticity (degree cm^2 dmol^{-1})
20000
10000
0
-10000
200
210
220
230
240
250
260
Wavelength (nm)
reduced - before unfolding
reduced - after unfolding
oxidized - before unfolding
oxidized - after unfolding
wt oxidized - after unfolding

Circular dichroism – Spectra
MPro K61A_C22S 0.2mg/mL + 1mM DTT / 1mM H_2O_2

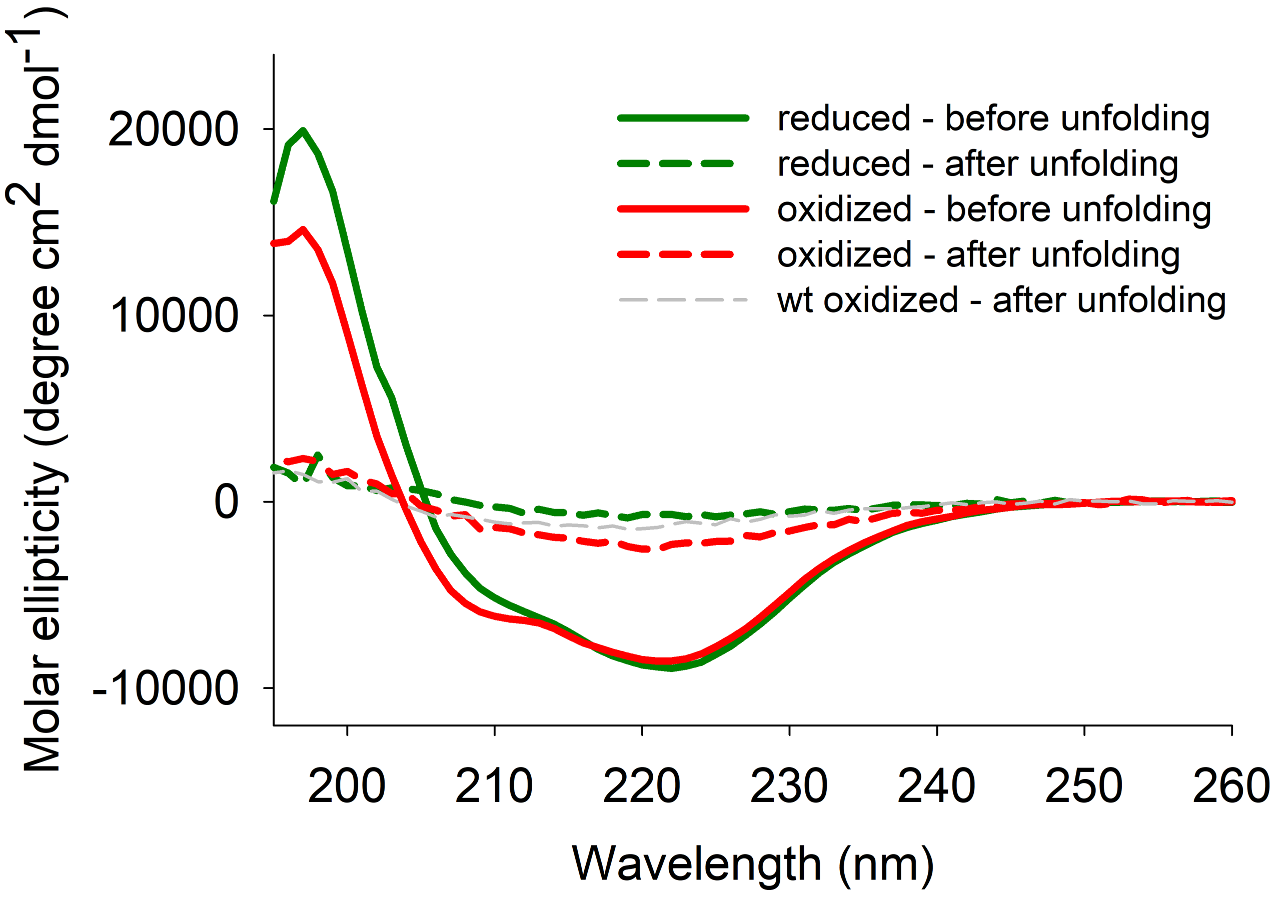

Circular dichroism – Spectra

MPro K61A_C44S 0.2mg/mL + 1mM DTT / 1mM H_2O_2

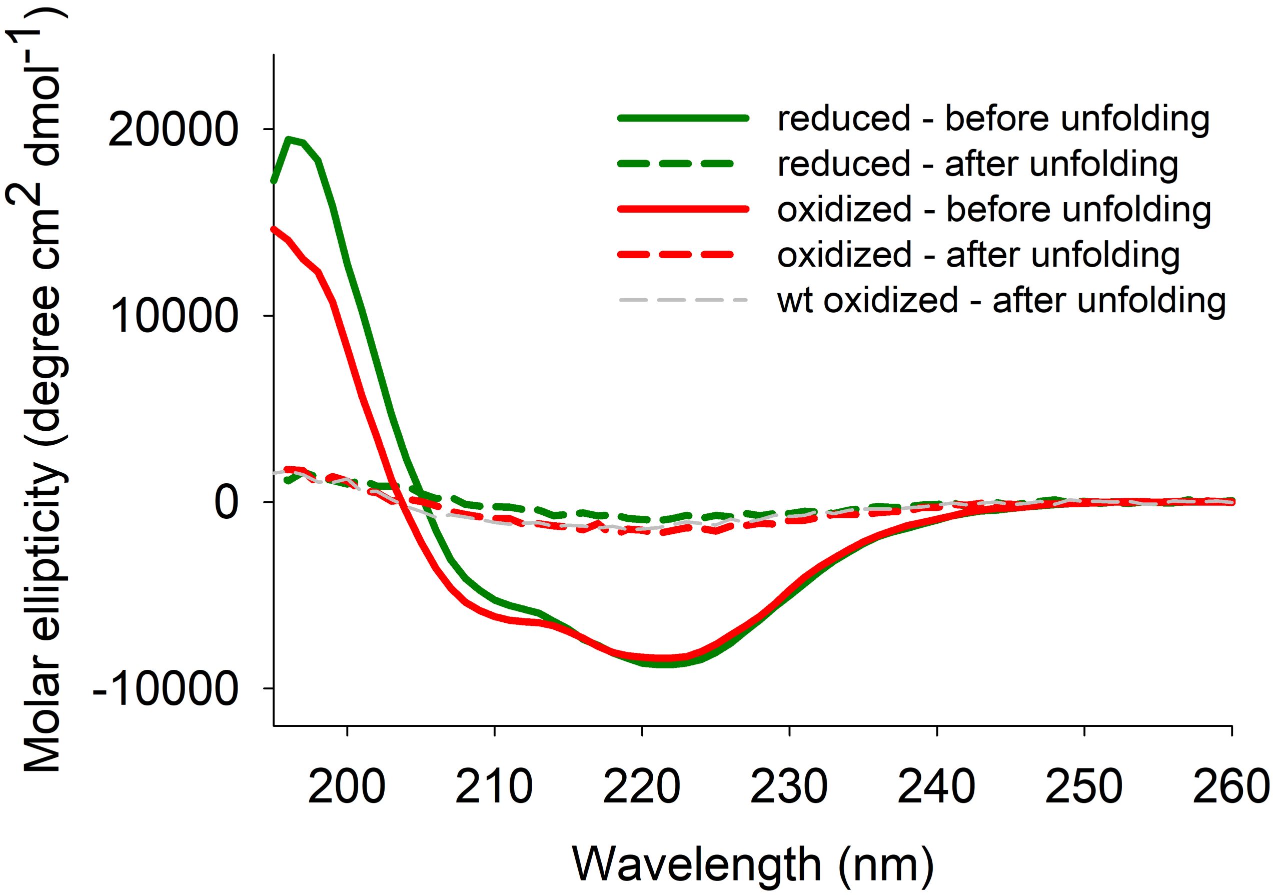

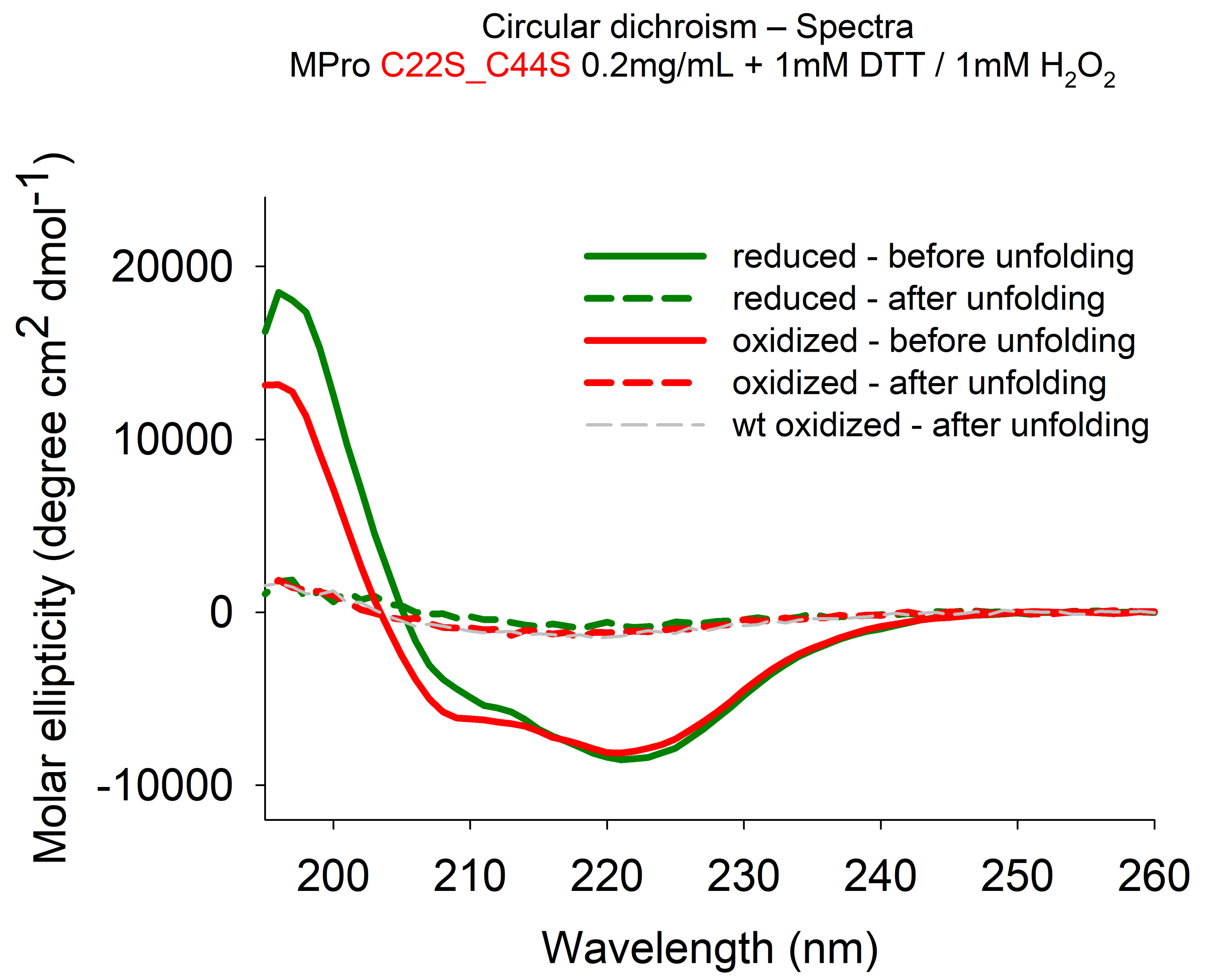
Circular dichroism – Spectra
MPro C22S_C44S 0.2mg/mL + 1mM DTT / 1mM H2O2
reduced - before unfolding
reduced - after unfolding
oxidized - before unfolding
oxidized - after unfolding
wt oxidized - after unfolding
Molar ellipticity (degree cm2 dmol-1)
20000
10000
0
-10000
200
210
220
230
240
250
260
Wavelength (nm)

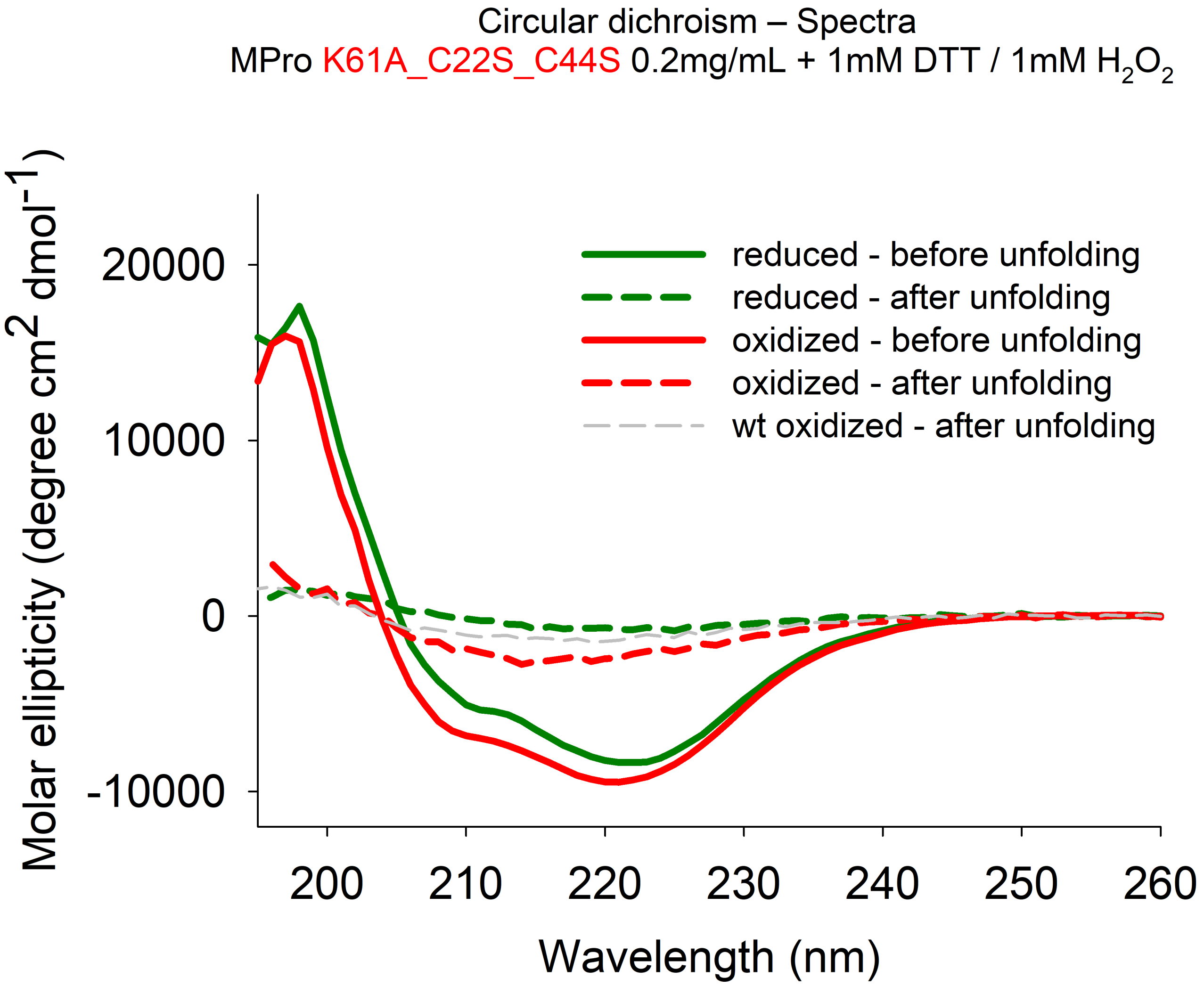
Circular dichroism – Spectra
MPro K61A_C22S_C44S 0.2mg/mL + 1mM DTT / 1mM H_2O_2
reduced - before unfolding
reduced - after unfolding
oxidized - before unfolding
oxidized - after unfolding
wt oxidized - after unfolding
Molar ellipticity (degree cm^2 dmol^{-1})
20000
10000
0
-10000
200
210
220
230
240
250
260
Wavelength (nm)

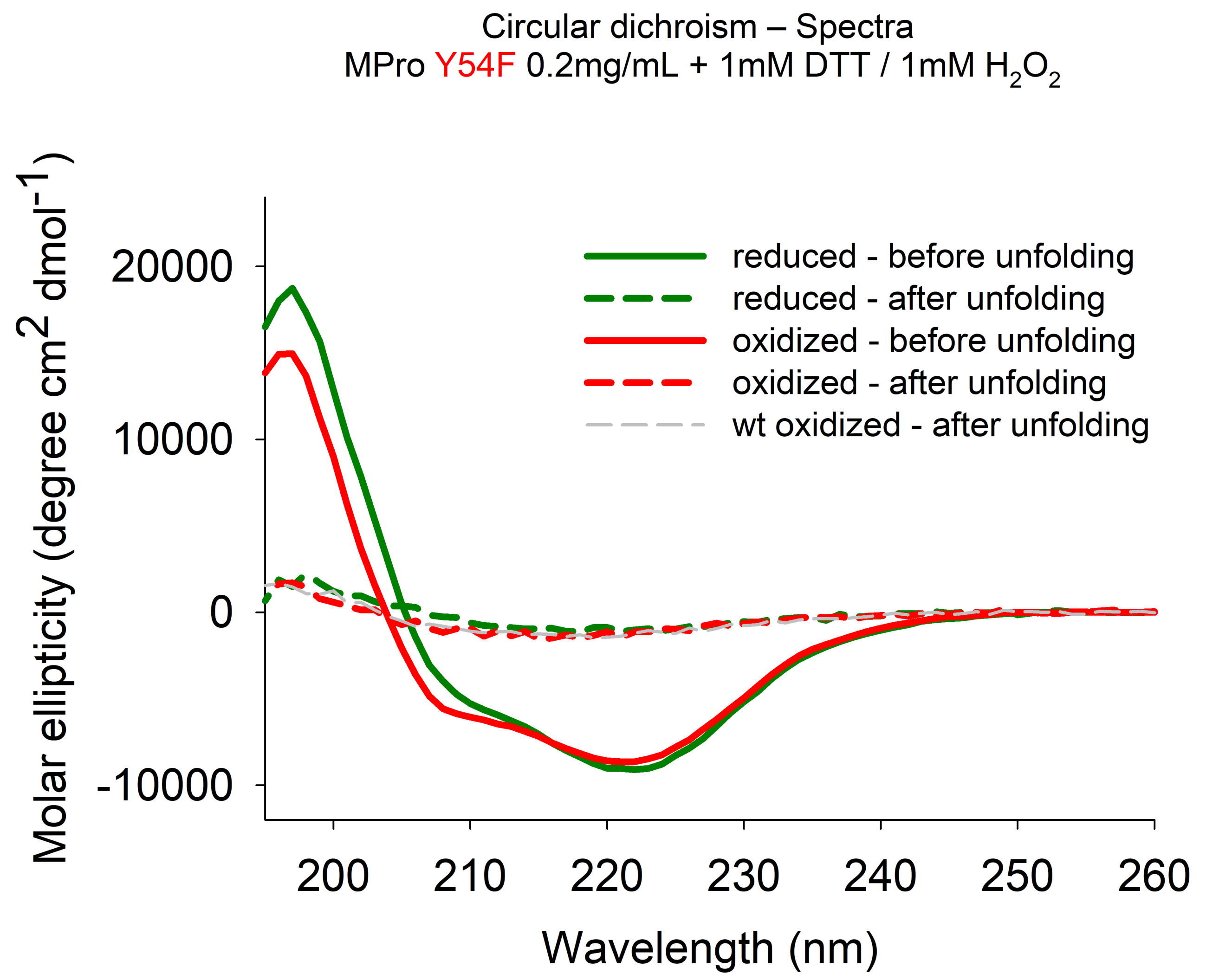
Circular dichroism – Spectra
MPro Y54F 0.2mg/mL + 1mM DTT / 1mM H_2O_2
Molar ellipticity (degree cm^2 dmol^{-1})
20000
10000
0
-10000
200
210
220
230
240
250
260
Wavelength (nm)
reduced - before unfolding
reduced - after unfolding
oxidized - before unfolding
oxidized - after unfolding
wt oxidized - after unfolding
