# Supplementary material for: Multiple redox switches of the SARS-CoV-2 main protease in vitro provide opportunities for drug design
Source: Nat Commun. 2024 Jan 9;15:411. doi: 10.1038/s41467-023-44621-0 (PMC10776599; doi:10.1038/s41467-023-44621-0)
Supplement: Supplementary file 6 — Supplementary Data 3 [file 41467_2023_44621_MOESM6_ESM.pdf]

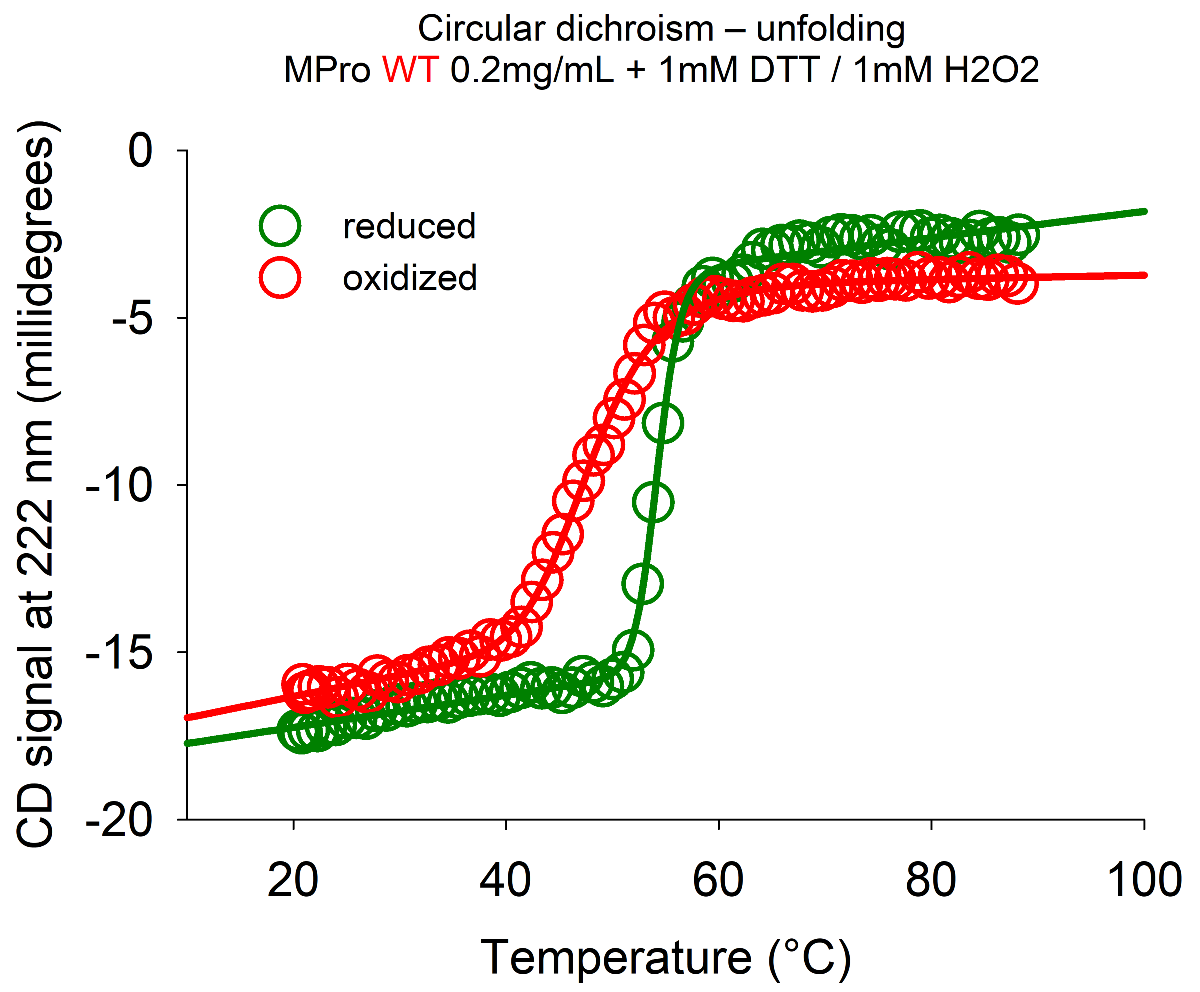
Circular dichroism – unfolding
MPro WT 0.2mg/mL + 1mM DTT / 1mM H2O2
reduced
oxidized
CD signal at 222 nm (millidegrees)
0
-5
-10
-15
-20
20
40
60
80
100
Temperature (°C)

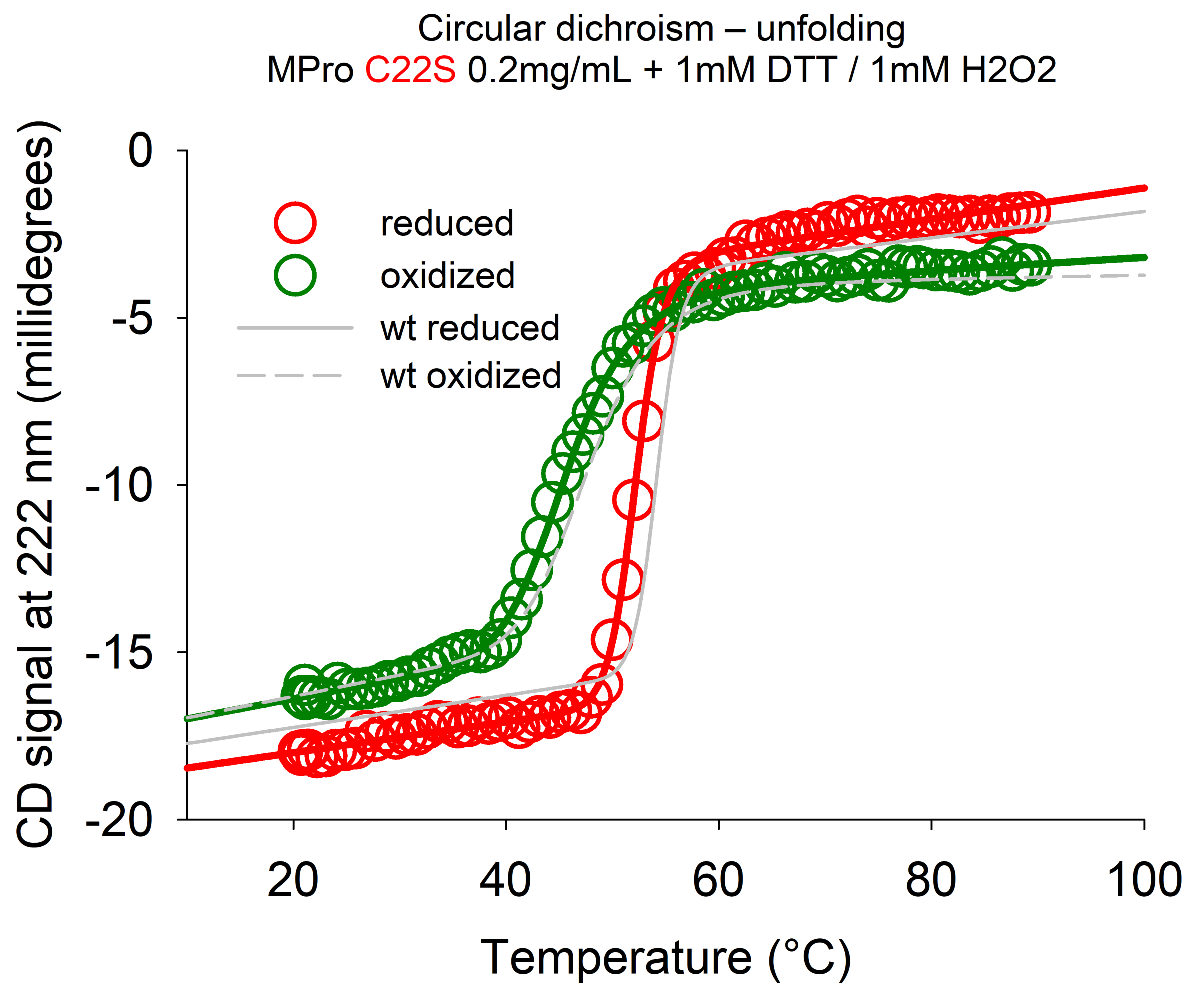

Circular dichroism – unfolding
MPro C22S 0.2mg/mL + 1mM DTT / 1mM H2O2
CD signal at 222 nm (millidegrees)
0
-5
-10
-15
-20
reduced
oxidized
wt reduced
wt oxidized
20
40
60
80
100
Temperature (°C)

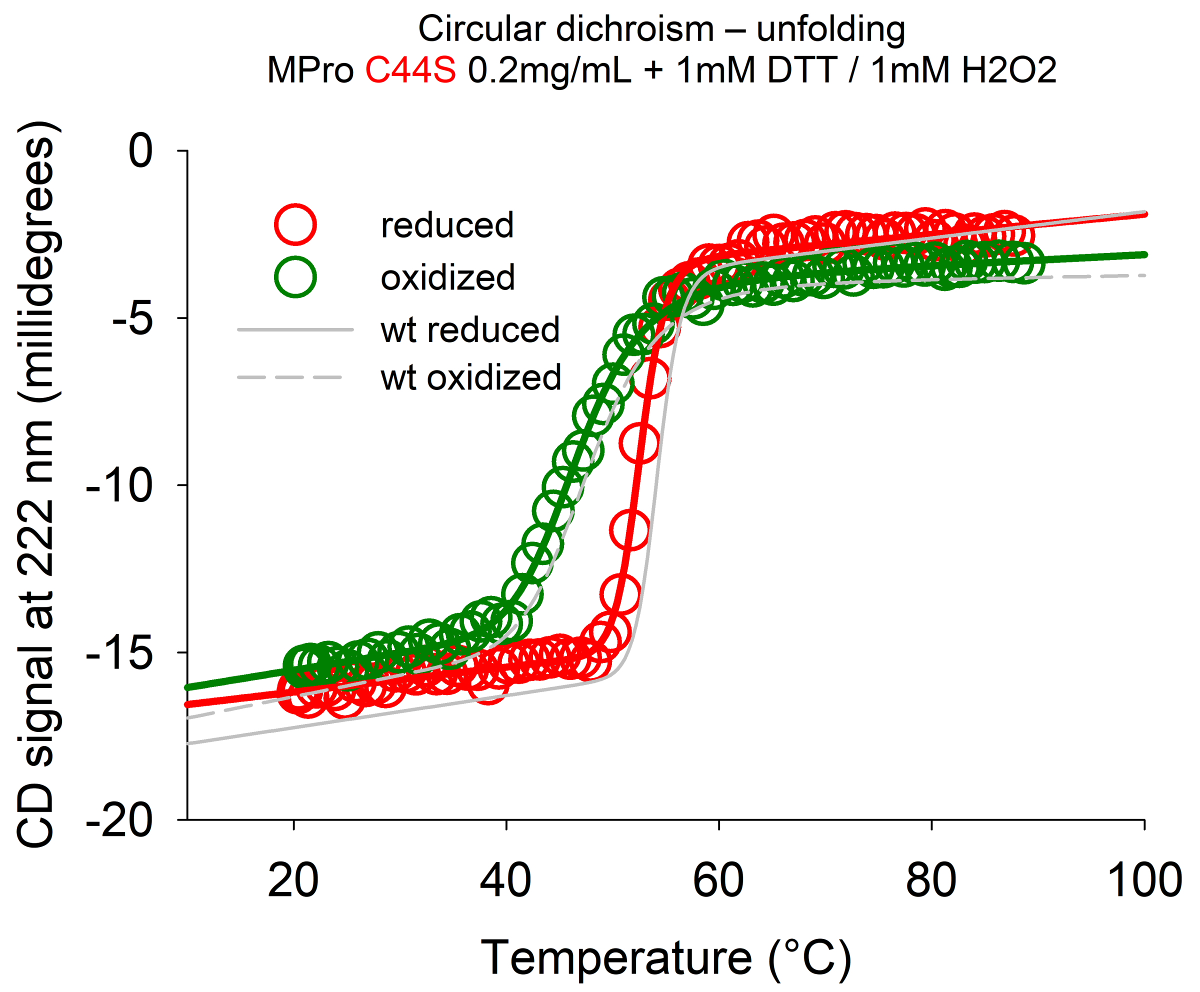

Circular dichroism – unfolding
MPro C44S 0.2mg/mL + 1mM DTT / 1mM H2O2
reduced
oxidized
wt reduced
wt oxidized
CD signal at 222 nm (millidegrees)
0
-5
-10
-15
-20
20
40
60
80
100
Temperature (°C)

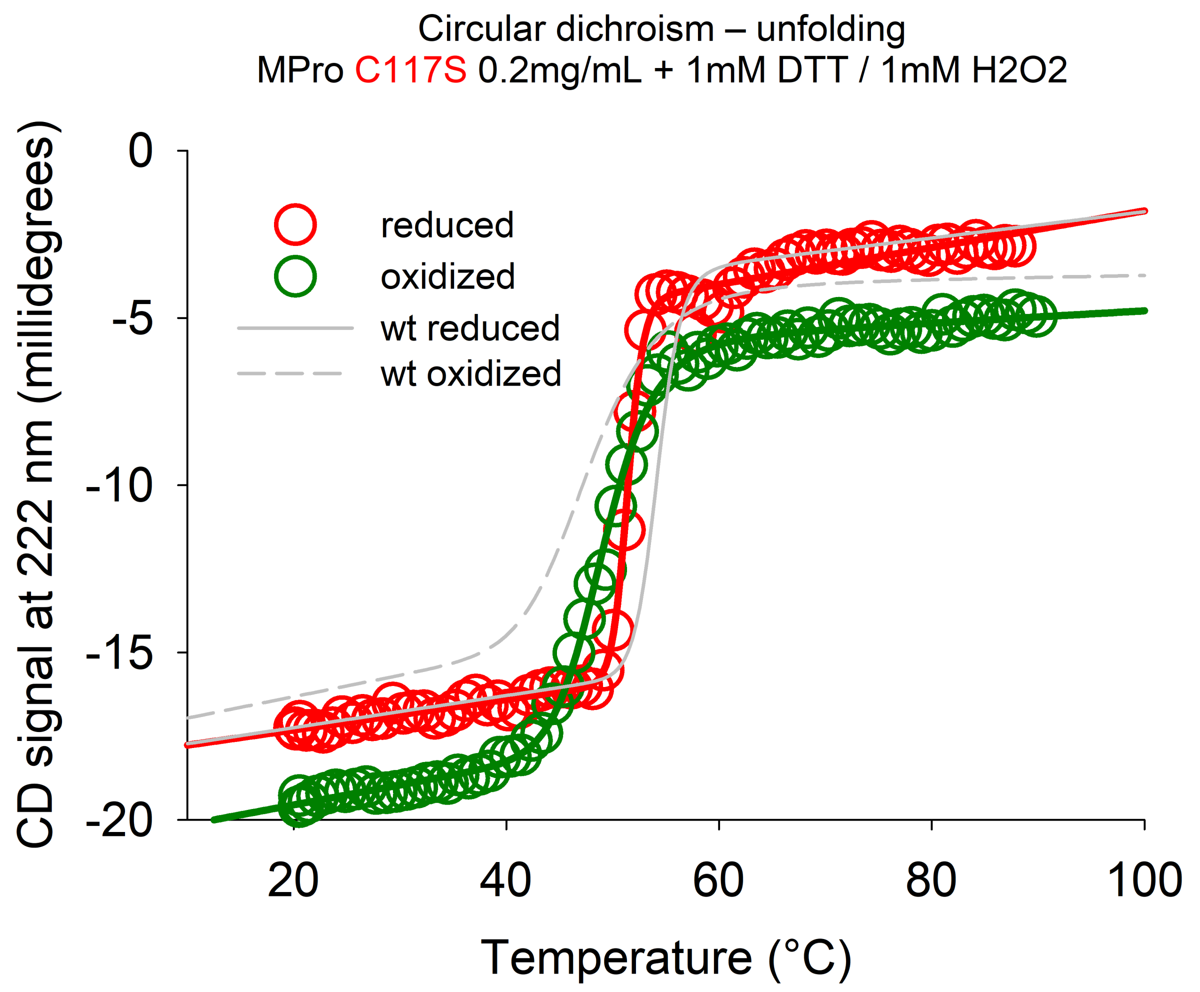

Circular dichroism – unfolding
MPro C117S 0.2mg/mL + 1mM DTT / 1mM H2O2
reduced
oxidized
wt reduced
wt oxidized
CD signal at 222 nm (millidegrees)
0
-5
-10
-15
-20
20
40
60
80
100
Temperature (°C)

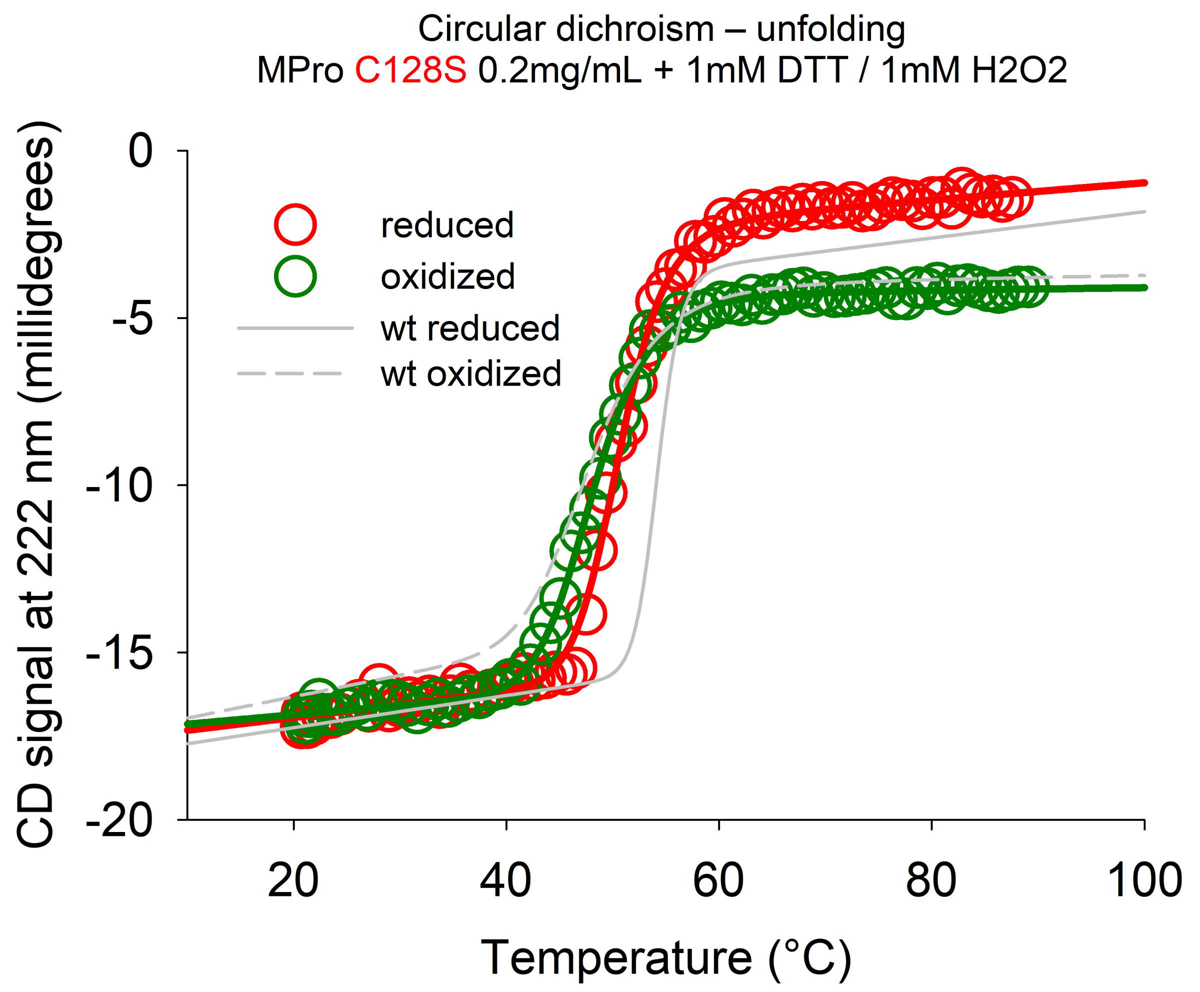

Circular dichroism – unfolding
MPro C128S 0.2mg/mL + 1mM DTT / 1mM H2O2
reduced
oxidized
wt reduced
wt oxidized
CD signal at 222 nm (millidegrees)
0
-5
-10
-15
-20
20
40
60
80
100
Temperature (°C)

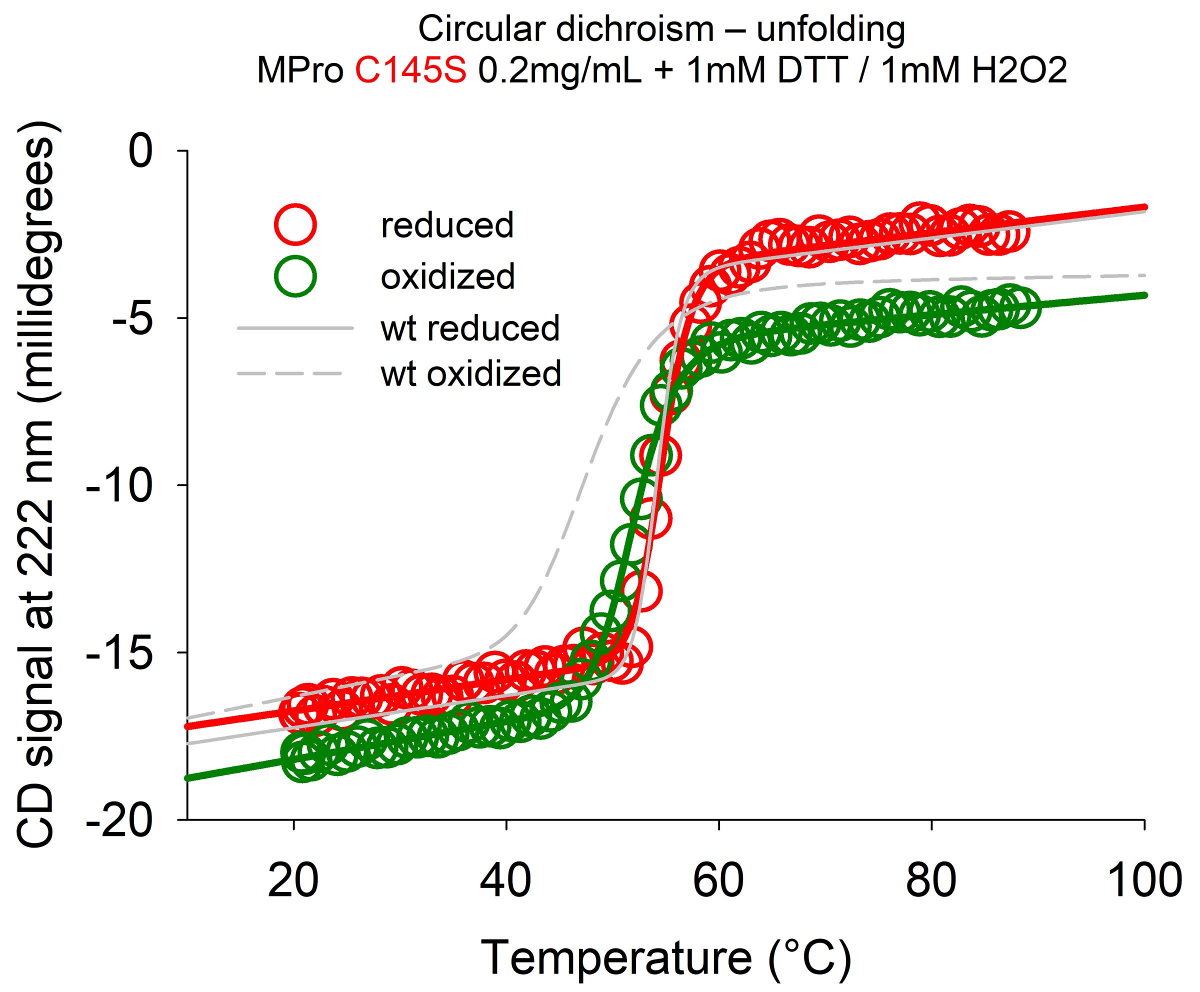

Circular dichroism – unfolding
MPro C145S 0.2mg/mL + 1mM DTT / 1mM H2O2
reduced
oxidized
wt reduced
wt oxidized
CD signal at 222 nm (millidegrees)
0
-5
-10
-15
-20
20
40
60
80
100
Temperature (°C)

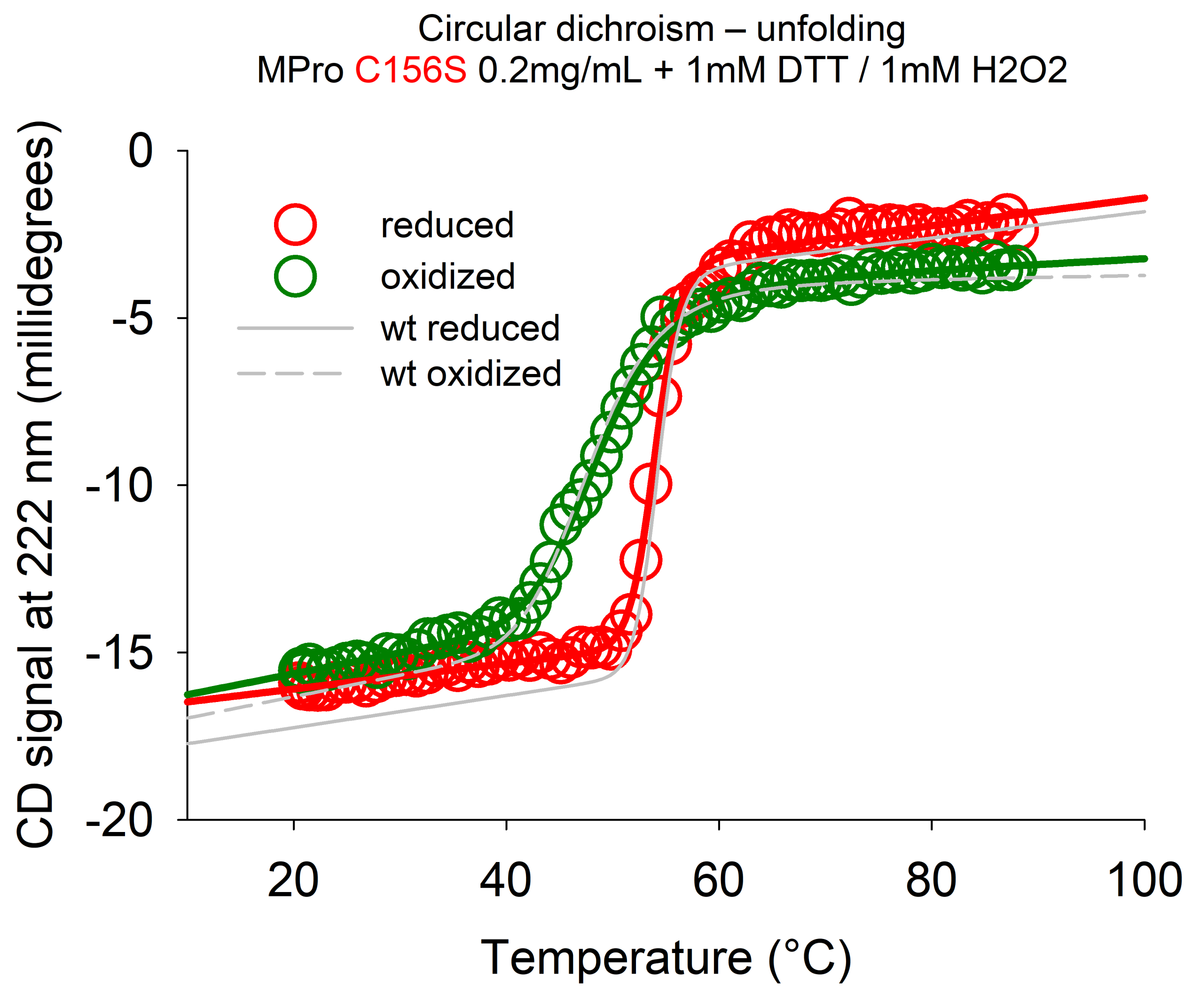

Circular dichroism – unfolding
MPro C156S 0.2mg/mL + 1mM DTT / 1mM H2O2
reduced
oxidized
wt reduced
wt oxidized
CD signal at 222 nm (millidegrees)
0
-5
-10
-15
-20
20
40
60
80
100
Temperature (°C)

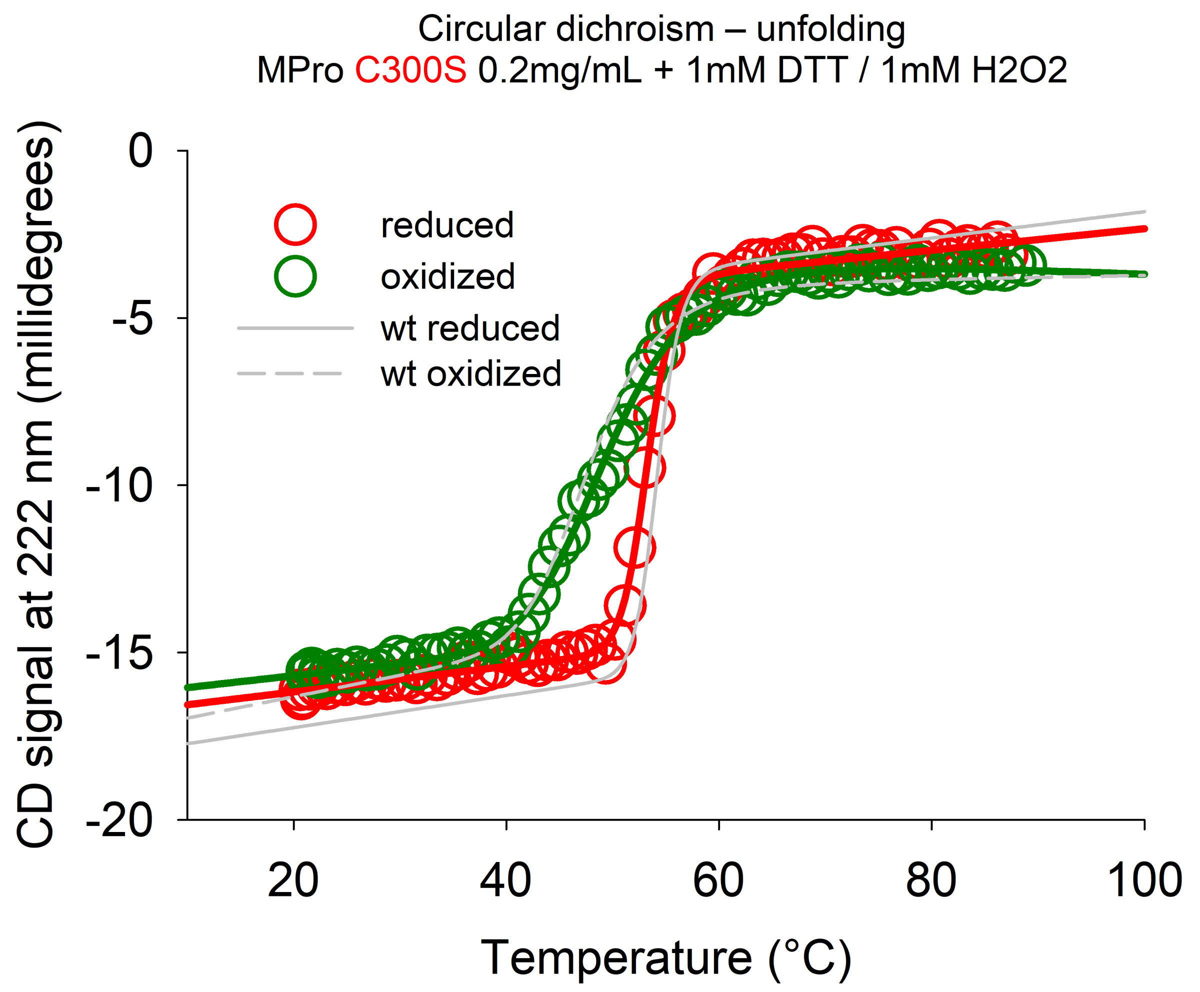

Circular dichroism – unfolding
MPro C300S 0.2mg/mL + 1mM DTT / 1mM H2O2
reduced
oxidized
wt reduced
wt oxidized
CD signal at 222 nm (millidegrees)
0
-5
-10
-15
-20
20
40
60
80
100
Temperature (°C)

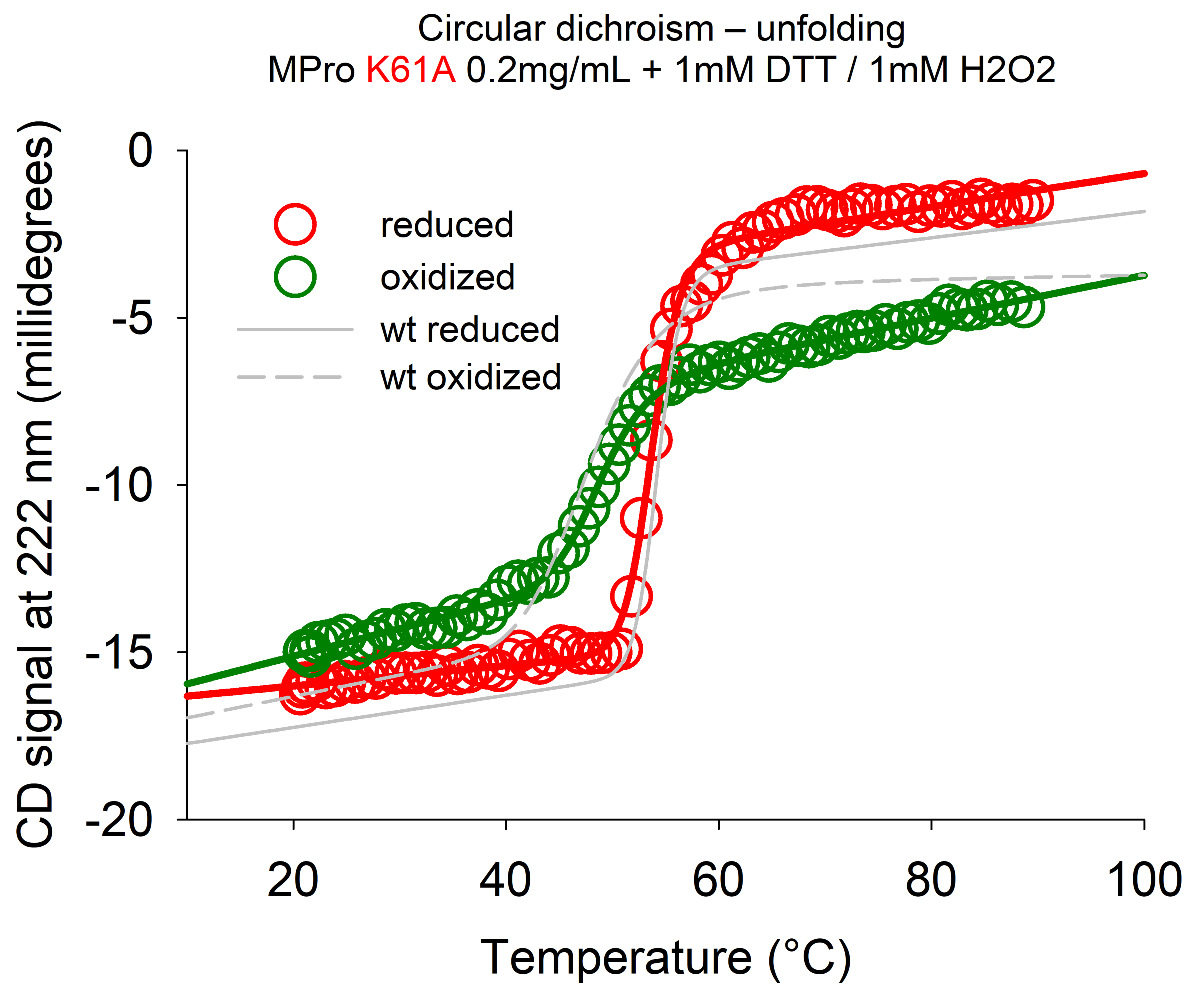

Circular dichroism – unfolding
MPro K61A 0.2mg/mL + 1mM DTT / 1mM H2O2
CD signal at 222 nm (millidegrees)
0
-5
-10
-15
-20
20
40
60
80
100
Temperature (°C)
reduced
oxidized
wt reduced
wt oxidized

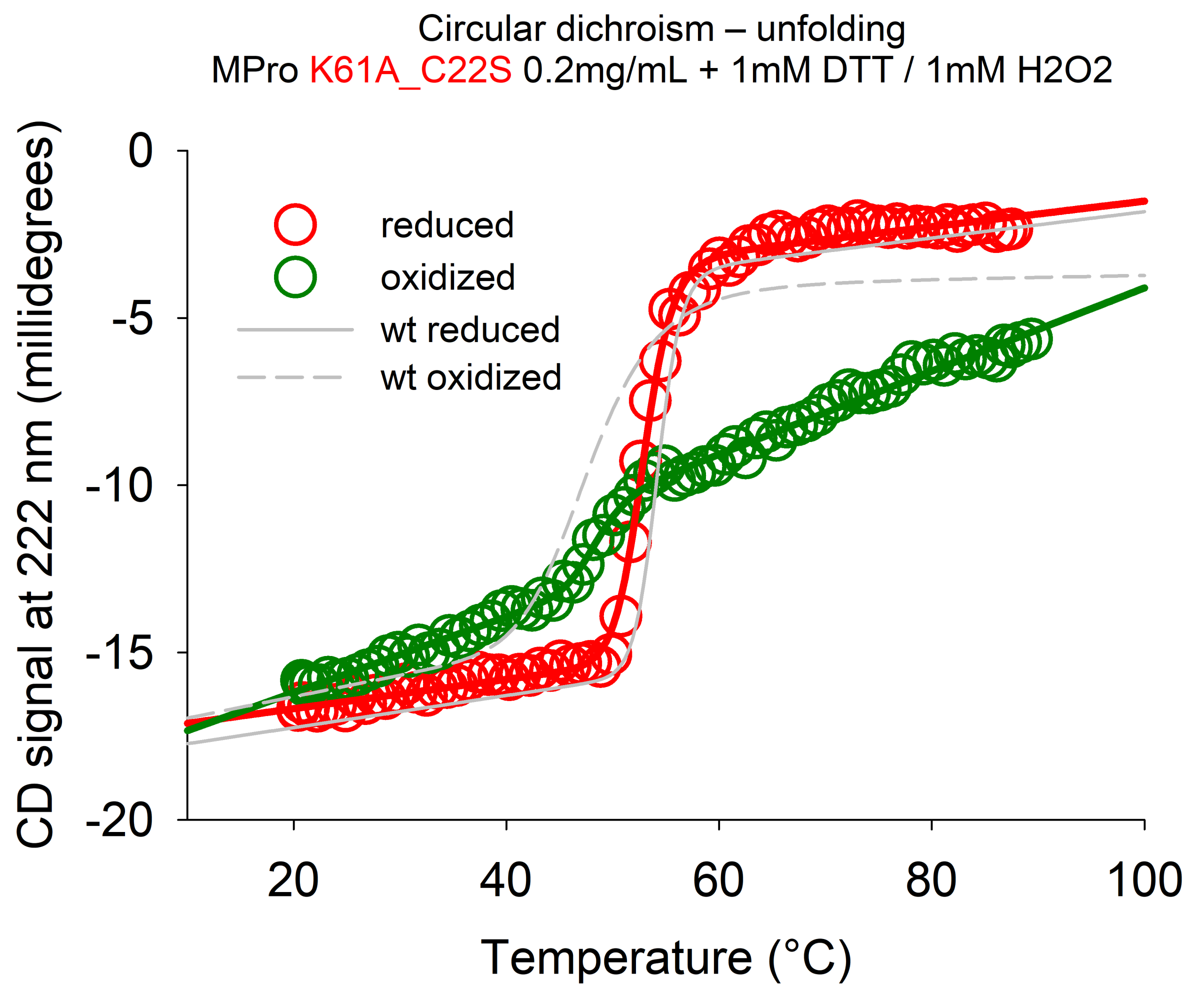

Circular dichroism – unfolding
MPro K61A_C22S 0.2mg/mL + 1mM DTT / 1mM H2O2
reduced
oxidized
wt reduced
wt oxidized
CD signal at 222 nm (millidegrees)
Temperature (°C)
0
-5
-10
-15
-20
20
40
60
80
100

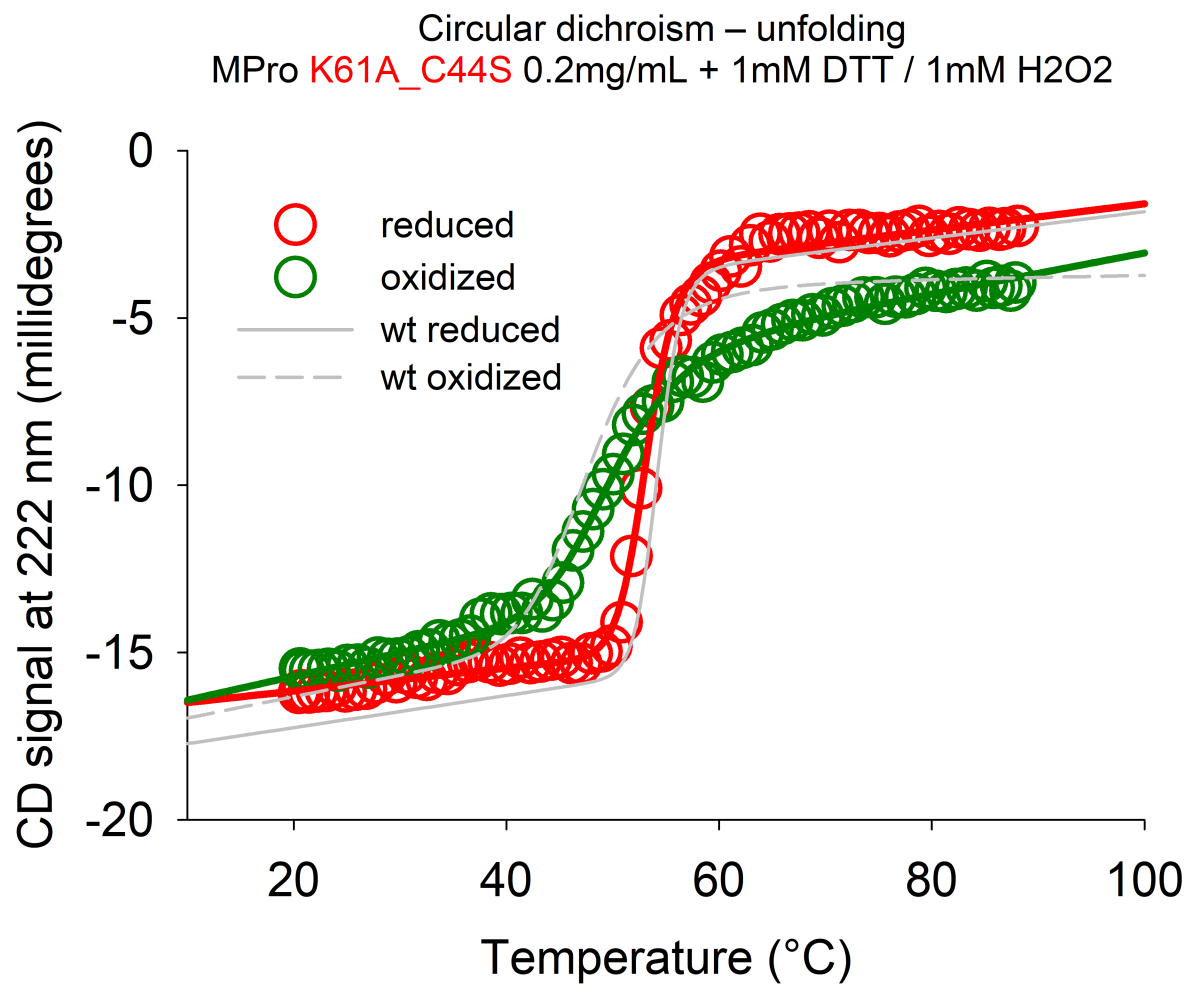

Circular dichroism – unfolding
MPro K61A_C44S 0.2mg/mL + 1mM DTT / 1mM H2O2
CD signal at 222 nm (millidegrees)
0
-5
-10
-15
-20
reduced
oxidized
wt reduced
wt oxidized
20
40
60
80
100
Temperature (°C)

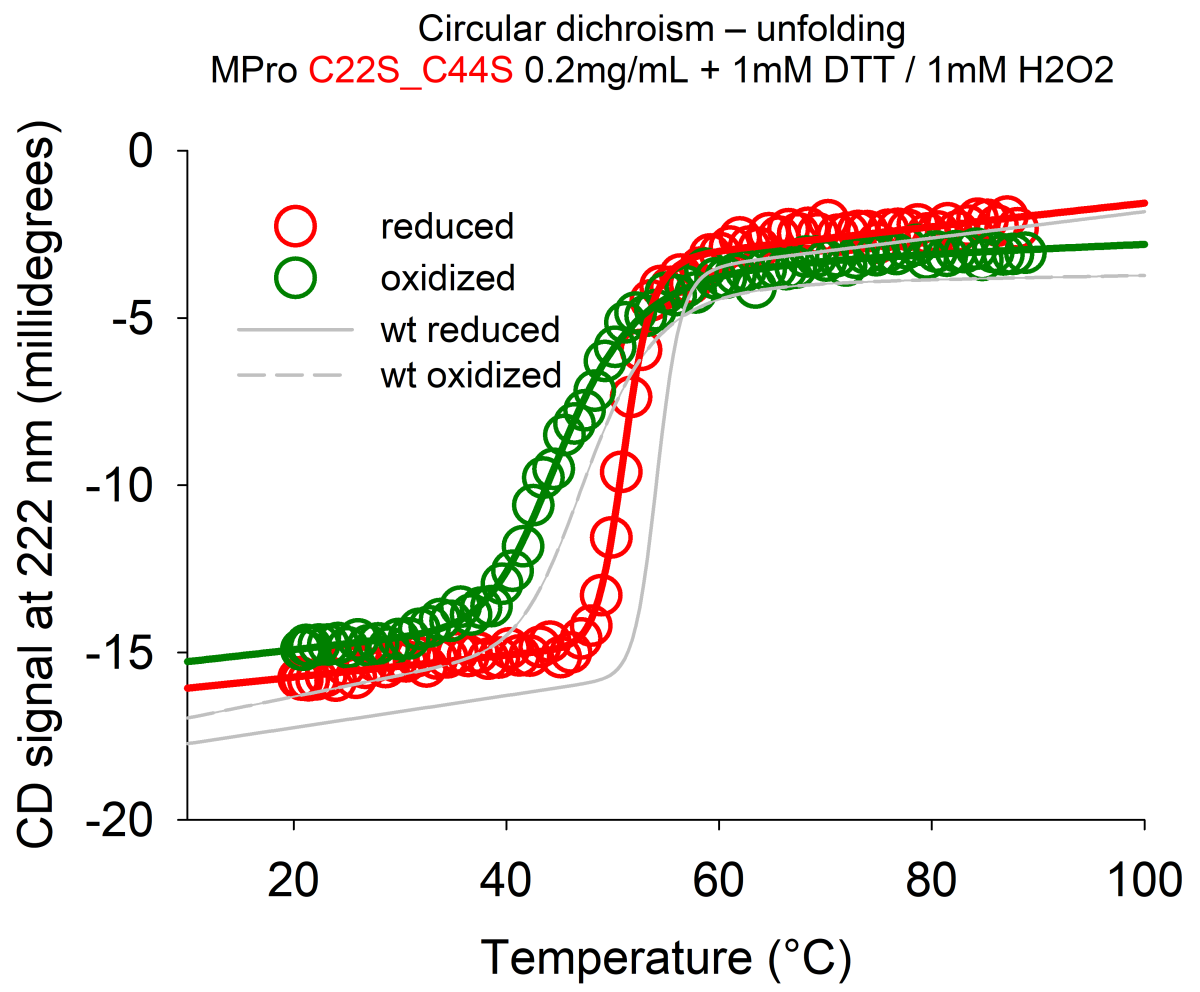
Circular dichroism – unfolding
MPro C22S_C44S 0.2mg/mL + 1mM DTT / 1mM H2O2
reduced
oxidized
wt reduced
wt oxidized
CD signal at 222 nm (millidegrees)
0
-5
-10
-15
-20
20
40
60
80
100
Temperature (°C)

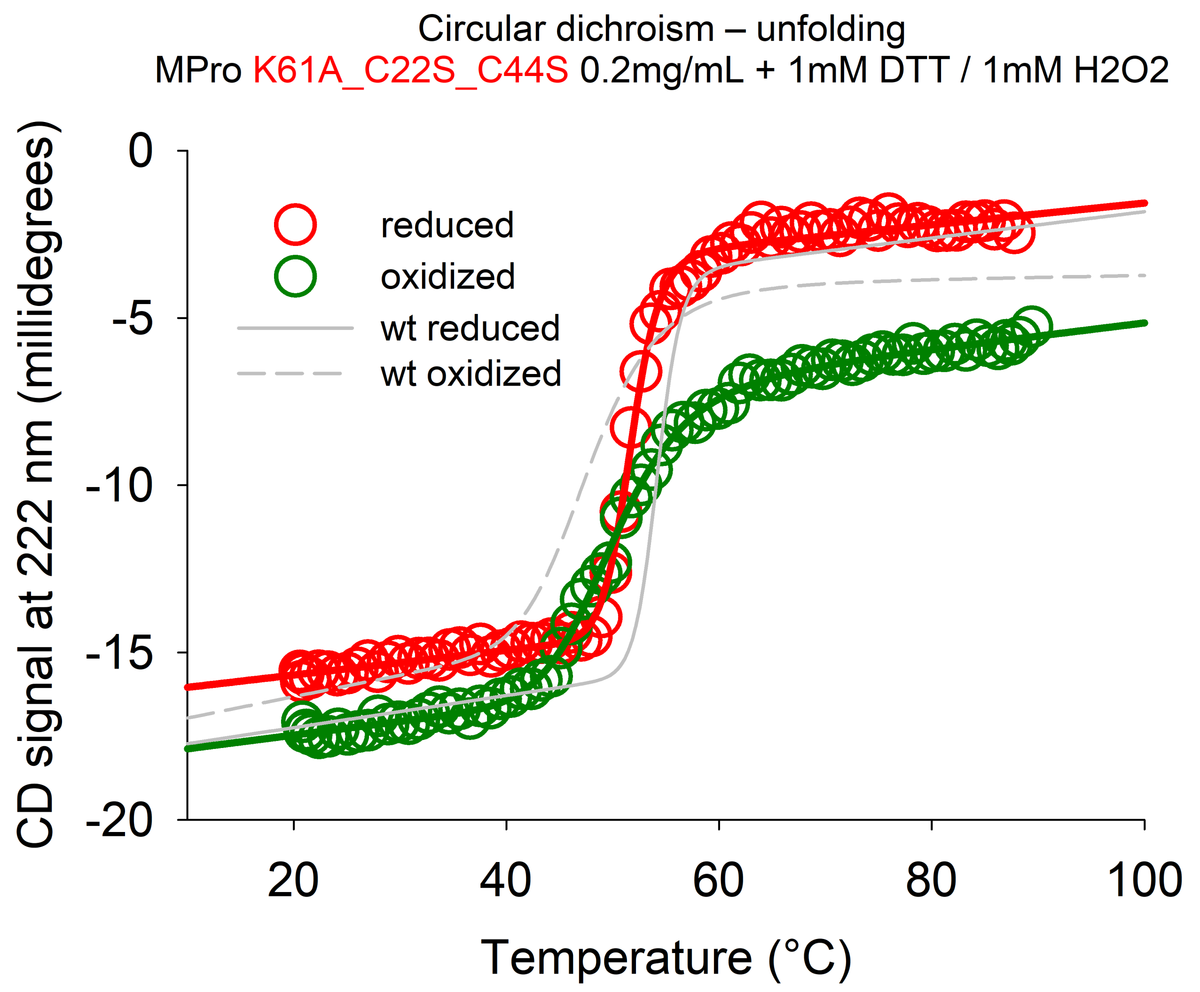

Circular dichroism – unfolding
MPro K61A_C22S_C44S 0.2mg/mL + 1mM DTT / 1mM H2O2
reduced
oxidized
wt reduced
wt oxidized
CD signal at 222 nm (millidegrees)
0
-5
-10
-15
-20
20
40
60
80
100
Temperature (°C)

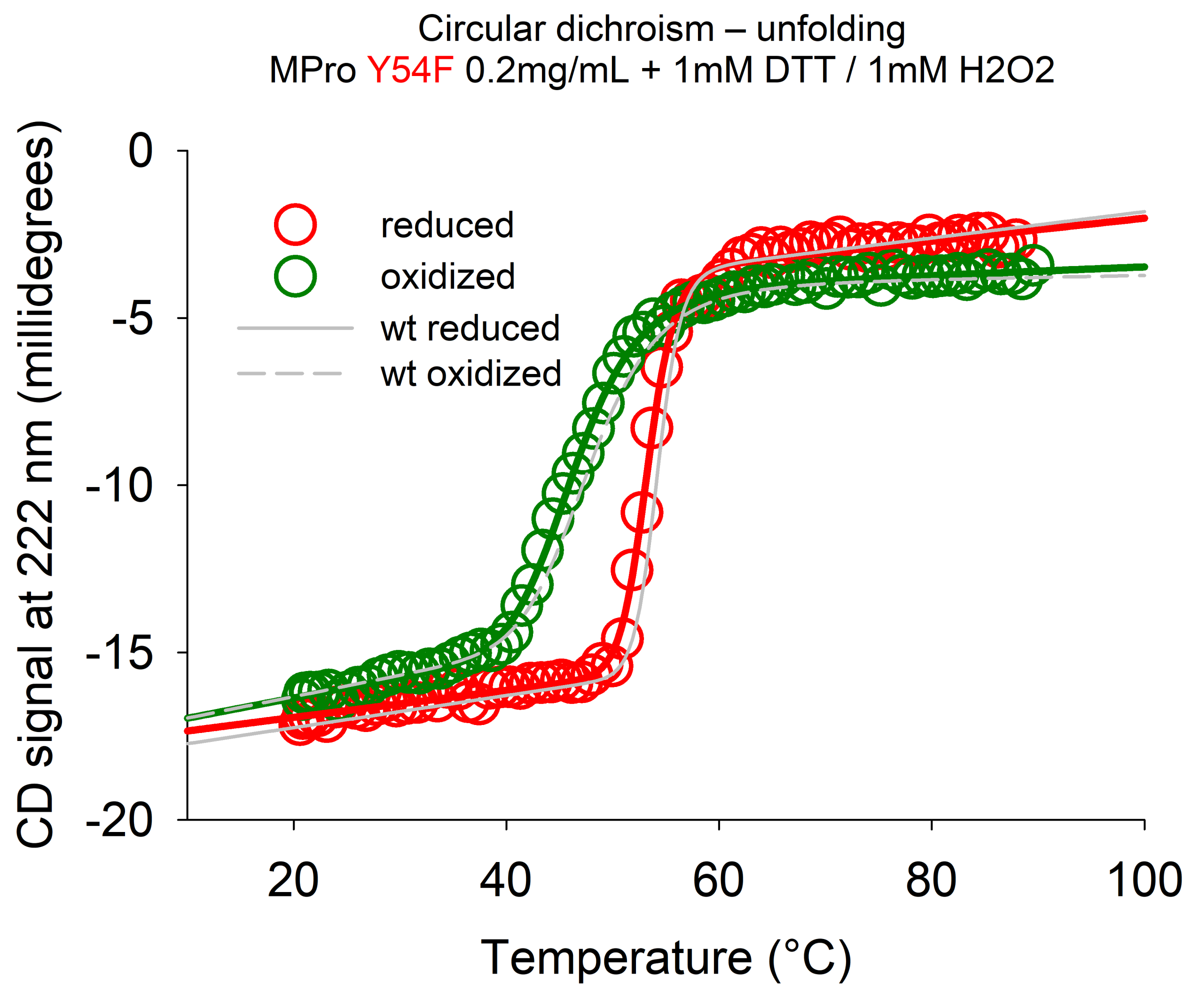
Circular dichroism – unfolding
MPro Y54F 0.2mg/mL + 1mM DTT / 1mM H2O2
reduced
oxidized
wt reduced
wt oxidized
CD signal at 222 nm (millidegrees)
0
-5
-10
-15
-20
20
40
60
80
100
Temperature (°C)
