# Supplementary material for: Multiple redox switches of the SARS-CoV-2 main protease in vitro provide opportunities for drug design
Source: Nat Commun. 2024 Jan 9;15:411. doi: 10.1038/s41467-023-44621-0 (PMC10776599; doi:10.1038/s41467-023-44621-0)
Supplement: Supplementary file 7 — Supplementary Data 4 [file 41467_2023_44621_MOESM7_ESM.pdf]

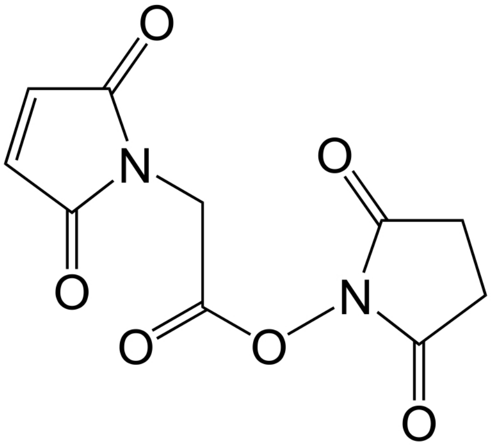

MAH

(Maleimidoacetic acid N-hydroxysuccinimide ester)

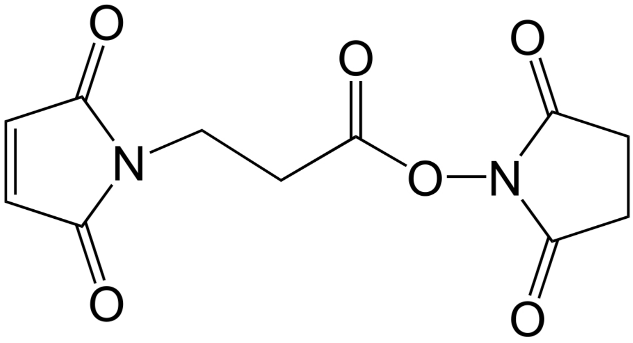

MPH

(3-(Maleimido)propionic acid N-hydroxysuccinimide ester)

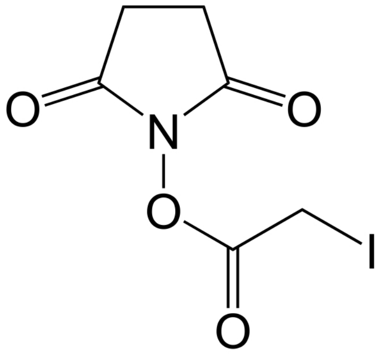

IANH

(Iodoacetic acid N-hydroxysuccinimide ester)

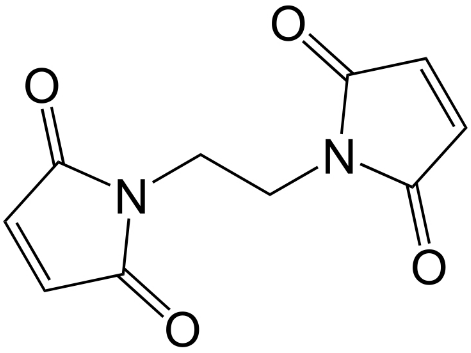

BMOE

(bis-maleimidoethane)

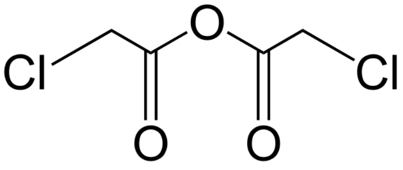

CAH

(Chloroacetic anhydride)

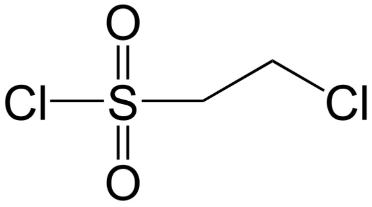

CSC

(2-Chloroethanesulfonyl chloride)

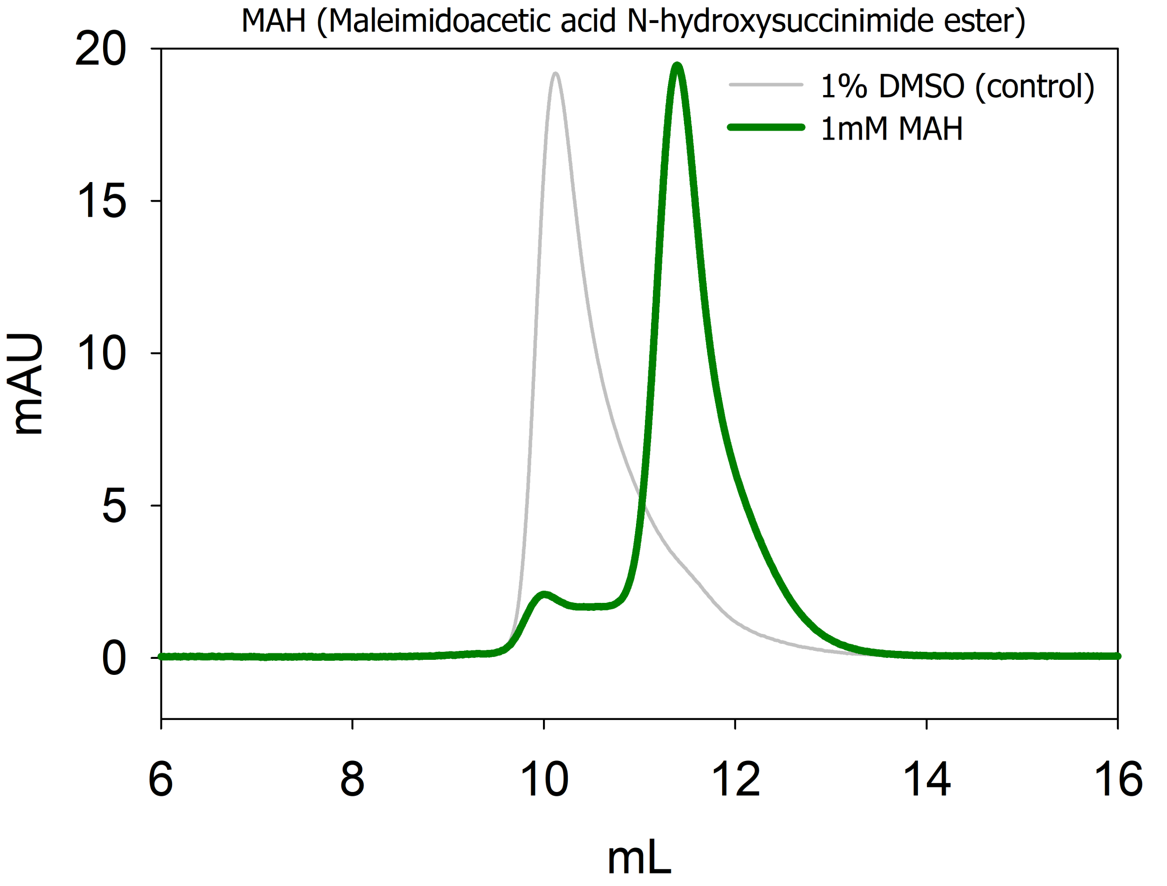
MAH (Maleimidoacetic acid N-hydroxysuccinimide ester)
1% DMSO (control)
1mM MAH
mAU
20
15
10
5
0
6
8
10
12
14
16
mL

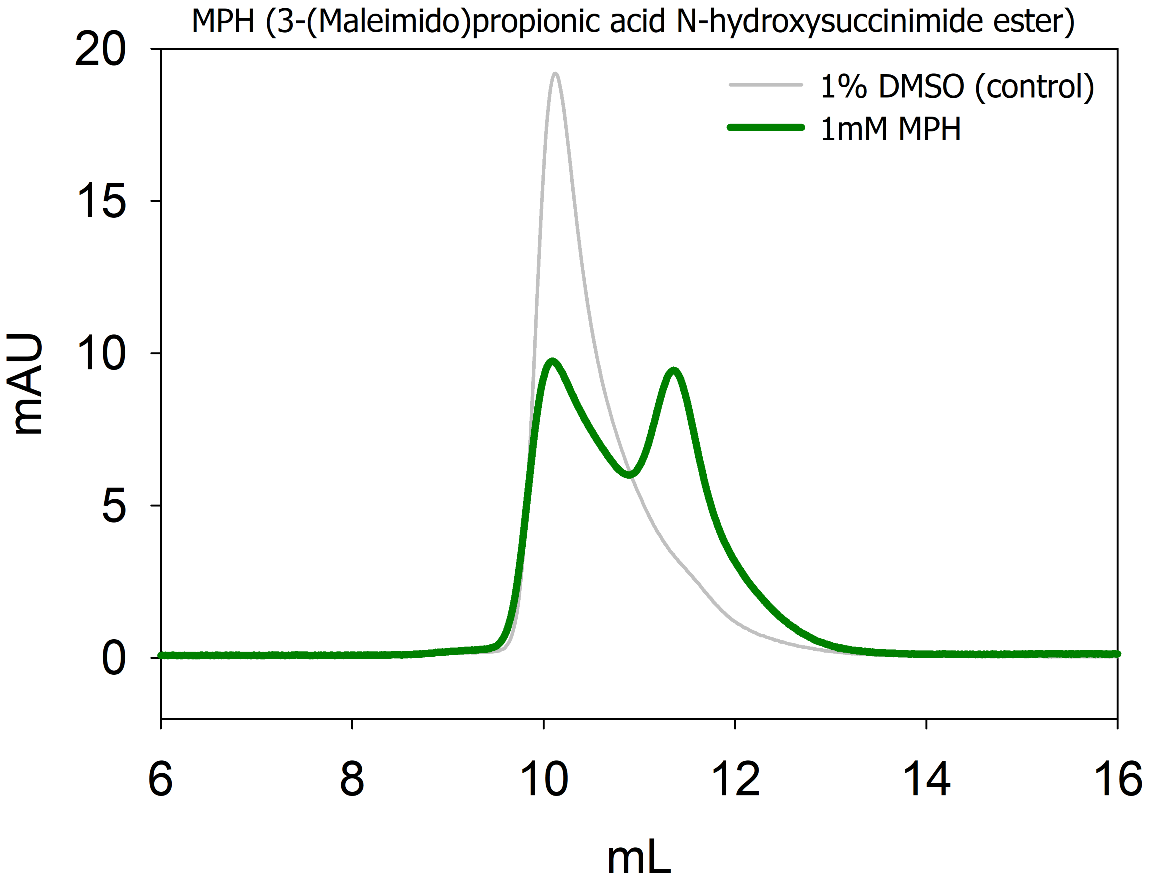
MPH (3-(Maleimido)propionic acid N-hydroxysuccinimide ester)
1% DMSO (control)
1mM MPH
mAU
20
15
10
5
0
6
8
10
12
14
16
mL

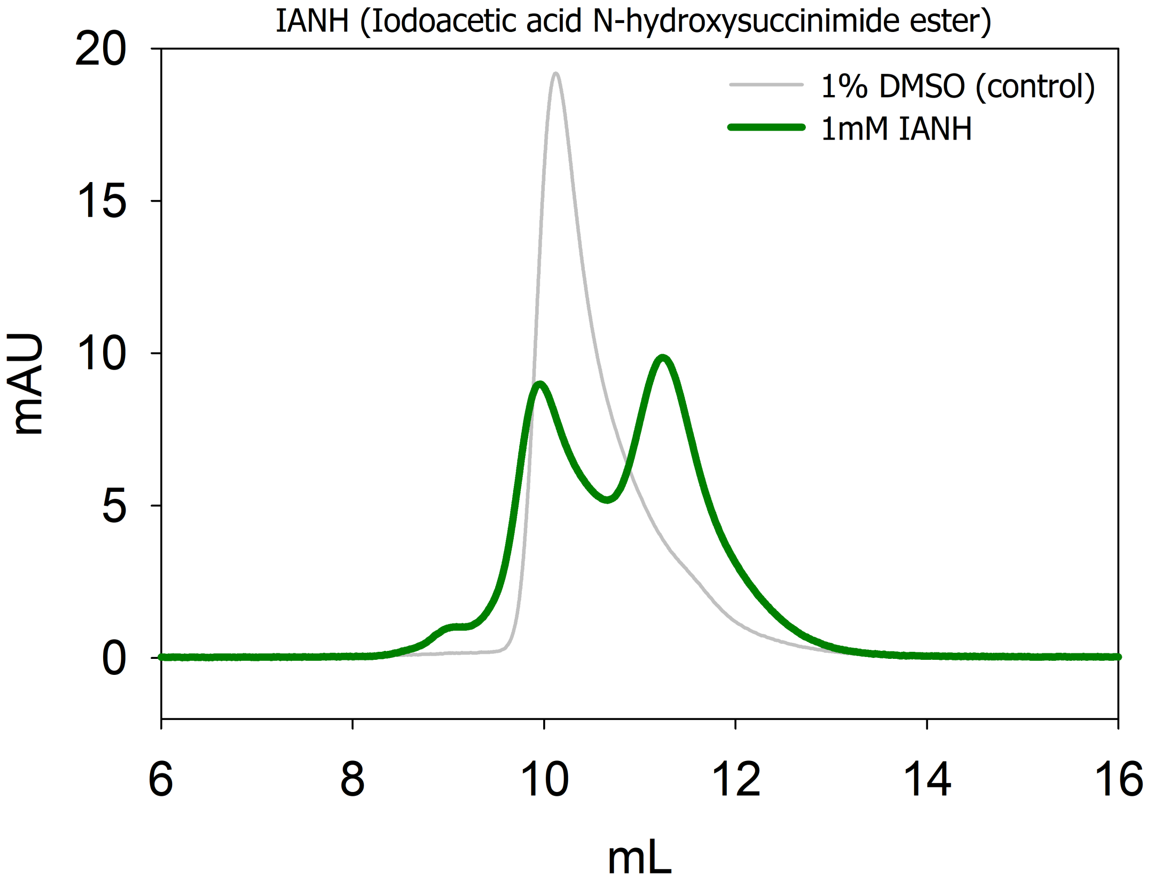
IANH (Iodoacetic acid N-hydroxysuccinimide ester)
1% DMSO (control)
1mM IANH
mAU
20
15
10
5
0
6
8
10
12
14
16
mL

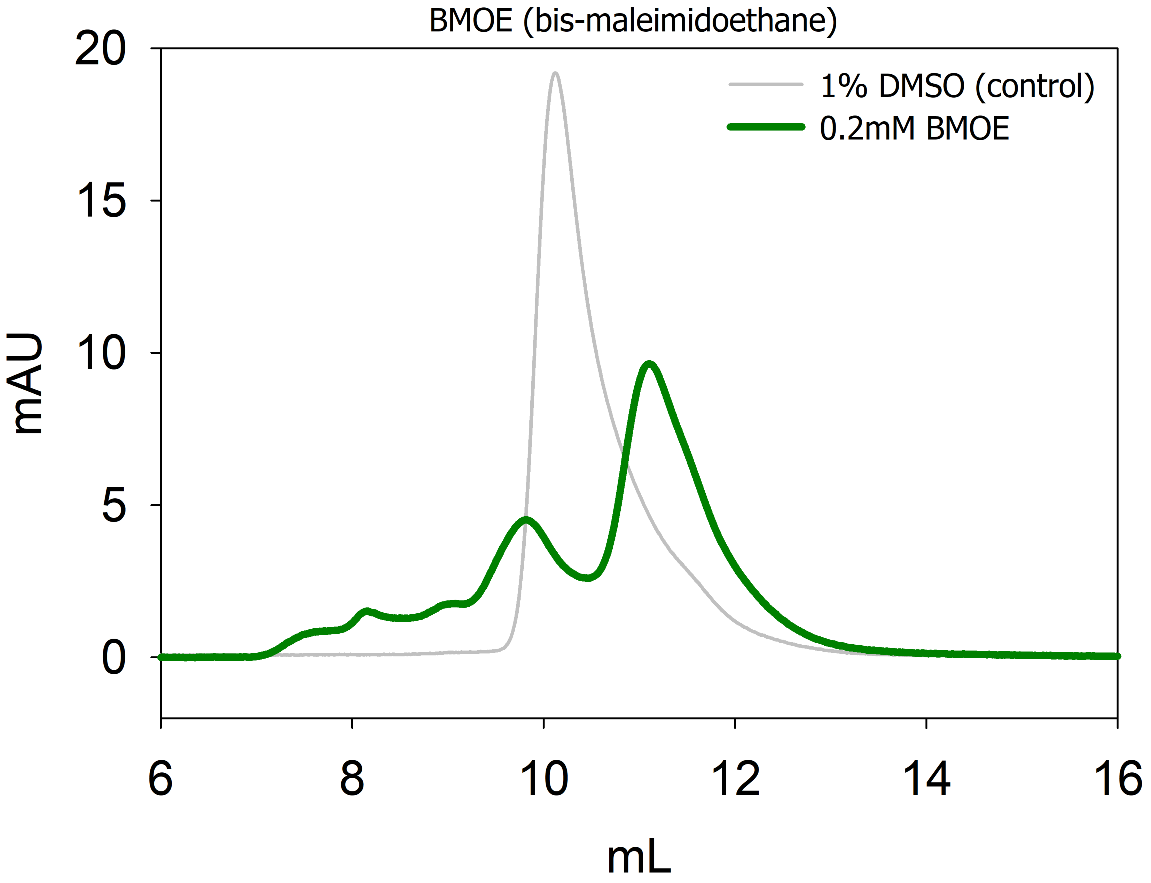
BMOE (bis-maleimidoethane)
1% DMSO (control)
0.2mM BMOE
mAU
20
15
10
5
0
6
8
10
12
14
16
mL

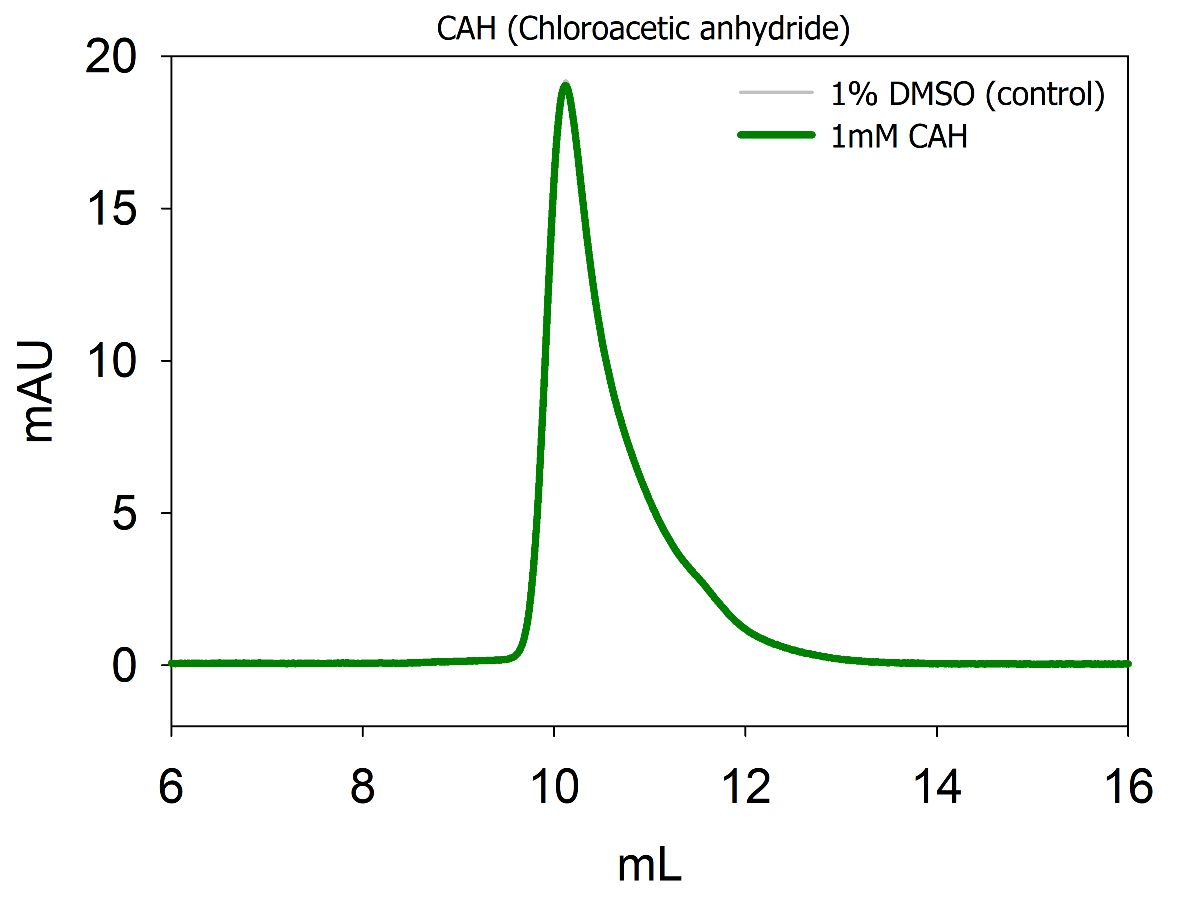
CAH (Chloroacetic anhydride)
20
15
10
5
0
mAU
1% DMSO (control)
1mM CAH
6
8
10
12
14
16
mL

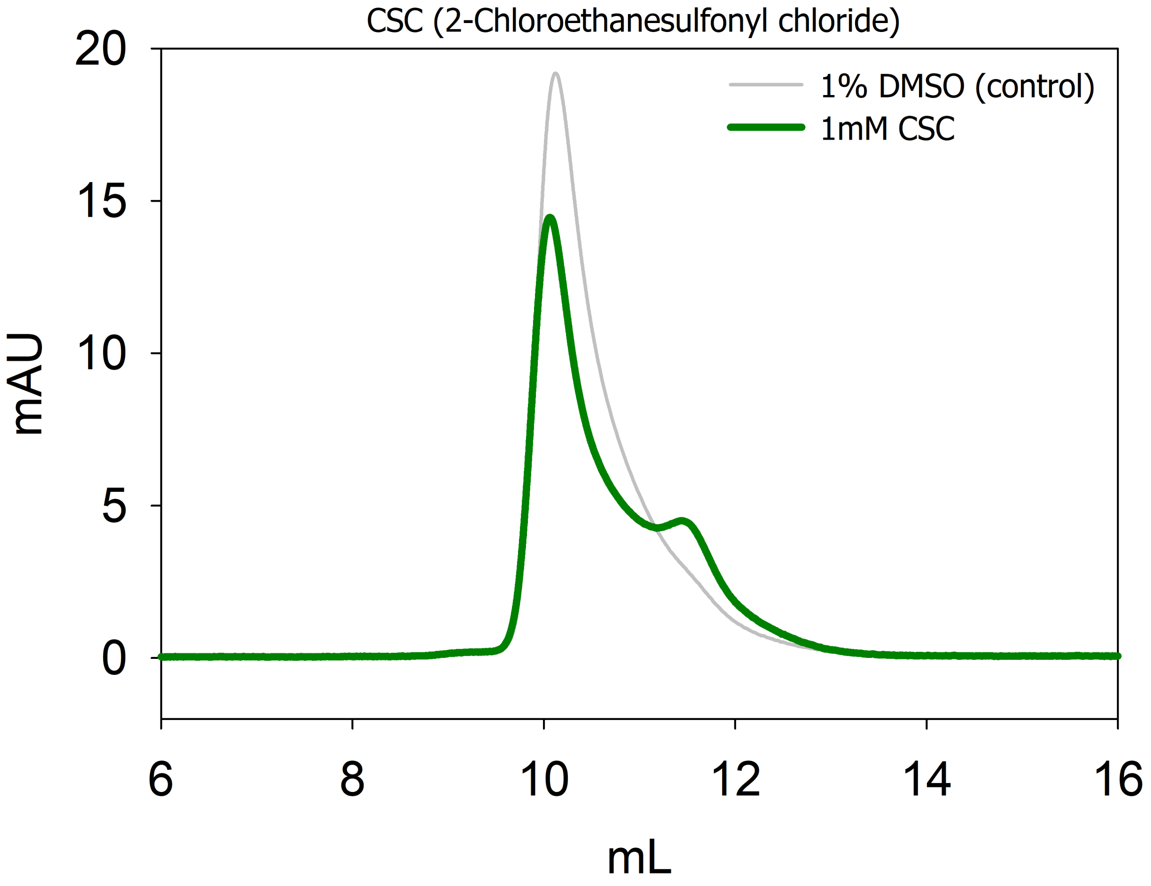
CSC (2-Chloroethanesulfonyl chloride)
1% DMSO (control)
1mM CSC
mAU
20
15
10
5
0
6
8
10
12
14
16
mL
