# Supplementary material for: Multiple redox switches of the SARS-CoV-2 main protease in vitro provide opportunities for drug design
Source: Nat Commun. 2024 Jan 9;15:411. doi: 10.1038/s41467-023-44621-0 (PMC10776599; doi:10.1038/s41467-023-44621-0)
Supplement: Supplementary file 8 — Reporting Summary [file 41467_2023_44621_MOESM8_ESM.pdf]

nature portfolio

Corresponding author(s): Tittmann, Kai

Last updated by author(s): Dec 11, 2023

Reporting Summary

Nature Portfolio wishes to improve the reproducibility of the work that we publish. This form provides structure for consistency and transparency in reporting. For further information on Nature Portfolio policies, see our Editorial Policies and the Editorial Policy Checklist.

Statistics

For all statistical analyses, confirm that the following items are present in the figure legend, table legend, main text, or Methods section.

n/a	Confirmed	
[ ]	[x]	The exact sample size (*n*) for each experimental group/condition, given as a discrete number and unit of measurement
[ ]	[x]	A statement on whether measurements were taken from distinct samples or whether the same sample was measured repeatedly
[ ]	[x]	The statistical test(s) used AND whether they are one- or two-sided<br>*Only common tests should be described solely by name; describe more complex techniques in the Methods section.*
[x]	[ ]	A description of all covariates tested
[x]	[ ]	A description of any assumptions or corrections, such as tests of normality and adjustment for multiple comparisons
[ ]	[x]	A full description of the statistical parameters including central tendency (e.g. means) or other basic estimates (e.g. regression coefficient) AND variation (e.g. standard deviation) or associated estimates of uncertainty (e.g. confidence intervals)
[x]	[ ]	For null hypothesis testing, the test statistic (e.g. *F*, *t*, *r*) with confidence intervals, effect sizes, degrees of freedom and *P* value noted<br>*Give P values as exact values whenever suitable.*
[x]	[ ]	For Bayesian analysis, information on the choice of priors and Markov chain Monte Carlo settings
[x]	[ ]	For hierarchical and complex designs, identification of the appropriate level for tests and full reporting of outcomes
[x]	[ ]	Estimates of effect sizes (e.g. Cohen's *d*, Pearson's *r*), indicating how they were calculated

Our web collection on statistics for biologists contains articles on many of the points above.

Software and code

Policy information about availability of computer code

Data collection	Kinetics and spectroscopic analysis<br>Chirascan Spectrometer Control Panel Application version 4.2.27 (Circular Dichroism)<br>Spectra Manager version 2.07.02 (Build 4) (UV-Vis-based Kinetics)<br><br>X-ray crystallography<br>mxCuBE 2 (X-ray data collection at P14 DESY/EMBL, Hamburg, Germany)<br><br>Mass spectrometry<br>Fusion Lumos Tune 3.4.3072.18<br>Xcalibur 4.4.16.14<br>Foundation 3.1 SP7QF1 build 306, SII 1.5<br>(all Thermo Fisher Scientific)<br><br>Analytical ultracentrifugation<br>ProteomeLab XL-I, GUI 6.0 (Firmware 5.7)<br><br>Electronic structure calculations<br>Gaussian16-A.03

Microscopy
AxioVision Zen 2 (version 2.0.0.0), from Zeiss

Western Blot
Image LabTM (version 5.2.1), from Bio-Rad

Data analysis

Kinetics and spectroscopic analysis
Pro-Data Viewer version 4.2.27 (Circular Dichroism)
Spectra Manager version 2.07.02 (Build 4) (UV-Vis-based Kinetics)
SigmaPlot version 11.0 (Circular Dichroism, UV-Vis-based Kinetics)

Crystallography
XDS VERSION Mar 15, 2019 BUILT=20190315 (X-ray, data processing)
XSCALE VERSION Jan 31, 2020 BUILT=20200417 (X-ray, data scaling)
autoPROC version 1.05 und 1.1.7 (data processing)
STARANISO version 2.3.59 und 2.3.74 (data processing)
CCP4 version 7.0.78 (X-ray, processing and refinement)
phenix.refine version 1.13_2998 (structure, refinement)
COOT version 0.8.9.2 (structure, model building)
MolProbity-Server Version 4 (structure validation)
The PyMOL Molecular Graphics System version 1.3 (structure representation)

Electronic structure calculations
Amber20
AmberTools v20.15
Python3.7 (libraries numpy and matplotlib)

Mass spectrometry data analysis
MaxQuant version 2.0.3.0 (MPI for Biochemistry, Planegg, Germany)

Analytical ultracentrifugation
SEDFIT version 16.2b (fitting of the raw data with the Continuous c(s) Distribution model)
SEDNTERP version 1.09 (calculation of extinction coefficients, partial specific volumes, density, viscosity, s 20, w correction)
GUSSI version 1.4.6 (preparation of figures)

Phylognetic analysis
MAFFT 7
iTOL 5
Jalview 2.11

Western Blot
Prism (version 9.0.0), from GraphPad

For manuscripts utilizing custom algorithms or software that are central to the research but not yet described in published literature, software must be made available to editors and reviewers. We strongly encourage code deposition in a community repository (e.g. GitHub). See the Nature Portfolio guidelines for submitting code & software for further information.

Data

Policy information about availability of data
All manuscripts must include a data availability statement. This statement should provide the following information, where applicable:
- Accession codes, unique identifiers, or web links for publicly available datasets
- A description of any restrictions on data availability
- For clinical datasets or third party data, please ensure that the statement adheres to our policy

The refined structural protein models and corresponding structure-factor amplitudes have been deposited under PDB accession codes 7ZB6 [http://doi.org/10.2210/pdb7ZB6/pdb] (Mpro C44S), 7ZB7 [http://doi.org/10.2210/pdb7ZB7/pdb] (Mpro Y54F) and 7ZB8 [http://doi.org/10.2210/pdb7ZB8/pdb] (Mpro K61A). The structures cited in this publication are available under their respective PDB accession codes 6LU7 [http://doi.org/10.2210/pdb6LU7/pdb], 7JR4 [http://doi.org/10.2210/pdb7JR4/pdb] and 7KPH [http://doi.org/10.2210/pdb7KPH/pdb]. Source data are provided for the kinetic analysis of enzyme activity (SARS-CoV-2 Mpro wild-type and variants, SARS-CoV Mpro wild-type) as well as all Western blots. Data of the quantum chemical calculations and MD simulations are provided in a public repository (https://doi.org/10.25625/GBIC2M). All other data are available on request.

Research involving human participants, their data, or biological material

Policy information about studies with human participants or human data. See also policy information about sex, gender (identity/presentation), and sexual orientation and race, ethnicity and racism.

Reporting on sex and gender	n.a.
Reporting on race, ethnicity, or other socially relevant groupings	n.a.
Population characteristics	n.a.
Recruitment	n.a.
Ethics oversight	n.a.

Note that full information on the approval of the study protocol must also be provided in the manuscript.

Field-specific reporting

Please select the one below that is the best fit for your research. If you are not sure, read the appropriate sections before making your selection.

[x] Life sciences [ ] Behavioural & social sciences [ ] Ecological, evolutionary & environmental sciences

For a reference copy of the document with all sections, see nature.com/documents/nr-reporting-summary-flat.pdf

Life sciences study design

All studies must disclose on these points even when the disclosure is negative.

Sample size	no sample size calculation was performed, all biophysical experiments were conducted with three statistical replicates
Data exclusions	no data were excluded from analysis
Replication	Experiments were performed in at least two independent biological replicates given virtually identical results
Randomization	n.a. as all data were included in the statistical analysis
Blinding	n.a.as all data were included in the statistical analysis

Reporting for specific materials, systems and methods

We require information from authors about some types of materials, experimental systems and methods used in many studies. Here, indicate whether each material, system or method listed is relevant to your study. If you are not sure if a list item applies to your research, read the appropriate section before selecting a response.

Materials & experimental systems

n/a	Involved in the study	
[ ]	[x]	Antibodies
[ ]	[x]	Eukaryotic cell lines
[x]	[ ]	Palaeontology and archaeology
[ ]	[x]	Animals and other organisms
[x]	[ ]	Clinical data
[x]	[ ]	Dual use research of concern
[x]	[ ]	Plants

Methods

n/a	Involved in the study	
[x]	[ ]	ChIP-seq
[x]	[ ]	Flow cytometry
[x]	[ ]	MRI-based neuroimaging

Antibodies

Antibodies used

1. SARS-CoV-2 Spike (GeneTex, Cat# 632604)
2. SARS-CoV-2 Nucleoprotein (Sino Biological, Cat# 40143-R019)
3. GAPDH (abcam, Cat# ab8245)
4. Alexa Fluor 488 donkey anti-mouse (Thermo Fisher Scientific, Cat# A21202)
5. Alexa Fluor 546 donkey anti-rabbit (Thermo Fisher Scientific, Cat# A10040)
6. Donkey anti-mouse IgG, HRP conj. (Jackson ImmunoResearch, Cat#715036150)

	7. Donkey anti-rabbit IgG, HRP conj. (Jackson ImmunoResearch, Cat#711036152)<br>8. anti-dimedone antibody (Pineda antibodies, Berlin, Germany)
Validation	Validation of all antibodies is provided on the corresponding manufacturers' websites.

Eukaryotic cell lines

Policy information about cell lines and Sex and Gender in Research

Cell line source(s)	Vero E6 (Vero C1008), Monkey, from ATCC, Cat# CRL-1586
Authentication	The cell line was authenticated by the Leibniz-Institute DSMZ (German Collection of Microorganisms and Cell Cultures) in February 2021. DNA profiling was performed using 17 different and highly polymorphic STR (Short Tandem Repeat) loci. In addition, we have tested your human samples for the presence of mitochondrial DNA sequences from rodent cells such as mouse, rat, Chinese and Syrian hamster. Animal cell line samples have been subjected to the procedure of Cytochrome C Subunit I (COI) DNA Barcoding for identification of the species
Mycoplasma contamination	Cells were routinely tested and ensured to be negative for mycoplasma contamination.
Commonly misidentified lines<br>(See ICLAC register)	No commonly misidentified cell lines were used in the study.

Animals and other research organisms

Policy information about studies involving animals; ARRIVE guidelines recommended for reporting animal research, and Sex and Gender in Research

Laboratory animals	SARS-CoV-2 ‘wildtype’, Göttingen/Germany, isolated from patient (Stegmann et al., 2021)
Wild animals	n.a.
Reporting on sex	n.a.
Field-collected samples	n.a.
Ethics oversight	n.a.

Note that full information on the approval of the study protocol must also be provided in the manuscript.

Plants

Seed stocks	n.a.
Novel plant genotypes	n.a.
Authentication	n.a.
